# Tandem Mass Spectrometric Analysis and Cross-Species Comparison of Triacylglycerol Regioisomers in Mammalian Milk

**DOI:** 10.1021/acs.jafc.5c08520

**Published:** 2026-01-05

**Authors:** Qizhu Zhao, Mikael Fabritius, Marika Kalpio, Md Abdullah Al Sazzad, Baoru Yang

**Affiliations:** Food Sciences Unit, Department of Life Technologies, Faculty of Technology, 8058 University of Turku FI-20014 Turku, Finland

**Keywords:** fatty acid, mammalian milk fat, regioisomers, *sn*-position, tandem mass spectrometry, triacylglycerol

## Abstract

Milk fats have complex profiles of triacylglycerols (TG) with a significant gap in understanding the species-specific compositions of regioisomers. This study comprehensively analyzed TG regioisomers in milk of eight mammalian species using ultra-high performance liquid chromatography-electrospray ionization tandem mass spectrometry and a calculation algorithm. Distinct TG regioisomer profiles were observed across species, shaped by the phylogenetic status and the structure of digestive tracts (ruminants, pseudo-ruminants, and non-ruminants). The ruminants (cow, goat, and sheep) exhibited highly similar milk TG regioisomeric profiles, while the pseudo-ruminant (camel) showed similar profiles to those of the non-ruminants (dog, horse, human, and pig). The non-ruminants showed a *sn*-2 abundance of palmitic acid in milk TGs; especially dog and pig milk displayed TG regioisomer profiles close to that of human milk. These findings provide novel insights into cross-species variation in lipid metabolism and the nutritional properties of milk fats, supporting product development for food and early life nutrition.

## Graphical abstract

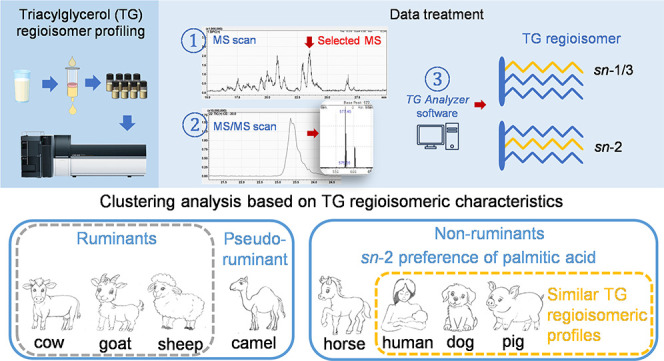

## 1 Introduction

Mammalian milk fat is an essential component of the diet and nutrition for newborns, providing energy and other essential bioactive compounds to support cellular functions and facilitate the absorption of fat-soluble nutrients.^1,2^ Among key components, triacylglycerols (TG) represent up to 98% of mammalian milk fats.^3,4^ The positional distribution of fatty acids (FAs) on the glycerol backbone has a significant impact on digestion, absorption, and metabolism of milk fat TGs in newborns.^5–7^ To mimic the nutritional benefits of human milk, infant formulas should ideally contain TGs with similar regioisomeric structures. However, the fats in most commercial infant formulas are derived from plant oils, which have different TG structures to those found in human milk fat.^8^ This raises the question of whether the TG regioisomer composition of milk from other mammalian species more closely resembles that of human milk.

While the FA composition of milk TGs has been extensively studied across various mammalian species,^9–17^ including double bond positional and geometric isomers,^17^ much less is known about the TG regioisomers. Overall FA compositions of *sn*-1,3 and *sn*-2 positions (*sn*, stereospecific numbering) can be analyzed after enzymatic hydrolysis, but this methodology does not provide information on the regioisomer composition of individual TG molecules.^18^ Currently, most of the studies related to TGs in mammalian milk are mainly focused on the level of TG species and TG molecular species without the *sn*-positional information on FAs.^3,19–24^ A few studies have provided TG regioisomer information on a limited number of specific TG molecular species from milk of the *Bovidae* (cow, buffalo, goat, sheep, and yak), *Equidae* (donkey), and *Camelid* (camel) families.^11,25–28^ Therefore, investigating the regioisomer composition of TGs and comparing them between different species would provide deeper insights than overall TG or FA profiles, enhancing our understanding of species-specific lactation biochemistry and lipid secretion mechanisms. Progress in this field of research has historically been hindered by the limited availability of reference compounds and the technical complexity involved in accurately resolving and quantifying TG regioisomers.

To address the challenge, our group recently developed fragmentation models based on collision-induced dissociation (CID) of TG ammonium adducts in electrospray ionization (ESI) tandem mass spectrometry (MS/MS), calibrated with a wide range of regiopure TG standards.^29^ This, together with calculation software, enables calculation of TG regioisomer ratios also for those TGs for which the regiopure reference standards are not available. The model was later updated with additional TGs containing short- and medium-chain FAs.^30^ In addition to the fragmentation model, the calculation software has a unique optimization algorithm that mitigates the effects of isobaric overlap of fragments from multiple different TG molecular species. In practice, using the fragmentation patterns in the model, the algorithm looks at the measured fragment spectra and tests all possible TG regioisomer combinations, including multiple different molecular species, and will report the combination of TG regioisomers that produce the closest matching synthetic spectra.

In this study, we applied this methodology to analyze and compare the TG regioisomer profiles in milk samples from eight mammalian species. The aim is to provide comprehensive knowledge of interspecies similarities and differences among mammalian milk fat in TG structural profiles at a regiospecific level. Such knowledge is crucial for understanding the nutritional properties of milk fats and their potential application in next-generation human milk fat substitutes and formulas for pets.

## 2 Materials and Methods

### 2.1 Nomenclature

The nomenclature follows the LIPID MAPS guideline.^31^ Briefly, annotations are presented at three structural levels. TG species provides the total number of acyl carbons (ACNs) and the total number of double bonds (DBs) within TG molecules (e.g., TG 48:1). TG molecular species has a known FA composition but no information on FA positional distribution (e.g., TG 16:0_16:0_16:1). TG regioisomers have FAs identified at *sn*-2 vs. *sn*-1,3 positions (e.g., TG 16:0_16:0­(*sn*-2)_16:1).

### 2.2 Materials and Reagents

Milk samples of eight mammalian species were studied, covering six families from four orders. *Artiodactyla* order animals included (family in brackets) camel (*Camelidae*), cow, goat, and sheep (*Bovidae*), and pig (*Suidae*); the *Perissodactyla* order included horse (*Equidae*); the *Primates* order included human (*Hominidae*), and the *Carnivora* order included dog (*Canidae*). The eight species belonged to three distinct groups based on their digestive systems: the pseudo-ruminant (camel), ruminants (cow, goat, and sheep), and non-ruminants (dog, horse, human, and pig).

The sample collection was strictly non-invasive and did not involve any dietary or life style interventions on humans or animals. Milk samples from camel (mature, pooled sample, approximately 40 two-humped camels, Urumqi, China), cow (K-Citymarket, Turku, Finland), goat (K-Citymarket, Turku, Finland), horse (17 years old, mature, Kylämäen Hevostila), and sheep (2 years and 3 months old, colostrum, Lammastila SikkaTalu) were obtained from commercial sources or farms where milk was produced as a routine agro-food practice. Dog (2 years and 2 months old, mature, lagotto romagnolo, Kennel Nuxo) and pig (4 years and 9 months old, colostrum, Jonna Lehtinen) milk samples were donated from the owners of the animals from the surplus portion of a natural overproduction of milk without causing harm or distress to the animals or their offspring. The human milk study was approved by The Ethical Committee of the Wellbeing Services County of Southwest Finland (ETMK Dnro: 106/1801/2018). The mature milk sample was voluntarily donated by a healthy breastfeeding Finnish mother (38 years old) of a male infant with informed written consent for the use of the sample in research. The gestational age was 38 weeks + 2 days (early term), and the parity was 2 (multiparous). No personal identifiers were recorded, and the sample was anonymized prior to analysis.

FA methyl ester (FAME) mixture GLC reference standard 566C was purchased from Nu-Chek-Prep (Elysian, MN, USA) and Supelco 37 Component FAME Mix from Supelco (St. Louis, MO, USA) as external standards. TG internal standard triheneicosanoin (TG 21:0/21:0/21:0) was purchased from Larodan (Malmö, Sweden). Acetyl chloride (CH_3_COCl, ≥ 99.0%), chloroform (CHCl_3_, HPLC grade, ≥ 99.8%), and methanol (MeOH, LC–MS grade, ≥ 99.9%) were purchased from Sigma-Aldrich (St. Louis, MO, USA). Diethyl ether (HPLC grade, ≥ 99.9%) and hexane (HPLC grade, ≥ 97%) were purchased from Fisher Scientific (Loughborough, UK). Potassium chloride (KCl) was purchased from VWR International (Radnor, PA, USA), and potassium carbonate (K_2_CO_3_) was from Acros Organics (Geel, Belgium). Ammonium acetate (CH_3_CO_2_NH_4_) was purchased from Merck (Germany). 2-Propanol (IPA, LC–MS grade, ≥ 99.9%) was purchased from VWR International Oy (Finland).

### 2.3 Extraction and Fractionation of Triacylglycerols

Mammalian milk TGs were extracted with a modified Folch method.^32^ All evaporation and storage steps were performed under nitrogen to prevent oxidative degradation. Briefly, 500 mg of milk sample was weighed into a reusable glass tube and the weight recorded, followed by the addition of 1.5 mL MeOH, 2.5 mL CHCl_3_, and 0.8 mL 0.88% KCl (*w*/*v*), vortexing briefly after each addition. The sample was then centrifuged at 1100*g* for 5 min, and the lower phase was collected into a single-use tube. An additional 1.5 mL of CHCl_3_ was added to the upper phase for the second extraction. After centrifugation, the lower phase was collected and combined with the lower phase from the first extraction. Thereafter, the solvent was evaporated to dryness under a gentle nitrogen flow at 50 °C. The total lipids were redissolved in 1 mL of CHCl_3_ and stored at −80 °C until further TG isolation by solid-phase extraction (SPE).

The SPE extraction was performed using a Sep-Pak Vac silica 1 cc (500 mg) SPE column (Waters, Dublin, Ireland). The column was conditioned first with 1 mL of hexane: diethyl ether (1:1, v/v), and then about 15 mg of total lipid sample in CHCl_3_ was applied to the column. The TG fractions were eluted with 9 mL of hexane: diethyl ether (1:1, v/v). Then, the solution was evaporated to dryness under a gentle nitrogen flow at 50 °C. TG fractions were dissolved in 1 mL of hexane and stored at −80 °C prior to MS/MS analysis. TG extraction and fractionation were performed in duplicate for each milk sample.

### 2.4 Fatty Acid Composition Analysis

FA composition of TG fractions was analyzed as FAMEs using a Shimadzu GC-2010 gas chromatograph (GC) with AOC-20i auto injector and flame ionization detector (FID) (Shimadzu Corporation, Kyoto, Japan). FAMEs were prepared by the acid-catalyzed method.^8^ A total of 1 mg of TG fractions dissolved in CHCl_3_ and 50 μg of internal standard TG 21:0/21:0/21:0 in CHCl_3_: MeOH (2:1, v/v) were pipetted into a sealed vial. The solvent was then evaporated to dryness under nitrogen. Subsequently, a volume of 2 mL of freshly prepared CH_3_COCl: MeOH (1:10, v/v) was added to the dried residue. Methylation was performed in an oven at 50 °C overnight. After the mixture was cooled, 2 mL of K_2_CO_3_ was gently added. Then, 1 mL of hexane was introduced. The mixture was vortexed thoroughly and centrifuged at 1000 g for 5 min. The top layer was collected into a 1.5 mL vial for GC-FID analysis. The methylation was performed in triplicate.

FAME37 and GLC-566C were used as external standard mixtures for identification. A wall-coated open tubular column DB-23 (60 m × 0.25 mm i.d., liquid film 0.25 μm, Agilent Technologies, J.W. Scientific, Santa Clara, CA, USA) was used. Helium was used as the carrier gas. The injection volume was 0.5 μL. Splitless injection mode was used, and the split was opened after 1 min. The column oven temperature program was according to a previous study with some modifications.^8^ The injection temperature was 90 °C and held for 1 min. It then increased to 170 °C at a rate of 6.5 °C/min, followed by an increase to 205 °C at 2.75 °C/min and held for 10 min, and finally increased to 230 °C at 2.85 °C/min and held for 2 min. The detector temperature was 280 °C. Correction factors were calculated with external standards to correct the difference in the detector response between each FA and the internal standard. Results were expressed as a weight percentage (%) of the total identified FAs.

### 2.5 Triacylglycerol Species and Regioisomer Identification

TG species and regioisomers were analyzed using a UHPLC Shimadzu LC–MS 8045 triple quadrupole MS/MS instrument with ESI operated in the positive mode. A Waters Cortecs C18 column (150 × 2.1 mm, 1.6 μm particle size) with a Waters VanGuard C18 precolumn (1.6 μm particle size) was used for the separation of TGs based on the method recently developed by our group.^30^

The mobile phase consisted of A: MeOH: H_2_O (1000:1, v/v) with CH_3_CO_2_NH_4_ (10 mM), and B: IPA: H_2_O (1000:1, v/v) with CH_3_CO_2_NH_4_ (10 mM). The gradient program started at 99% solvent A, decreased linearly to 70% over 30 min, and then linearly to 50% within 7 min, followed by an isocratic hold for 1 min. It further decreased to 30% in 2 min, returned to 99% in 4 min, and was held for up to 50 min. The flow rate was 0.2 mL/min until 44 min, then increased to 0.3 mL/min at 46 min, and maintained for the final 4 min. The total analysis time was 50 min. The column temperature was maintained at 60 °C.

The interface voltage, temperature, desolvation temperature, and DL temperature of the mass spectrometer were set to 4 kV, 300 °C, 526 °C, and 200 °C, respectively. Nebulizing, heating, and drying gas flows were 2, 15, and 5 L/min, respectively. MS scan in positive polarity *m*/*z* 200–1000 was used to identify TG species with distribution estimated from ammonium adduct ion peak areas. Following the identification of major TG species, product ion scans at *m*/*z* 50–700 of relevant precursors were performed using argon as collision gas at 30 eV to analyze regioisomer composition. TG fractions dissolved in hexane were first dried under nitrogen and then diluted to a final concentration of 20 μg/mL using IPA: hexane (4:1, v/v). For each milk sample, two extracted and fractionated TG samples were both analyzed in duplicate, for a total of four analyses per milk sample. The injection volume of samples was 1 μL.

### 2.6 Data Handling and Statistical Analysis

FA composition and MS and MS/MS raw data were first preprocessed and exported from LabSolutions software (Shimadzu Corporation, Kyoto, Japan). TG species distribution was estimated from the integrated peak areas of ammonium adduct ions. TG regioisomer results were analyzed using the TG Analyzer software published by our group.^29,30^ Briefly, the fragmentation model and optimization algorithm are two main features of the analysis logic. First, the chain length and degree of unsaturation of the *sn*-2 FA significantly affect the relative abundance of diacylglycerol (DG) fragment ions [M+NH_4_–RCO_2_H–NH_3_]^+^, whereas the *sn*-1,3 FAs have a comparatively minor impact. Utilizing a set of calibration standards, a fragmentation model can be established, estimating fragmentation patterns for TG regioisomers that are not included in the calibration data of the model. Currently, the software has been calibrated with 26 AAB-type regioisomer pairs and 7 ABC-type regioisomer triplets^29,30^ (Supplementary Table S1). The calibration also utilizes leave-one-out cross-validation (LOOCV), which produces the error rate of the calculations for TGs that are not part of the calibration data. In essence, for the LOOCV validation, the model was first calibrated with all TG standards except one. After that, the result was calculated for that one excluded standard. The LOOCV result shows the true performance of the model when that particular standard was not used for the calibration. This validation provides a more accurate estimation of the real performance of the model, as most TG regioisomers found in the samples are not included in the calibration data. The LOOCV results are shown in Supporting Information (Table S2 and Table S3).

In addition to the fragmentation model, the software has an optimization algorithm that resolves the effects of interference from isobaric DG fragments. First, the calculation algorithm identifies the FA acylium fragments [RCO]^+^. Based on the observed [RCO]^+^ fragments, the software creates a list of all possible TG molecular species combinations. Utilizing the fragmentation model, the algorithm then tests different concentrations of TG regioisomers within the identified TG molecular species to create synthetic fragment spectra that closely match with the observed spectra. As the optimization is performed simultaneously for all TG regioisomers within the fragment spectra, this allows calculation of TG regioisomer abundances even for molecular species that share the same isobaric DG fragments. An illustrative example of the optimization process was described in our previous study.^30^

All data were presented as the mean ± standard deviation. Statistical analyses were performed using IBM SPSS Statistics 29.0 (IBM Corp., Armonk, New York, USA). Data were first assessed for normality and homogeneity of variance. The significance of the difference among samples was tested by a one-way analysis of variance (ANOVA), followed by the post hoc Tukey’s HSD test. Statistical significance was set at *p* < 0.05.

Clustering analysis was performed using Python (version 3.8.3) with the Ward method and the Euclidean distance metric. Data visualization was carried out using Origin2016 (OriginLab Corporation, Northampton, MA, USA) and GraphPad Prism 10 (GraphPad Software, Boston, USA).

## 3 Results and Discussion

### 3.1 Fatty Acid Composition

The clustering of the FA composition of the TG fraction of the milk samples is shown in Figure S1. Pseudo-ruminant (camel), ruminant (cow, goat, and sheep), and horse milk had more similar FA distributions in TG fractions, which were distinguished from the FA profile of human, dog, and pig milk.

The detailed FA composition of the milk TG fractions is presented in Table S4. Oleic (18:1­(9Z), 18.9–38.5%) and palmitic acids (16:0, 19.9–35.2%) dominated in both ruminants’ and non-ruminants’ milk. Among ruminants except sheep milk, FA 16:0 was the predominant FA (25.5%–35.2%). Notably, sheep milk had the lowest FA 16:0 (21.9%), whereas FA 18:1­(9Z) (24.0%) was the most abundant one. A similar result has been reported previously: FAs 18:1­(9Z) and 16:0 both dominated within the range of 23.9–25.6% of total FAs in the milk fat of three sheep breeds.^33^ In contrast, the milk TGs of non-ruminants showed an apparently higher proportion of FA 18:1­(9Z) (19.4–38.5%) than the ruminants (18.9–24.0%). The results were consistent with previous studies in humans (26.2–35.5% and 17.0–28.0% for FAs 18:1­(9Z) and 16:0, respectively), dogs (30.0–34.0%, 14.5–17.7%), and pigs (33.2%, 30.8%).^9–11^ FA 18:2­(9Z,12Z) (linoleic acid, LA) was the third most prevalent FA in the TG fractions of human, dog, and pig milk samples, whereas FAs 14:0 and 18:0 were equally abundant in the milk of ruminants. Interestingly, in horse milk the *n*-3 FA 18:3­(9,12,15) (α-Linolenic acid, ALA, 12.7%) was the third most abundant FA. High concentrations of ALA in horse milk (7.1–21.4% of total FAs) have also been documented by others.^13,14,16^ A previous study suggested that infants might benefit from long-chain polyunsaturated FAs (LCPUFAs) when consuming formulas with high ALA content.^34^

Saturated FAs (SFAs) accounted for more than half of the FAs (61.3–73.7%) in ruminants’ milk. In contrast, non-ruminants’ milk contained a higher percentage of unsaturated FAs (UFAs) (51.3–65.4%). According to FA compositions and features of FA classifications (Table S4), such as the groups of monounsaturated FAs (MUFAs) and PUFAs, horse milk was between ruminants and non-ruminants, tending toward the latter. The reason for the features of horse milk fat remains to be explored. However, it has been documented that horse milk proteins are more suitable for infants than cow milk due to their hypo-allergenicity.^35,36^ Dog milk contained more important LCPUFAs and very long-chain PUFAs (VLCFAs) than human milk and other mammalian milk: FA 20:4­(5,8,11,14) (ARA), FA 20:5­(5,8,11,14,17) (EPA), FA 22:5­(7,10,13,16,19) (DPA), and FA 22:6­(4,7,10,13,16,19) (DHA) (1.2, 0.4, 0.5, and 0.4%, respectively). Similarly, ARA and DHA in canine milk (e.g., milk from domestic dogs) have been reviewed in the range of 0.8–1.8% and 0.1–0.4%, respectively.^9^ This might be explained by the puppies’ high requirement for VLCFAs in order to adjust to fast development within a short breastfeeding period of 3–5 weeks.

A key difference in FA composition between foregut fermenters (pseudo-ruminants and ruminants) and non-ruminants lies in the abundance of FAs with acyl chain lengths of less than 10 carbons. These medium- and short-chain FAs (MCFA, SCFA) were found in high abundance in the milk of cows, sheep, and goats; their levels were low in the milk of non-ruminants and camels (pseudo-ruminant). The presence of odd-chain FAs such as FAs 15:0, 17:0, and 17:1 in pseudo-ruminants and ruminants is the result of production by bacteria in the rumen.^12^ Odd-chain SFAs 15:0 and 17:0 are regarded as biomarkers of ruminant-fat intake and alternate endogenous metabolic pathways since they are only derived from dairy fats.^37,38^ In addition, high levels of FAs 15:0 and 17:0 in plasma are negatively correlated with type 2 diabetes mellitus and coronary heart disease.^38–40^ In addition, two trans FAs, 18:1­(9E) and 18:2­(9E,12E), were also found at slightly higher levels in ruminants’ milk, possibly due to the same microbial factor.

Overall, the FA composition of TG fractions in milk from eight different mammalian species varied significantly due to the differences in digestive systems and genetic factors. The FA composition of milk TGs clearly distinguished ruminants from non-ruminants. Ruminants’ milk was richer in SFAs, while non-ruminants have higher UFAs, especially FA 18:1­(9Z). Horse milk showed intermediate characteristics, leaning toward the other non-ruminants in its FA profile.

### 3.2 Triacylglycerol Species Composition

The TG species distribution data presented in Table S5 show that a total of 70 TG species were detected in eight mammalian milk species with ACN ranging from 26–54 and number of DB from 0–7. The number of TG species detected within an individual milk sample varied from 18 in pig milk to 44 in horse milk.

The eight mammalian milk samples were clustered into two groups according to the distribution of the TG species (Figure S2). TGs of milk from the non-ruminant horse as well as three ruminants (cow, goat, and sheep) consisted of over 40 different species, and the other group, including milk of three non-ruminants (dog, pig, and human) and the pseudo-ruminant (camel) contained a remarkably lower number of TG species (18–31). Human milk showed abundant TG species with ACNs of 46, 48, 50, and 52. TG profiles of dog, pig, and camel milk were close to that of human milk, with high abundance in those TGs. Overall, the milk of pig, dog, and camel showed high similarity in the dominating proportion of TG species TG 50:1, TG 50:2, TG 52:2, and TG 52:3. TG 52:2 was the most abundant species in human (23.8 mol %), pig (18.7 mol %), and dog (19.2 mol %) milk, while TG 50:1 (12.8 mol %) was the most dominant species in camel milk. TG 52:2 (8.3–21.0 mol %) has been reported as the most important TG species in human milk.^11,23^ In pig milk, TG 52:3 (18.4 mol %) was also the top species together with TG 52:2. Camel milk showed obviously a different distribution of TG species compared to ruminants due to the lower levels of FAs with a carbon number below 12 (2% compared to over 12% of ruminants).

Horse and the *Bovidae* family (cow, goat, and sheep) milk were clustered into the other group with a high abundance of the TG species with ACNs below 44 (Figure S2). The total number of TG species exceeded 40 in each of these milk samples. However, horse milk showed obvious differences compared to the *Bovidae* family (cow, goat, and sheep), resulting from the even distribution of abundant TG species from ACN 40–52, each with a percentage of less than 7 mol %. This could be explained by the fact that horses belong to a different order (*Perissodactyla*) from the other three animals (*Artiodactyla*). The top three TG species in horse milk were TG 52:4, TG 42:1, and TG 44:1 (about 6 mol % each), followed by TG 46:1, TG 50:2, and TG 52:2 (about 5 mol % each). A previous study reported TG 44:2 (4.5 mol %), TG 44:3 (4.3 mol %), TG 42:2 (4.2 mol %), TG 36:0 (3.5 mol %), and TG 40:1 (3.4 mol %) as the most abundant TG species in horse milk.^23^ Although TG species within ACN 44 were prominent in both studies, our findings showed slightly different proportions: TG 44:2 (2.8 mol %), TG 44:3 (3.2 mol %), TG 42:2 (1.8 mol %), TG 36:0 (1.9 mol %), and TG 40:1 (3.7 mol %). This is because the overall TG profiles in mammalian milk fat are influenced by various factors, such as lactation stage, geographic region, season, diet, and physiological status.^20,21,41,42^ The differences in the abundance of TG species might be attributed to various horse species and diet.^15,43^ Cow, goat, and sheep milks were more similar in the distribution of TG species within even ACN 32–38, which was due to the *Bovidae* family. The most abundant TG species of these three milk species were mainly composed of SFAs such as the TG species TG 32:0, TG 34:0, TG 36:0, and TG 38:0. Similar results were found in a previous study.^11^ Interestingly, low-ACN TG species with only one MUFA were also obvious in cow, goat, sheep, and horse milk, such as TG 38:1, TG 40:1, and TG 42:1. This was attributed to the high proportion of SCFAs and MCFAs (Table S4) of TGs fractions in these milk samples. In addition, it is worth noting that the percentages of FA 18:1 were higher in cow, goat, sheep, and horse milk compared to that in human, pig, and camel milk. This highlights distinct differences in the distribution of FAs within TG molecules across various mammalian milk species.

The distribution of TG species in milk samples revealed two distinct clusters: one with higher TG diversity (horse, cow, goat, and sheep) and another with fewer TG species (human, dog, pig, and camel). Horse milk showed a unique evenly distributed TG profile across a wide range of ACNs, distinguishing it from both ruminants and non-ruminants. The results of TG species distribution are consistent with the findings of the study of Simddy et al.,^20^ where the *Bovidae* family (cow, sheep, and goat) milk were grouped together, clearly separated from horse and camel milk.

### 3.3 Regioisomeric Composition of Triacylglycerols

Table S5 displays the molar percentages of TG regioisomers within each TG species as well as their percentages of total detected TGs in eight different milk species. A total of 976 individual TGs were detected with *sn*-2 FAs identified, covering 362 distinct regioisomer pairs or triplets as well as 5 TGs composed of a single FA: TG 14:0/14:0/14:0, TG 16:0/16:0/16:0, TG 16:1/16:1/16:1, TG 18:1/18:1/18:1, and TG 18:2/18:2/18:2. Additionally, an example chromatogram of the TG species separation of cow milk is provided in Figure S3. Retention time (tR) ranges of TG species are listed in Table S6. Some example MS/MS spectra are shown in Figure S4.

Figure 1 presents the clustering analysis of the milk samples based on the compositions of all detected individual TG regioisomers and single-FA TGs. However, the figure displays only those TGs that were present at concentrations exceeding 1 mol % in at least one milk sample. For clarity and comparative purposes, TGs are arranged in descending order of the relative abundance in human milk. The clustering analysis (Figure 1) revealed two major groups: one comprising human, dog, and pig milk and another encompassing horse milk along with all pseudo-ruminant (camel) and ruminant species (cow, goat, and sheep). The five most abundant TGs in human milk all contained FA 16:0 in *sn*-2 position. This positional specificity is consistent with the known structural features of human milk fat, enhancing fat and calcium absorption in infants. Dog and pig milk also exhibited similar patterns, with their most abundant TGs containing FA 16:0 esterified at the *sn*-2 position, suggesting a potentially comparable nutritional need during early life in these species. The *Bovidae* family (cow, goat, and sheep) showed high similarity in their most abundant TGs. However, camel milk (pseudo-ruminant) was apart from these ruminant species but appeared more closely aligned with the non-ruminant species horse milk. These could be explained by the phylogenetic distance, as well as adaptation to the diet and living environment. Species-specific enzymatic activities, such as acyltransferases in the mammary gland, regulate the positional distribution of FAs on the glycerol backbone during milk fat synthesis.^41,44^

**Figure 1:**
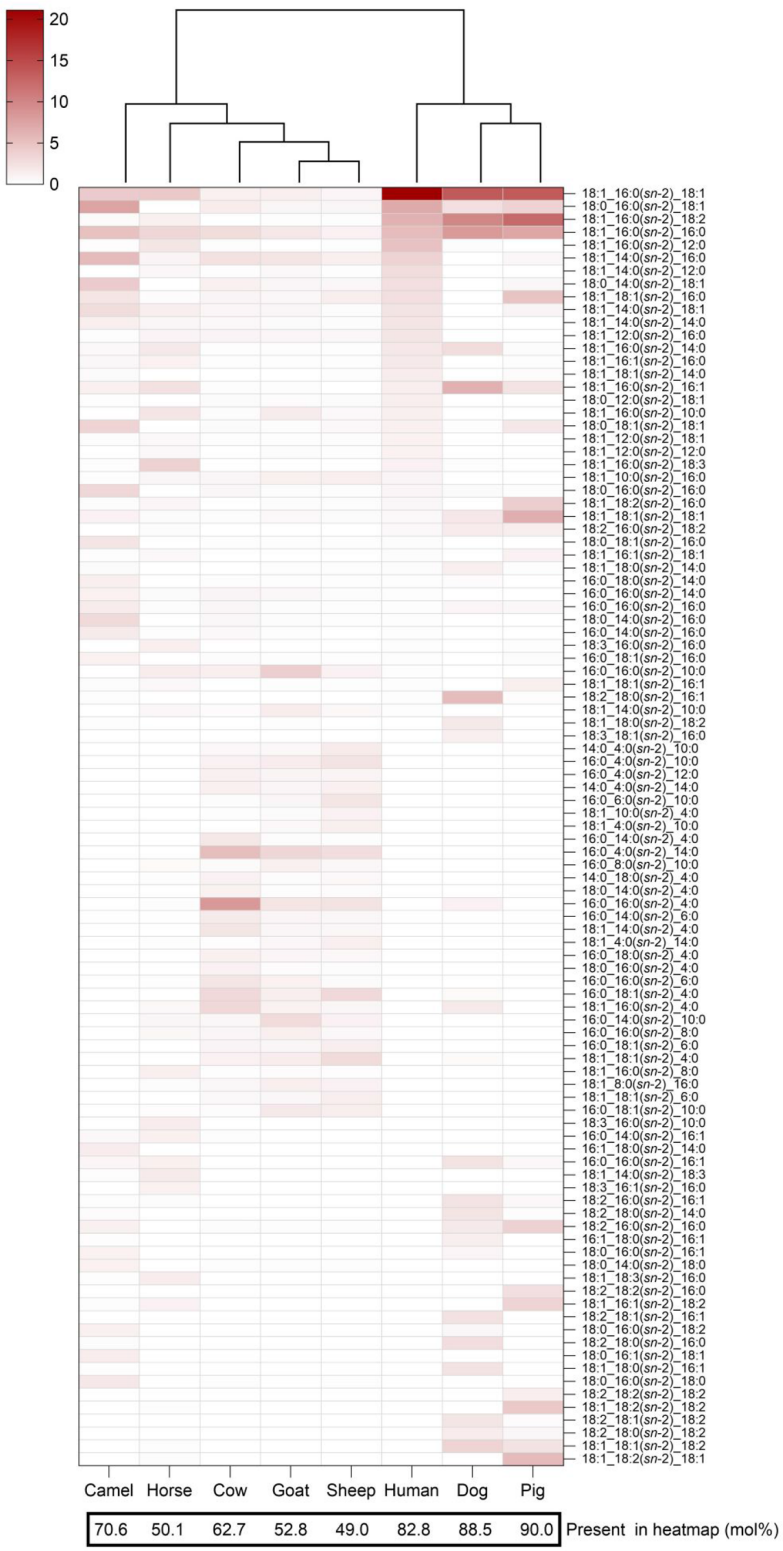
Clustering analysis of mammalian milk based on the proportion of all detected TG molecules (mol %) (Clustering was performed using all detected TG regioisomers and single-FA TGs. The figure displays only those TG regioisomers that are present at minimally 1 mol % in at least one milk sample. The TG regioisomers are shown in descending order of abundance in human milk.).

Table 1 presents the overall TG composition in eight milk samples, categorized based on the number of SFAs and UFAs present in TGs: SSS, SSU/SUS, SUU/USU, and UUU types. Cow, horse and sheep milk had over 40% of TGs with three SFAs (56.9, 52.4, and 44.7 mol %, respectively), corresponding to the abundant SFAs composition (over 68%, Table S4) in each milk. Within the TGs having both SFAs and UFAs, human milk had a typical feature of equal amounts of TGs with one SFA (45.1 mol %) and two SFAs (45.3 mol %) corresponding to previous studies^23,24^ with minor differences (35.3–52.3 and 31.3–32.3, respectively). TGs in non-ruminant horse and ruminants’ milk were mainly composed of two SFAs, while dog and pig milks had more TGs with only one SFA. In addition, horse, dog, and pig milk had higher amounts of TGs with three UFAs (6.5, 11.2, and 27.6 mol %, respectively) than the other milk. Haddad et al.^23^ reported almost equal amounts of TGs in four types (SSS, SSU/SUS, SUU/USU, and UUU types, 22.4–28.5 mol %) in horse milk, which again could be the influence of horse species and diet.

**Table 1:** Overall TG Composition in Milk Samples with Different Number of SFAs (mol %) (S, saturated FA; U, unsaturated FA)

number of SFAs	TG Types	pseudo-ruminant	ruminants			non-ruminants			
		camel	cow	goat	sheep	human	dog	horse	pig
3	SSS	23.9	56.9	52.4	44.7	8.6	3.0	14.4	1.2
	16:0_S(*sn*-2)_S	6.9	14.5	11.8	8.5	2.0	1.3	3.0	0.7
	S_16:0(*sn*-2)_S	3.1	6.5	5.3	3.0	0.9	0.8	2.1	0.3
2	SSU	41.3	27.3	30.6	29.8	42.6	32.7	38.9	18.7
	16:0_S(*sn*-2)_U	5.6	3.1	3.3	2.7	4.3	5.8	4.5	4.5
	S_16:0(*sn*-2)_U	6.4	3.3	2.4	1.2	7.4	8.0	6.7	5.3
2	SUS	8.3	8.9	8.6	13.2	2.8	0.4	4.1	0.9
	16:0_U(*sn*-2)_S	2.8	2.4	1.9	2.9	0.7	0.1	0.8	0.6
1	SUU	12.8	3.6	4.5	9.5	9.3	3.3	13.5	17.0
	16:0_U(*sn*-2)_U	1.5	0.3	0.4	0.6	1.8	0.4	2.0	4.5
1	USU	11.9	2.9	3.3	2.3	35.7	49.3	22.5	34.6
	U_16:0(*sn*-2)_U	2.1	0.5	0.5	0.3	10.2	12.0	4.7	10.6
0	UUU	1.9	0.3	0.7	0.5	1.0	11.2	6.5	27.6

The molar percentages of TG regioisomers containing FA 16:0 are also presented in Table 1. In human milk, *sn*-2 FA 16:0 dominated over *sn*-1,3 FA 16:0 in TGs containing two UFAs (SUU/USU-type, 10.2 vs. 1.8 mol %). Other non-ruminants’ (dog, horse, and pig) as well as pseudo-ruminant’s milk (camel) showed the same tendency. In contrast, the ruminants’ milk (cow, goat, and sheep) contained less than 1 mol % of SUU/USU-type TGs, limiting the comparison of *sn*-positional preferences in these species.

When TGs contain only one UFA (SSU/SUS type), the *sn*-2 FA 16:0 is still noticeable in human milk (7.4 vs. 5.0 mol %). A similar trend was found in the other non-ruminants’ (dog, horse, and pig) milk, though the distribution tended to be more balanced (8.0 vs. 5.9 in dog, 6.7 in vs. 5.3 in horse, 5.3 vs. 5.1 mol % in pig). In contrast, the pseudo-ruminant (camel) and ruminants (cow, goat, and sheep) presented the opposite feature, with FA 16:0 showing either equal distribution or a slight preference for the *sn*-1,3 over the *sn*-2 positions (6.4 vs. 8.4 mol %). These results indicated that milk fat of non-ruminant species may offer comparable nutritional benefits to human milk fat in terms of fat digestibility and calcium absorption.

The regioisomer composition of selected TGs within molecular species contributing ≥ 2 mol % is shown in Figure 2a–d, categorized by the number of SFAs and UFAs.

**Figure 2:**
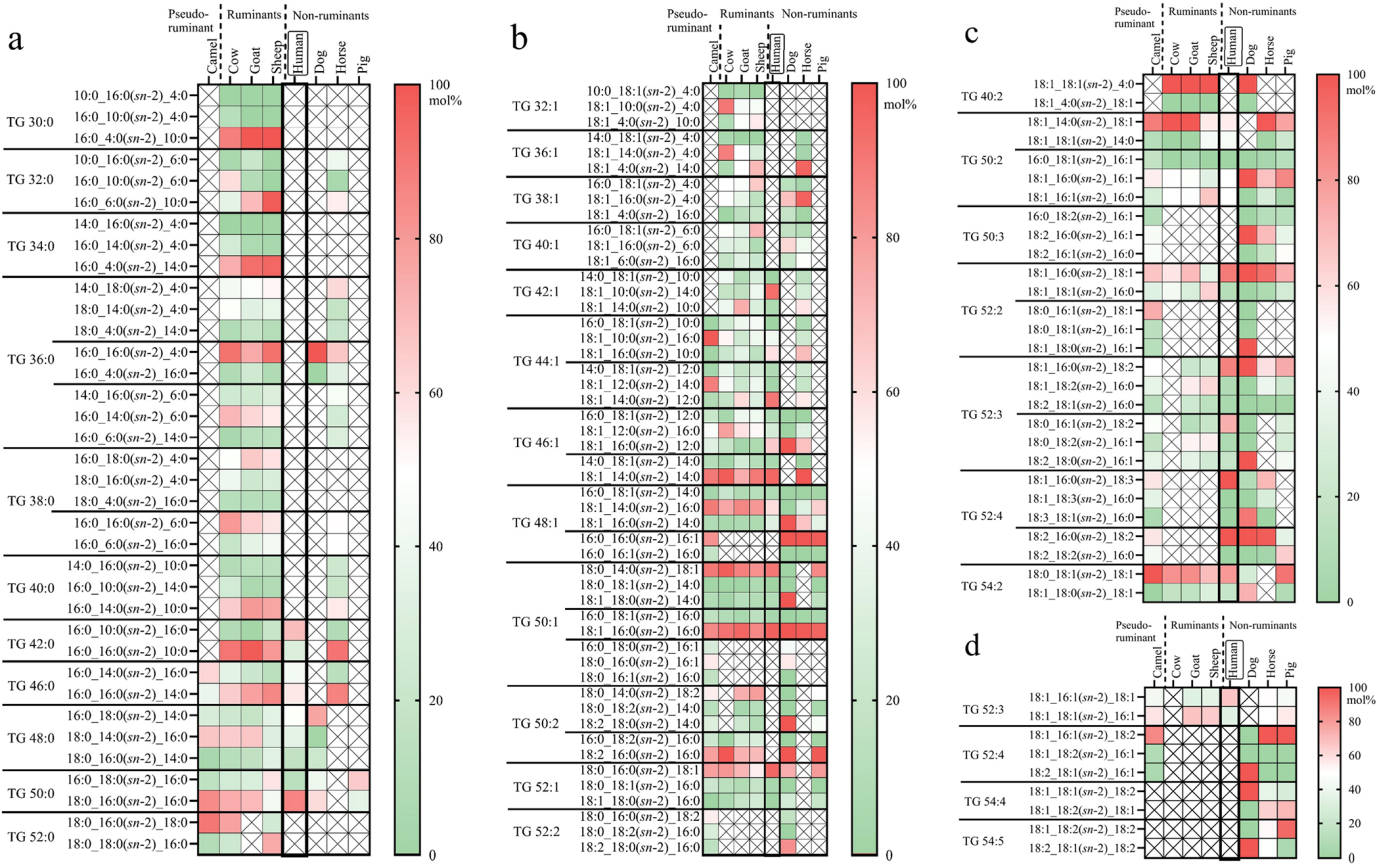
Heatmaps showing regioisomer composition of selected TG regioisomers within TG molecular species contributing ≥ 2 mol % (S, saturated FA; U, unsaturated FA). (a) TG regioisomer composition within each SSS-type regioisomer pair or triplet (mol %); (b) TG regioisomer composition within each SSU/SUS-type regioisomer pair or triplet (mol %); (c) TG regioisomer composition within each SUU/USU-type regioisomer pair or triplet (mol %); and (d) TG regioisomer composition within each UUU-type regioisomer pair or triplet (mol %).

According to Figure 2a, pseudo-ruminant (camel), ruminant (cow, goat, and sheep), and non-ruminant horse milk were rich in SSS-type TGs with ACN below 40, reflecting the higher content of SCFAs and MCFAs. Furthermore, the regioisomeric profile remained consistent across the species within the *Bovidae* family (cow, goat, and sheep) for specific TG molecular species, including TG 16:0_10:0_4:0 (*sn*-2 4:0 dominated), TG 16:0_14:0_4:0 (*sn*-2 4:0 dominated), TG 16:0_16:0_4:0 (*sn*-2 16:0 dominated), and TG 16:0_16:0_10:0 (*sn*-2 16:0 dominated). This suggested conserved biosynthetic features across species within the *Bovidae* family, reflecting the common features of the positional preference of enzymes involved in TG biosynthesis.

In TGs with two SFAs (SSU/SUS types) (Figure 2b), SFAs showed an overall preference for the *sn*-2 position, reflected as the dominance of regioisomers TG 18:1_14:0­(*sn*-2)_14:0, TG 18:1_14:0­(*sn*-2)_16:0, TG 16:0_16:0­(*sn*-2)_16:1, TG 18:0_14:0­(*sn*-2)_18:1 (except dog milk), TG 18:1_16:0­(*sn*-2)_16:0, TG 18:2_16:0­(*sn*-2)_16:0, and TG 18:0_16:0­(*sn*-2)_18:1. However, there was no obvious species-specific *sn*-positional rule in the SSU/SUS TGs. Especially in TG species TG 32:1 to TG 46:1, the abundance of TG regioisomers in each TG molecular species differed among each milk species.

As shown in Figure 2c, TGs with one SFA (SUU/USU types) included two TG regioisomers, TG 18:1_16:0­(*sn*-2)_18:1 (21.3 mol %) and TG 18:1_16:0­(*sn*-2)_18:2 (6.4 mol %), which dominated in human milk. A higher amount of TG 18:1_16:0­(*sn*-2)_18:2 (16.6–19.2%) than TG 18:1_16:0­(*sn*-2)_18:1 (10.1–12.0%) has been reported in Chinese human milk compared to milk of Western mothers.^27^ This could be explained by the higher consumption of soybean and sunflower oil with abundant FA 18:2­(12,15) by Chinese mothers.^45^ In addition, TG 18:1_16:0­(*sn*-2)_18:1 was the dominating regioisomer within the molecular species in human and the other non-ruminants’ (dog, pig, and horse) milk, which indicated the closer similarity in TG molecular structures of these milks to human milk compared to the milk of the ruminant species.

According to Figure 2d, TGs with three UFAs (UUU type) mainly had ACNs of 52 and 54. Non-ruminants’ (dog, horse, and pig) milk presented more abundant TGs with three UFAs, but there were no obvious species-specific rules of FA *sn*-positional preference. Human milk showed a difference in TG molecular species TG 18:1_18:1_16:1. FA 16:1 was preferentially located at the *sn*-2 position, unlike in other milk species.

Minor amounts of TGs containing odd-chain FAs 15:0, 17:0, and 17:1 were found in both pseudo-ruminant’s and ruminants’ milk (Table S5). Odd-carbon-number TG species TG 33:0, TG 35:0, TG 37:0, TG 37:1, TG 39:0, and TG 39:1 in ruminants (cow, goat, and sheep) are composed mainly of SCFAs and MCFAs 4:0, 10:0, and 12:0, as well as some LCFAs. In comparison, TGs in camel milk (pseudo-ruminant) had longer carbon number TG species TG 47:0, TG 47:1, TG 49:0, TG 49:1, TG 49:2, TG 51:1, and TG 51:2 containing odd chain FAs and LCFAs 16:0, 16:1, 18:0, and 18:1. In addition, TGs with two or three odd-chain FAs were found only in camel milk, such as TG 14:0_17:0_17:0, TG 15:0_15:0_18:0, TG 15:0_15:0_17:0, TG 15:0_17:0_17:0, and TG 15:0_17:1_17:1. These different odd-carbon-number TGs in pseudo-ruminant’s and ruminants’ milk indicated the possible influence of different digestion ways on regioisomer compositions. In addition, FA 15:0 showed an obvious preference for the *sn*-2 position in some molecular species, such as the relative abundance of TG 14:0_15:0­(*sn*-2)_10:0, TG 12:0_15:0­(*sn*-2)_12:0, and TG 16:0_15:0­(*sn*-2)_8:0 in in goat milk. TG molecular species containing SCFAs such as TG 6:0_18:0_18:1, TG 6:0_16:0_18:0, TG 4:0_16:0_18:1, TG 6:0_16:0_16:0, and TG 4:0_16:0_18:0 were identified in ruminants’ milk. However, SCFAs did not show obvious *sn*-positional preference.

Branched-chain FAs (*iso* and *anteiso*), such as 13:0, 15:0, 17:0, and 18:0,^46–51^ have also been reported in animal fats. Lísa et al.^48^ detected a wide range of odd-number TGs in eight species of animal fats, including isomer pairs containing branched (br-) and linear FAs (e.g., TG br-18:0_18:1_17:0 vs TG 18:0_18:1_17:0, TG br-18:1_17:0_16:0 vs TG 18:1_17:0_16:0). Unlike linear FAs, branched-chain FAs in mammals are synthesized de novo from branched-chain acyl-CoA derived from branched-chain amino acids such as valine, leucine, and isoleucine.^52^ Unfortunately, methodologies applied in this study could not identify branched-chain FAs.

TG regioisomeric profiles in eight milk species revealed that non-ruminants’ (human, dog, horse, and pig) milk shared high and similar *sn*-2 FA 16:0 characteristics, whereas pseudo-ruminant’s and ruminants’ milk showed distinct structural characteristics linked to species–species adaptations, particularly between the *Bovidae* (cow, goat, and sheep) and *Camelidae* (camel) families. According to clustering analysis, horse milk, apart from the other non-ruminants’ milk, might be attributed to its evenly distributed TG regioisomers composition.

According to the results in Table S4, some of the minor FAs identified in the GC-FID analysis, particularly long-chain FAs with chain lengths of 22 or higher, were not detected in any of the TGs. This could be explained by the targeted nature of our analysis method. After a preliminary MS scan to identify major TGs, the subsequent MS/MS analysis was only performed for those TG species identified in the first step. This likely resulted in the omission of certain low-abundance TGs. However, the analysis results should still cover the large majority of the most relevant and abundant TGs in the samples.

### 3.4 Positional Distribution of Fatty Acids in Triacylglycerols

The *sn*-positional distribution of FAs in detected TG regioisomers varied significantly across eight milk species (Figure 3). Detailed Supporting Information is provided in Table S7.

**Figure 3:**
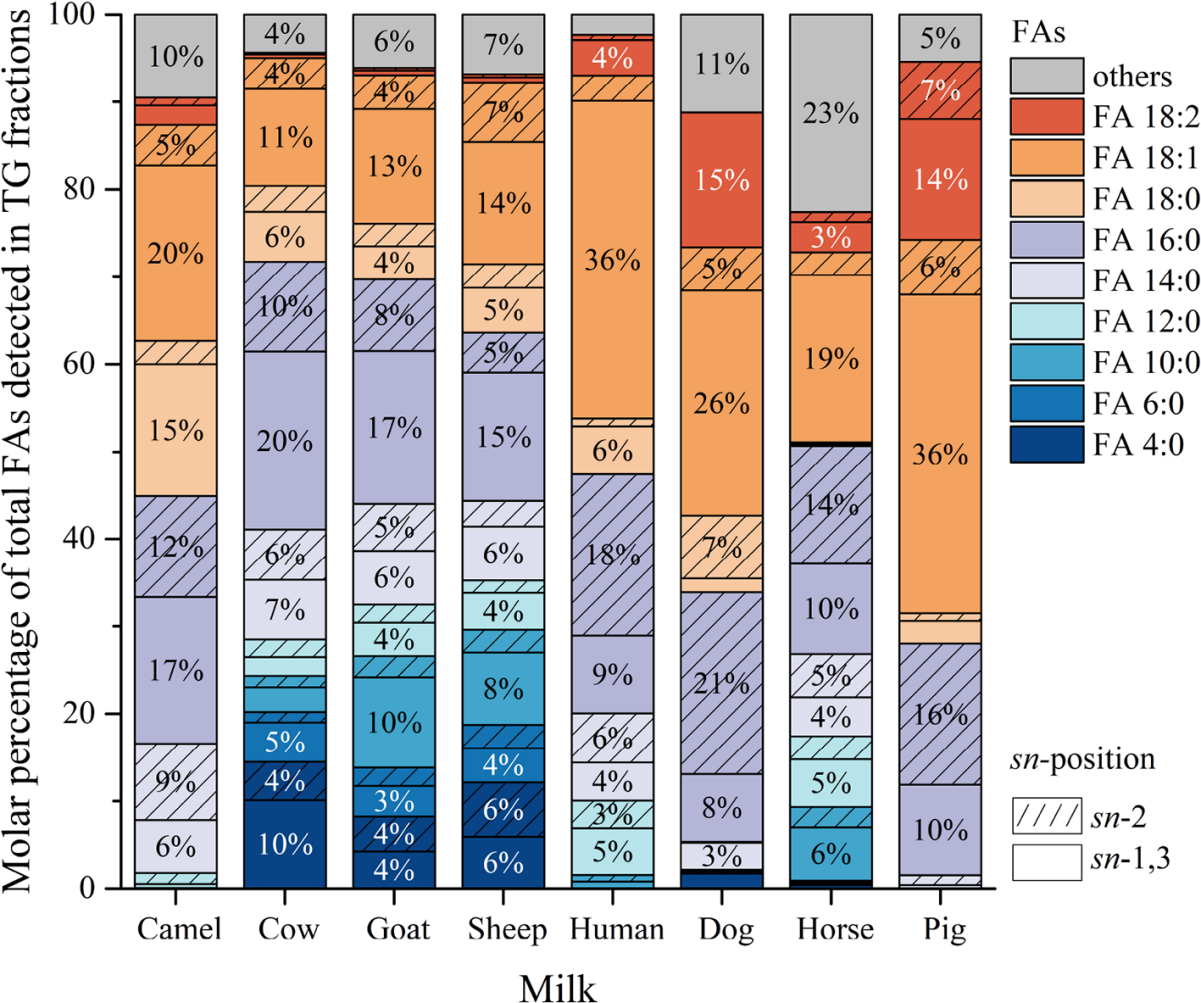
*sn*-positional features of FAs in detected TG regioisomers in eight mammalian milk species (mol %). (Different colors present different FAs, and each FA has two positional options: *sn*-2 (with pattern) and *sn*-1,3 (without pattern).).

The positional distribution of FA 16:0 in TG molecules indicated significant cross-species variations. In agreement with the results of others,^11,27,53^ FA 16:0 had a significant *sn*-2 positional preference in human milk TGs. In the other non-ruminants’ (dog, horse, and pig) milk, FA 16:0 showed similar *sn*-2 preference. However, the distribution of FA 16:0 in camel, cow, goat, and sheep milk favored the *sn*-1,3 positions, being even two or three times more abundant than in the *sn*-2 position in the *Bovidae* family. Commonly used human milk fat substitutes, such as sunflower, palm, and rapeseed oils, had the same positional preference of FA 16:0 for *sn*-1,3 positions,^8^ resulting in a noticeable difference from human milk fat. Despite not being the most abundant FAs in human, dog, or pig milk based on GC results (Table S4), *sn*-2 FA 16:0 was the most abundant *sn*-2 FA in most of the milk samples, except for sheep milk (Figure 3). Similar results of cow and goat milk were reported by Zhang et al.^11^ However, using the lipase-catalyzed acidolysis method, they reported higher *sn*-2 FA 16:0 in cow and goat milk, about 13.6 and 13.0%, compared to 10.3 and 8.2 mol % in the current study, which could be explained by the fact that they did not report the FAs with lower carbon numbers (<10). The proportion of FA *sn*-2 16:0 in human milk was 18.5 mol % in the current study, in line with the range of 15.0–21.3% reported in the study by Zhang et al.^11^

Other abundant SFAs, such as FA 14:0, did not show an obvious preference for any *sn*-positions. Regarding UFAs, FA 18:1 was predominantly esterified at *sn*-1,3 positions in all eight milk samples studied, showing a clear positional preference. While this pattern may seem similar to that observed in many plant oils,^8^ the underlying distribution differs. For example, the distribution of FA 18:1 between *sn*-2 and *sn*-1,3 is often more balanced. Milk fats tend to contain higher amounts of SFAs in the *sn*-2 position, particularly FA 16:0, while UFA 18:1 is more commonly found at the *sn*-1,3 positions. This distribution reflects fundamental differences in the biosynthetic pathways and functional roles of triacylglycerols in mammalian milk compared to plant oils. FA 18:2 showed a similar *sn*-1,3 preference in eight types of milk. In addition, FA 16:1 in dog milk was almost entirely located in the *sn*-1,3 positions. Hence, there might be a rule that UFAs in mammalian milk tend to be esterified at the outer positions of the glycerol backbone, which could be explained by the enzyme specificity in TG biosynthesis in mammals. The same *sn*-1,3 positional preference of UFAs in human and horse milk has also been reported earlier.^23^ In addition, SCFAs and MCFAs with carbon numbers ranging from 4 to 12 were mainly equally esterified at the *sn*-1,3 and *sn*-2 positions, with an *sn*-1,3 preference shown by 6:0 and 10:0 and 12:0.

Positional distribution of FAs in TGs influences their digestion. Related to *sn*-2 FA 16:0, the *in vivo* digestion performances of TG 18:1_16:0­(*sn*-2)_18:1, TG 18:1_16:0­(*sn*-2)_16:0, and TG 16:0_18:1­(*sn*-2)_16:0 have been compared using the *in vitro* gastrointestinal digestion method (INFOGEST).^54^ Individual TG molecules TG 16:0_18:1­(*sn*-2)_16:0 hydrolyzed faster than the other two TGs, while the digestion degree of TG 18:1_16:0­(*sn*-2)_18:1 is the highest among them. Another study demonstrated that feeding rats with TGs having *sn*-3 DHA resulted in higher levels of DHA accumulation in visceral fat.^55^ However, the potential nutritional relevance remains to be investigated pending *in vitro* digestion or *in vivo* feeding studies.

In this study, we analyzed only one milk sample from each mammalian species to detect the interspecies variation in TG regioisomer composition. In the future, multiple samples or pooled samples covering the variation in geography, feeding conditions, lactation stage, and genetic factors should be studied in the subsequent research to provide solid data matrices to enable statistical comparison and better understanding of the biological significances of species-specific features of TG regioisomer profiles.

This study highlights the diversity of TG regioisomer compositions across milk from different mammalian species and underscores the influence of physiological factors (ruminant, pseudo-ruminant, and non-ruminant) and phylogenetic status (families). The similarity of *sn*-2 palmitic acid enrichment in non-ruminants’ (human, dog, horse, and pig) milk implies possible similarities in nutritional properties. Among the foregut fermenters, ruminants’ (cow, goat, and sheep) milk shared highly similar TG regioisomeric profiles, whereas the distinct features of pseudo-ruminant’s (camel) milk further emphasize the complexity of lipid metabolism and fat accumulation in milk.

This research provides new insights into interspecies variation in the lipid metabolism and nutritional properties of milk fats of different mammalian species and especially their potential applications in developing next-generation human milk fat substitutes. Future studies should expand into comprehensive research on the regioisomer profiles of milk glycerophospholipids in different species to provide a holistic view of cross-species variation in the molecular structures and nutritional properties of milk lipids.

## Supplementary Material



## Notes

Clustering analysis of FA composition of mammalian milk TGs; TG regioisomer standards used in this study; calibration results for the LOOCV models and the final model, showing the calculated abundance within a pair for each AAB/ABA type regioisomer standard; calibration results for the LOOCV models and the final model, showing the calculated abundance within a triplet for each ABC type regioisomer standard; FA composition in eight mammalian milk species expressed as relative mass percentage; TG species and regioisomers composition in eight mammalian milk species; clustering analysis of TG species composition of mammalian milk; S3MS scan chromatogram of cow milk TG species; retention times (tR) of TG specie; examples of MS/MS chromatogram and spectra (TGs 52:0, 52:1, 52:2, 52:3, 52:4, and 52:5 in cow and pig milk; TGs 54:2 and 44:0 in cow milk; odd-chain TGs 49:0 and 51:1 in camel milk); *sn*-positional features of FAs in detected TG regioisomers in eight mammalian milk species; and calculation of the number of theoretically possible triacylglycerol regioisomers (PDF)

## Abbreviations

ACN: acyl carbon number
ALA: α-linolenic acid
ANOVA: one-way analysis of variance
ARA: arachidonic acid
DB: double bond
DG: diacylglycerol
DHA: docosahexaenoic acid
DPA: docosapentaenoic acid
EPA: eicosapentaenoic acid
ESI: electrospray ionization
FA: fatty acid
FAME: fatty acid methyl ester
FID: flame ionization detector
GC: gas chromatography
MS/MS: tandem mass spectrometry
LA: linoleic acid
LCFA: long-chain fatty acid
LCPUFA: long-chain polyunsaturated fatty acid
LOOCV: leave-one-out cross-validation
MCFA: medium-chain fatty acid
MUFA: monounsaturated fatty acid
PUFA: polyunsaturated fatty acid
SFA: saturated fatty acid
SCFA: short-chain fatty acid
*sn*: stereospecific numbering
SPE: solid-phase extraction
TG: triacylglycerol
tR: retention time
UFA: unsaturated fatty acid
UHPLC: ultrahigh performance liquid chromatography
VLCFA: very long-chain fatty acid

## References

[1] Guo J., Duan H., Zheng X., Wang D., Zhou Y., Zhou S., Yan W. (2025). Comparative Assessment of Nutritional
Value in Milk through Fatty Acid from Various Sources: A Review. J. Future Foods.

[2] German J. B., Dillard C. J. (2006). Composition, Structure and Absorption
of Milk Lipids:
A Source of Energy, Fat-Soluble Nutrients and Bioactive Molecules. Crit Rev. Food Sci. Nutr..

[3] Ten-Doménech I., Beltrán-Iturat E., Herrero-Martínez J. M., Sancho-Llopis J. V., Simó-Alfonso E.
F. (2015). Triacylglycerol Analysis
in Human Milk and Other Mammalian Species: Small-Scale Sample Preparation,
Characterization, and Statistical Classification Using HPLC-ELSD Profiles. J. Agric. Food Chem..

[4] Innis S. M. (2011). Dietary
Triacylglycerol Structure and Its Role in Infant Nutrition. Adv. Nutr..

[5] Han X., Ye H. (2021). Overview of Lipidomic Analysis of Triglyceride Molecular Species
in Biological Lipid Extracts. J. Agric. Food
Chem..

[6] Ghide M. K., Yan Y. (2021). 1,3-Dioleoyl-2-Palmitoyl Glycerol (OPO)—Enzymatic Synthesis
and Use as an Important Supplement in Infant Formulas. J. Food Biochem..

[7] Cao H., Liu Q., Liu Y., Zhao J., Qiao W., Wang Y., Liu Y., Chen L. (2024). Progress in Triacylglycerol Isomer Detection in Milk
Lipids. Food Chem.: X.

[8] Zhao Q., Kalpio M., Fabritius M., Zhang Y., Yang B. (2025). Analysis of
Triacylglycerol Regioisomers in Plant Oils Using Direct Inlet Negative
Ion Chemical Ionization Tandem Mass Spectrometry. Food Res. Int..

[9] Zhang M., Sun X., Cheng J., Guo M. (2022). Analysis and Comparison of Nutrition
Profiles of Canine Milk with Bovine and Caprine Milk. Foods.

[10] Mountzouris K. C., Fegeros K., Papadopoulos G. (1999). Utilization
of Fats Based on the
Composition of Sow Milk Fat in the Diet of Weanling Pigs. Animal feed science and technology.

[11] Zhang X., Wei W., Tao G., Jin Q., Wang X. (2021). Identification and
Quantification of Triacylglycerols Using Ultraperformance Supercritical
Fluid Chromatography and Quadrupole Time-of-Flight Mass Spectrometry:
Comparison of Human Milk, Infant Formula, Other Mammalian Milk, and
Plant Oil. J. Agric. Food Chem..

[12] Bas P., Archimède H., Rouzeau A., Sauvant D. (2003). Fatty Acid Composition
of Mixed-Rumen Bacteria: Effect of Concentration and Type of Forage. J. Dairy Sci..

[13] Curadi M. C., Leotta R., Contarini G., Orlandi M. (2012). Milk fatty acids from
different horse breeds compared with cow, goat and human milk. Maced J. Anim Sci..

[14] Barłowska J., Polak G., Janczarek I., Próchniak T. (2023). Chemical Composition,
Whey Protein Profile, and Fatty Acid Profile of Milk from Sokólski
Horses in Relation to Polish Halfbred Horses. Ann. Anim. Sci..

[15] Barłowska J., Polak G., Janczarek I., Tkaczyk E. (2023). The Influence of Selected
Factors on the Nutritional Value of the Milk of Cold-Blooded Mares:
The Example of the Sokólski Breed. Animals.

[16] Pikul J., Wójtowski J., Danków R., Kuczyńska B., Łojek J. (2008). Fat Content and Fatty Acids Profile
of Colostrum and
Milk of Primitive Konik Horses (Equus Caballus Gmelini Ant.) during
Six Months of Lactation. J. Dairy Res..

[17] Wang D. H., Wang Z., Chen R., Brenna J. T. (2020). Characterization
and Semiquantitative Analysis of Novel Ultratrace C10–24Monounsaturated
Fatty Acid in Bovine Milkfat by Solvent-Mediated Covalent Adduct Chemical
Ionization (CACI) MS/MS. J. Agric. Food Chem..

[18] Chen B., Zhu H., Zhang Y., Wang X., Zhang W., Wang Y., Pang X., Zhang S., Lv J. (2024). Comparison of Species
and Lactation of Different Mammalian Milk: The Unique Composition
and Stereospecificity of Fatty Acids of Mare Milk. Int. Dairy J..

[19] Mitina A., Mazin P., Vanyushkina A., Anikanov N., Mair W., Guo S., Khaitovich P. (2020). Lipidome Analysis
of Milk Composition in Humans, Monkeys,
Bovids, and Pigs. BMC Evol Biol..

[20] Smiddy M. A., Huppertz T., van Ruth S. M. (2012). Triacylglycerol
and Melting Profiles
of Milk Fat from Several Species. Int. Dairy
J..

[21] Tu A., Ma Q., Bai H., Du Z. (2017). A Comparative Study of Triacylglycerol
Composition in Chinese Human Milk within Different Lactation Stages
and Imported Infant Formula by SFC Coupled with Q-TOF-MS. Food Chem..

[22] Wang X., Zhu H., Zhang W., Zhang Y., Zhao P., Zhang S., Pang X., Vervoort J., Lu J., Lv J. (2022). Triglyceride
and Fatty Acid Composition of Ruminants Milk, Human Milk, and Infant
Formulae. J. Food Compos. Anal..

[23] Haddad I., Mozzon M., Strabbioli R., Frega N. G. (2012). A Comparative Study
of the Composition of Triacylglycerol Molecular Species in Equine
and Human Milks. Dairy Sci. Technol..

[24] Yu J., Wang S., Wang F., Wang L., Wei W., Wang Q., Wang X. (2024). Triacylglycerols
in Human Milk and
Their Association with Edible Oils in Maternal Diet: A Study of Five
Regions in China. J. Food Compos. Anal..

[25] Zhang X., Wei W., Tao G., Jin Q., Wang X. (2022). Triacylglycerol Regioisomers
Containing Palmitic Acid Analyzed by Ultra-Performance Supercritical
Fluid Chromatography and Quadrupole Time-of-Flight Mass Spectrometry:
Comparison of Standard Curve Calibration and Calculation Equation. Food Chem..

[26] Liu Z., Rochfort S. (2021). Bovine Milk Triacylglycerol
Regioisomer Ratio Shows
Remarkable Inter-Breed and Inter-Cow Variation. Molecules.

[27] Chen Y. J., Zhou X. H., Han B., Yu Z., Yi H. X., Jiang S. L., Li Y. Y., Pan J. C., Zhang L. W. (2020). Regioisomeric
and Enantiomeric Analysis of Primary Triglycerides in Human Milk by
Silver Ion and Chiral HPLC Atmospheric Pressure Chemical Ionization-MS. J. Dairy Sci..

[28] Zou X., Huang J., Jin Q., Guo Z., Liu Y., Cheong L., Xu X., Wang X. (2013). Lipid Composition Analysis
of Milk Fats from Different Mammalian Species: Potential for Use as
Human Milk Fat Substitutes. J. Agric. Food Chem..

[29] Sazzad M. A. Al., Fabritius M., Boström P., Tarvainen M., Kalpio M., Linderborg K. M., Kallio H., Yang B. (2022). A Novel UHPLC-ESI-MS/MS
Method and Automatic Calculation Software for Regiospecific Analysis
of Triacylglycerols in Natural Fats and Oils. Anal. Chim. Acta.

[30] Al
Sazzad M. A., Fabritius M., Boström P., Yang B. (2024). Advanced Tandem Mass Spectrometric Analysis of Complex Mixtures of
Triacylglycerol Regioisomers: A Case Study of Bovine Milk Fat. J. Agric. Food Chem..

[31] Liebisch G., Fahy E., Aoki J., Dennis E. A., Durand T., Ejsing C. S., Fedorova M., Feussner I., Griffiths W. J., Köfeler H., Merrill A. H., Murphy R. C., O’Donnell V. B., Oskolkova O., Subramaniam S., Wakelam M. J. O., Spener F. (2020). Update on
LIPID MAPS Classification, Nomenclature, and Shorthand Notation for
MS-Derived Lipid Structures. J. Lipid Res..

[32] Folch J., Lees M., Sloane G. H. (1957). A simple
method for the isolation
and purification of total lipides from animal tissues. J. Biol. Chem..

[33] Signorelli F., Contarini G., Annicchiarico G., Napolitano F., Orrù L., Catillo G., Haenlein G. F. W., Moioli B. (2008). Breed Differences
in Sheep Milk Fatty Acid Profiles: Opportunities for Sustainable Use
of Animal Genetic Resources. Small Ruminant
Research.

[34] Clark K. J., Makrides M., Neumann M. A., Gibson R. A., Gibson R. (1992). Determination
of the optimal ratio of linoleic acid to α-linolenic acid in
infant formulas. J. Pediatr..

[35] Businco L., Giampietro P. G., Lucenti P., Lucaroni F., Pini C., Di Felice G., Iacovacci P., Curadi C., Orlandi M. (2000). Allergenicity
of Mare’s Milk in Children with Cow’s Milk Allergy. J. Allergy Clin. Immunol..

[36] Curadi M. C., Giampietro P. G., Lucenti P., Orlandi M. (2001). Use of Mare Milk in
Pediatric Allergology. Proc. ASPA XIV Congress,
Italy.

[37] Wolk, A. ; Bengt, V. ; Hãkan, L. ; Peter, B. Evaluation of a Biological Marker of Dairy Fat Intake. 1998, 68 (2), 291–295. 10.1093/ajcn/68.2.291. 9701185

[38] Jenkins B., West J. A., Koulman A. (2015). A Review of Odd-Chain
Fatty Acid
Metabolism and the Role of Pentadecanoic Acid (C15:0) and Heptadecanoic
Acid (C17:0) in Health and Disease. Molecules.

[39] Forouhi N. G., Koulman A., Sharp S. J., Imamura F., Kröger J., Schulze M. B., Crowe F. L., Huerta J. M., Guevara M., Beulens J. W. J., van
Woudenbergh G. J., Wang L., Summerhill K., Griffin J. L., Feskens E. J. M., Amiano P., Boeing H., Clavel-Chapelon F., Dartois L., Fagherazzi G., Franks P. W., Gonzalez C., Jakobsen M. U., Kaaks R., Key T. J., Khaw K. T., Kühn T., Mattiello A., Nilsson P. M., Overvad K., Pala V., Palli D., Quirós J. R., Rolandsson O., Roswall N., Sacerdote C., Sánchez M. J., Slimani N., Spijkerman A. M. W., Tjonneland A., Tormo M. J., Tumino R., van der A D. L., van der Schouw Y. T., Langenberg C., Riboli E., Wareham N. J. (2014). Differences
in the Prospective Association between Individual Plasma Phospholipid
Saturated Fatty Acids and Incident Type 2 Diabetes: The EPIC-InterAct
Case-Cohort Study. Lancet Diabetes Endocrinol.

[40] Furse S., Fernandez-Twinn D. S., Beeson J. H., Chiarugi D., Ozanne S. E., Koulman A. A. (2022). A mouse
model of gestational diabetes shows dysregulated
lipid metabolism post-weaning, after return to euglycaemia. Nutr Diabetes.

[41] Rudolph M. C., Neville M. C., Anderson S. M. (2007). Lipid Synthesis
in Lactation: Diet
and the Fatty Acid Switch. J. Mammary Gland
Biol. Neoplasia.

[42] Linderborg K. M., Kalpio M., Mäkelä J., Niinikoski H., Kallio H. P., Lagström H. (2014). Tandem Mass
Spectrometric Analysis
of Human Milk Triacylglycerols from Normal Weight and Overweight Mothers
on Different Diets. Food Chem..

[43] Hoffman R. M., Kronfeld D. S., Herbein J. H., Swecker W. S., Cooper W. L., Harris P. A. (1998). Dietary Carbohydrates
and Fat Influence Milk Composition
and Fatty Acid Profile of Mare’s Milk. Journal of nutrition.

[44] Innis S. M. (2007). Human Milk:
Maternal Dietary Lipids and Infant Development. Proceedings of the Nutrition Society.

[45] Orsavova J., Misurcova L., Ambrozova J., Vicha R., Mlcek J. (2015). Fatty Acids
Composition of Vegetable Oils and Its Contribution to Dietary Energy
Intake and Dependence of Cardiovascular Mortality on Dietary Intake
of Fatty Acids. Int. J. Mol. Sci..

[46] Teng F., Wang P., Yang L., Ma Y., Day L. (2017). Quantification
of Fatty Acids in Human, Cow, Buffalo, Goat, Yak, and Camel Milk Using
an Improved One-Step GC-FID Method. Food Anal.
Methods.

[47] Vlaeminck B., Fievez V., Tamminga, Dewhurst R. J., Van Vuuren A., De Brabander D., Demeyer D. (2006). Milk Odd- and Branched-Chain Fatty
Acids in Relation to the Rumen Fermentation Pattern. J. Dairy Sci..

[48] Lísa M., Netušilová K., Franěk L., Dvořáková H., Vrkoslav V., Holčapek M. (2011). Characterization
of Fatty Acid and Triacylglycerol Composition in Animal Fats Using
Silver-Ion and Non-Aqueous Reversed-Phase High-Performance Liquid
Chromatography/Mass Spectrometry and Gas Chromatography/Flame Ionization
Detection. J. Chromatogr A.

[49] Egge H., Murawski U., Ryhage R., György P., Chatranon W., Zilliken F. (1972). Minor constitutents
of human milk
IV: Analysis of the branched chain fatty acids. Chem. Phys. Lipids.

[50] Massart-Leën A. M., De Pooter H., Decloedt M., Schamp N. (1981). Composition and variability
of the branched-chain fatty acid fraction in the milk of goats and
cows. Lipids.

[51] Wang F., Chen M., Luo R., Huang G., Wu X., Zheng N., Zhang Y., Wang J. (2022). Fatty Acid Profiles
of Milk from Holstein Cows, Jersey Cows, Buffalos, Yaks, Humans, Goats,
Camels, and Donkeys Based on Gas Chromatography–Mass Spectrometry. J. Dairy Sci..

[52] Vlaeminck B., Fievez V., Cabrita A. R. J., Fonseca A. J. M., Dewhurst R. J. (2006). Factors
Affecting Odd- and Branched-Chain Fatty Acids in Milk: A Review. Anim. Feed Sci. Technol..

[53] Fabritius M., Linderborg K. M., Tarvainen M., Kalpio M., Zhang Y., Yang B. (2020). Direct Inlet
Negative Ion Chemical Ionization Tandem Mass Spectrometric
Analysis of Triacylglycerol Regioisomers in Human Milk and Infant
Formulas. Food Chem..

[54] Chang H. J., Lee J. H. (2021). Regiospecific Positioning of Palmitic Acid in Triacylglycerol
Structure of Enzymatically Modified Lipids Affects Physicochemical
and in Vitro Digestion Properties. Molecules.

[55] Zhang Y., Kalpio M., Tao L., Haraldsson G. G., Gudmundsson H. G., Fang X., Linderborg K. M., Zhang Y., Yang B. (2023). Metabolic Fate of DHA from Regio-
and Stereospecific Positions of Triacylglycerols in a Long-Term Feeding
Trial in Rats. Food Res. Int..

