# Supplementary material for: Tandem Mass Spectrometric Analysis and Cross-Species Comparison of Triacylglycerol Regioisomers in Mammalian Milk
Source: J Agric Food Chem. 2026 Jan 5;74(1):1410–21. doi: 10.1021/acs.jafc.5c08520 (PMC12814545; doi:10.1021/acs.jafc.5c08520)
Supplement: Supplementary file 1 [file jf5c08520_si_001.pdf]

Supporting Information

Tandem mass spectrometric analysis and cross-species comparison of triacylglycerol regioisomers in mammalian milk

Qizhu Zhao [1], *Mikael Fabritius* [1], *Marika Kalpio, Md Abdullah Al Sazzad, Baoru Yang**

Food Sciences Unit, Department of Life Technologies, Faculty of Technology, University of Turku, FI-20014 Turku, Finland

[1] These authors contributed equally.

* Corresponding author

Professor Baoru Yang

Food Sciences Unit, Department of Life Technologies, Faculty of Technology, University of Turku, FI-20014 Turku, Finland

E-mail: baoru.yang@utu.fi

Phone: +358 29 450 2917

Contents

Figure S1 Clustering analysis of FA composition of mammalian milk TGs (%)........S2

Table S1 TG regioisomer standards used in this study........S3

Table S2 Calibration results for the LOOCV models and the final model, showing the calculated abundance within a pair for each AAB/ABA type regioisomer standard........S5

Table S3 Calibration results for the LOOCV models and the final model, showing the calculated abundance within a triplet for each ABC type regioisomer standard........S7

Table S4 FA composition in eight mammalian milk species expressed as relative mass percentage (%)........S8

Table S5 TG species and regioisomers composition in eight mammalian milk species (mol%)........S9

Figure S2 Clustering analysis of TG species composition of mammalian milk (mol%).........S28

Figure S3 MS scan chromatogram of cow milk TG species........S29

Table S6 Retention times (tR) of TG species.........S30

Figure S4 Examples of MS/MS chromatogram and spectra........S31

Table S7 The *sn*-positional features of FAs in detected TG regioisomers in eight mammalian milk species (mol%)........S33

Formula S1 Calculation of the number of theoretically possible triacylglycerol regioisomers........S34

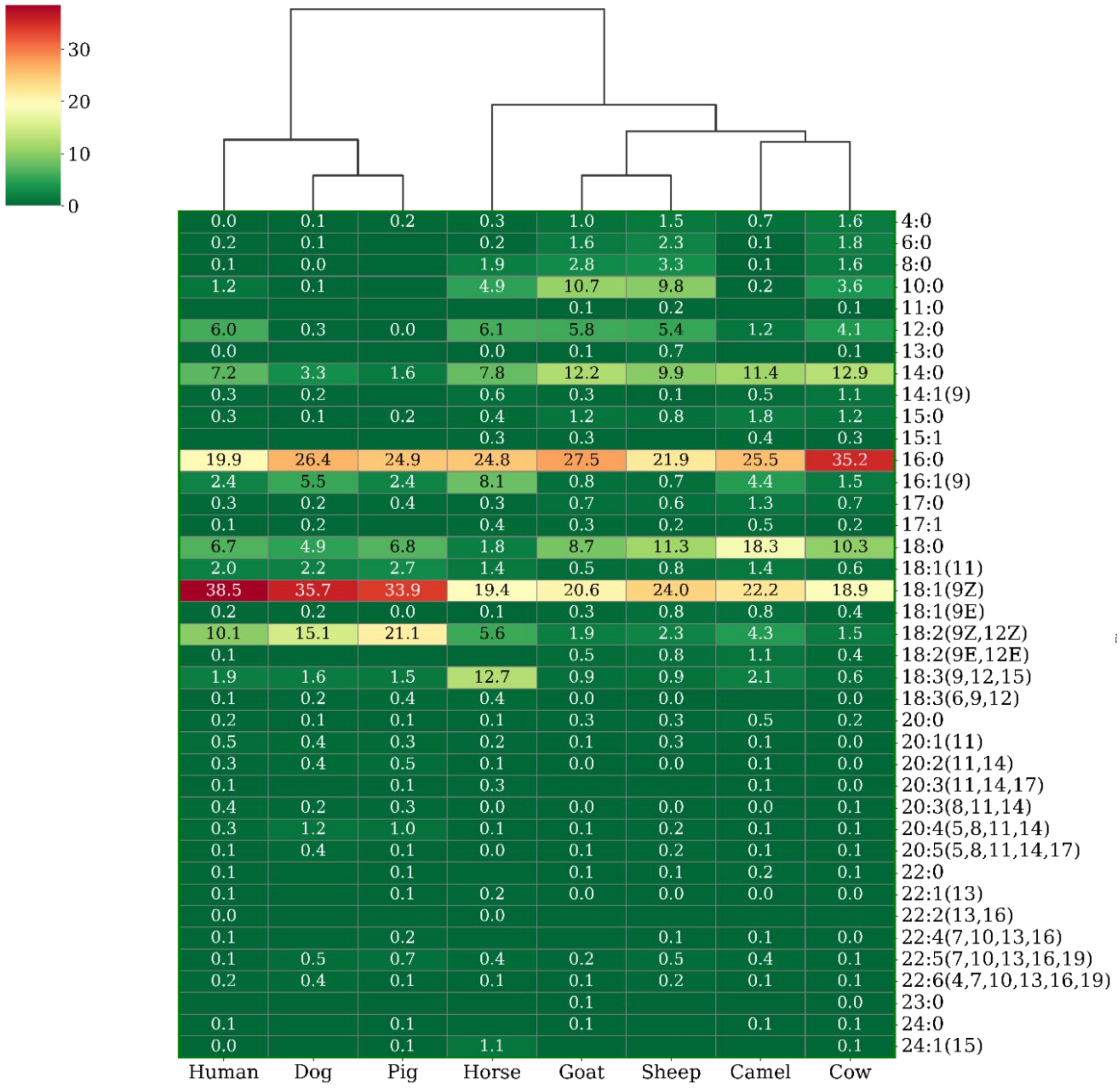

Figure S1 Clustering analysis of FA composition of mammalian milk TGs (%).

Table S1 TG regioisomer standards used in this study.
(All standards were purchased from Larodan, Sweden.)

Systematic name	Shorthand name
1(3)-butyryl-2-butyryl-3(1)-palmitoyl-glycerol	TG 4:0_4:0 (*sn*-2)_16:0
1(3)-butyryl-2-palmitoyl-3(1)-butyryl-glycerol	TG 4:0_16:0 (*sn*-2)_4:0
1(3)-butyryl-2-butyryl-3(1)-stearoyl-glycerol	TG 4:0_4:0 (*sn*-2)_18:0
1(3)-butyryl-2-stearoyl-3(1)-butyryl-glycerol	TG 4:0_18:0 (*sn*-2)_4:0
1(3)-caproyl-2-caproyl-3(1)-myristoyl-glycerol	TG 10:0_10:0 (*sn*-2)_14:0
1(3)-caproyl-2-myristoyl-3(1)-caproyl-glycerol	TG 10:0_14:0 (*sn*-2)_10:0
1(3)-caproyl-2-caproyl-3(1)-palmitoyl-glycerol	TG 10:0_10:0 (*sn*-2)_16:0
1(3)-caproyl-2-palmitoyl-3(1)-caproyl-glycerol	TG 10:0_16:0 (*sn*-2)_10:0
1(3)-caproyl-2-caproyl-3(1)-stearoyl-glycerol	TG 10:0_10:0 (*sn*-2)_18:0
1(3)-caproyl-2-stearoyl-3(1)-caproyl-glycerol	TG 10:0_18:0 (*sn*-2)_10:0
1(3)-caproyl-2-dicaproyl-3(1)-oleoyl-glycerol	TG 10:0_10:0 (*sn*-2)_18:1
1(3)-caproyl-2-oleoyl-3(1)-caproyl-glycerol	TG 10:0_18:1 (*sn*-2)_10:0
1(3)-myristoyl-2-myristoyl-3(1)-oleoyl-glycerol	TG 14:0_14:0 (*sn*-2)_18:1
1(3)-myristoyl-2-oleoyl-3(1)-myristoyl-glycerol	TG 14:0_18:1 (*sn*-2)_14:0
1(3)-palmitoyl-2-palmitoyl-3(1)-butyryl-glycerol	TG 16:0_16:0 (*sn*-2)_4:0
1(3)-palmitoyl-2-butyryl-3(1)-palmitoyl-glycerol	TG 16:0_4:0 (*sn*-2)_16:0
1(3)palmitoyl-2-palmitoyl-3(1)-capryloyl-glycerol	TG 16:0_16:0 (*sn*-2)_8:0
1(3)-palmitoyl-2-capryloyl-3(1)-palmitoyl-glycerol	TG 16:0_8:0 (*sn*-2)_16:0
1(3)-palmitoyl-2-palmitoyl-3(1)-myristoyl-glycerol	TG 16:0_16:0 (*sn*-2)_14:0
1(3)-palmitoyl-2-myristoyl-3(1)palmitoyl-glycerol	TG 16:0_14:0 (*sn*-2)_16:0
1(3)-palmitoyl-2-palmitoyl-3(1)-stearoyl-glycerol	TG 16:0_16:0 (*sn*-2)_18:0
1(3)-palmitoyl-2-stearoyl-3(1)-palmitoyl-glycerol	TG 16:0_18:0 (*sn*-2)_16:0
1(3)-palmitoyl-2-palmitoyl-3(1)-oleoyl-glycerol	TG 16:0_16:0 (*sn*-2)_18:1
1(3)-palmitoyl-2-oleoyl-3(1)-palmitoyl-glycerol	TG 16:0_18:1 (*sn*-2)_16:0
1(3)-palmitoyl-2-palmitoyl-3(1)-linoleoyl-glycerol	TG 16:0_16:0 (*sn*-2)_18:2
1(3)-palmitoyl-2-linoleoyl-3(1)-palmitoyl-glycerol	TG 16:0_18:2 (*sn*-2)_16:0
1(3)-stearoyl-2-stearoyl-3(1)-palmitoyl-glycerol	TG 18:0_18:0 (*sn*-2)_16:0
1(3)-stearoyl-2-palmitoyl-3(1)-stearoyl-glycerol	TG 18:0_16:0 (*sn*-2)_18:0
1(3)-stearoyl-2-stearoyl-3(1)-oleoyl-glycerol	TG 18:0_18:0 (*sn*-2)_18:1
1(3)-stearoyl-2-oleoyl-3(1)-stearoyl-glycerol	TG 18:0_18:1 (*sn*-2)_18:0
1(3) stearoyl-2-stearoyl-3(1)-linoleoyl-glycerol	TG 18:0_18:0 (*sn*-2)_18:2
1(3)-stearoyl-2-linoleoyl-3(1)-stearoyl-glycerol	TG 18:0_18:2 (*sn*-2)_18:0
1(3)-oleoyl-2-oleoyl-3(1)-butyryl-glycerol	TG 18:1_18:1 (*sn*-2)_4:0
1(3)-oleoyl-2-butyryl-3(1)-oleoyl-glycerol	TG 18:1_4:0 (*sn*-2)_18:1
1(3)-oleoyl-2-oleoyl-3(1)-lauroyl-glycerol	TG 18:1_18:1 (*sn*-2)_12:0
1(3)-oleoyl-2-lauroyl-3(1)-oleoyl-glycerol	TG 18:1_12:0 (*sn*-2)_18:1
1(3)-oleoyl-2-oleoyl-3(1)-myristoyl-glycerol	TG 18:1_18:1 (*sn*-2)_14:0
1(3)-oleoyl-2-myristoyl-3(1)-oleoyl-glycerol	TG 18:1_14:0 (*sn*-2)_18:1
1(3)-oleoyl-2-oleoyl-3(1)-palmitoyl-glycerol	TG 18:1_18:1 (*sn*-2)_16:0
1(3)-oleoyl-2-palmitoyl-3(1)-oleoyl-glycerol	TG 18:1_16:0 (*sn*-2)_18:1
1(3)-oleoyl-2-oleoyl-3(1)-stearoyl-glycerol	TG 18:1_18:1 (*sn*-2)_18:0
1(3)-oleoyl-2-stearoyl-3(1)-oleoyl-glycerol	TG 18:1_18:0 (*sn*-2)_18:1
1(3)-oleoyl-2-oleoyl-3(1)-linoleoyl-glycerol	TG 18:1_18:1 (*sn*-2)_18:2
1(3)-oleoyl-2-linoleoyl-3(1)-oleoyl-glycerol	TG 18:1_18:2 (*sn*-2)_18:1

1(3)-oleoyl-2-oleoyl-3(1)-linolenoyl-glycerol	TG 18:1_18:1 (*sn*-2)_18:3
1(3)-oleoyl-2-linolenoyl-3(1)-oleoyl-glycerol	TG 18:1_18:3 (*sn*-2)_18:1
1(3)-linoleoyl-2- linoleoyl-3(1)-palmitoyl-glycerol	TG 18:2_18:2 (*sn*-2)_16:0
1(3)-linoleoyl-2-palmitoyl-3(1)-linoleoyl-glycerol	TG 18:2_16:0 (*sn*-2)_18:2
1(3)-linoleoyl-2-linoleoyl-3(1)-oleoyl-glycerol	TG 18:2_18:2 (*sn*-2)_18:1
1(3)-linoleoyl-2-oleoyl-3(1)linoleoyl-glycerol	TG 18:2_18:1 (*sn*-2)_18:2
1(3)-linoleoyl-2- linoleoyl -3(1)-linolenoyl-glycerol	TG 18:2_18:2 (*sn*-2)_18:3
1(3)-linoleoyl-2-linolenoyl-3(1)-linoleoyl-glycerol	TG 18:2_18:3 (*sn*-2)_18:2
1(3)-butyryl-2-palmitoyl-3(1)-oleoyl-glycerol	TG 4:0_16:0 (*sn*-2)_18:1
1(3)-butyryl-2-oleoyl-3(1)-palmitoyl-glycerol	TG 4:0_18:1 (*sn*-2)_16:0
1(3)-oleoyl-2-butyryl-3(1)-palmitoyl-glycerol	TG 18:1_4:0 (*sn*-2)_16:0
1(3)-hexanoyl-2-palmitoyl-3(1)-oleoyl-glycerol	TG 6:0_16:0 (*sn*-2)_18:1
1(3)-hexanoyl-2-oleoyl-3(1)-palmitoyl-glycerol	TG 6:0_18:1 (*sn*-2)_16:0
1(3)-oleoyl-2-hexanoyl-3(1)-palmitoyl-glycerol	TG 18:1_6:0 (*sn*-2)_16:0
1(3)-palmitoyl-2-oleoyl-3(1)-lauroyl-glycerol	TG 16:0_18:1 (*sn*-2)_12:0
1(3)-oleoyl-2-lauroyl-3(1)-palmitoyl-glycerol	TG 18:1_12:0 (*sn*-2)_16:0
1(3)-oleoyl-2-palmitoyl-3(1)-lauroyl-glycerol	TG 18:1_16:0 (*sn*-2)_12:0
1(3)-linoleoyl-2-oleoyl-3(1)-lauroyl-glycerol	TG 18:2_18:1 (*sn*-2)_12:0
1(3)-oleoyl-2-lauroyl-3(1)-linoleoyl-glycerol	TG 18:1_12:0 (*sn*-2)_18:2
1(3)-oleoyl-2-linoleoyl-3(1)-lauroyl-glycerol	TG 18:2_18:1 (*sn*-2)_12:0
1(3)-oleoyl-2-palmitoyl-3(1)-stearoyl-glycerol	TG 18:1_16:0 (*sn*-2)_18:0
1(3)-palmitoyl-2-stearoyl-3(1)-oleoyl-glycerol	TG 16:0_18:0 (*sn*-2)_18:1
1(3)-palmitoyl-2-oleoyl-3(1)-stearoyl-glycerol	TG 16:0_18:1 (*sn*-2)_18:0
1(3)-palmitoyl-2-oleoyl-3(1)-linoleoyl-glycerol	TG 16:0_18:1 (*sn*-2)_18:2
1(3)-oleoyl-2-linoleoyl-3(1)-palmitoyl-glycerol	TG 18:1_18:2 (*sn*-2)_16:0
1(3)-oleoyl-2-palmitoyl-3(1)-linoleoyl-glycerol	TG 18:1_16:0 (*sn*-2)_18:2
1(3)-palmitoyl-2-oleoyl-3(1)-arachidonyl-glycerol	TG 16:0_18:1 (*sn*-2)_20:0
1(3)-oleoyl-2-arachidonyl-3(1)-palmitoyl-glycerol	TG 18:1_20:0 (*sn*-2)_16:0
1(3)-oleoyl-2-palmitoyl-3(1)-arachidonyl-glycerol	TG 18:1_16:0 (*sn*-2)_20:0

Calibration results of the fragmentation model

Using the LOOCV models, which always leaves out the calibration data of TG regioisomer for which the result is calculated, the results on average for AAB/ABA type TGs were 94.52 ± 2.52 %, which is close to the theoretical 100 % purity of the standard. Using the final model, the average was 95.42 ± 2.33 %. For the ABC type TGs the accuracy was even higher, 96.18 ± 0.74% for the LOOCV models, and 96.82 ± 0.77% for the final model containing all standards. Overall, there was only a modest improvement in the final model compared to the LOOCV models, indicating that the contribution of a single TG standard to the model is relatively low. This highlights the fact that the model can reasonably accurately estimate fragment ion ratios for TGs that are not included in the calibration data. The least accurate results were calculated for TG 16:0_16:0 (sn-2)_4:0 (72.75 ± 17.24%) and TG 18:1_18:1 (sn-2)_4:0 (78.94 ± 13.29%). These two TGs contain short chain FAs, and they also gained proportionally better accuracy in the final model versus the LOOCV models compared to other TGs. This could suggest that a higher number of TGs containing short-chain FAs would still have been beneficial for the model, but there were also certain TGs containing short-chain FAs for which the results were quite accurate (over 90%).

It is also worth noting that tor standards that have the calculated regioisomer abundance very close to 100% (>99%), the calculated very low standard deviation does not always reflect the instrumental deviation of the fragment ion abundances. This is caused by the fact that the calculated results can technically be over 100% if the observed fragment ion abundances are beyond the end points of the calibration data. However, such result is reported as 100%. Therefore, the observed standard deviation in some of the calculated results is very low even if there would be higher instrumental deviation in the fragment ion abundances.

Table S2 Calibration results for the LOOCV models and the final model, showing the calculated abundance within a pair for each AAB/ABA type regioisomer standard.

		**LOOCV model calibration**		**Final model calibration**	
**Group**	**Regioisomer standard**	**Result (%)**	**Standard deviation**	**Result (%)**	**Standard deviation**
**TG 4:0_4:0_X**	TG 4:0_4:0 (*sn*-2)_16:0	90.69	3.56	91.96	0.26
	TG 4:0_16:0 (*sn*-2)_4:0	99.94	0.02	99.92	0.05
	TG 4:0_4:0 (*sn*-2)_18:0	77.71	15.50	86.08	14.76
	TG 4:0_18:0 (*sn*-2)_4:0	99.77	0.13	99.79	0.05
**TG 10:0_10:0_X**	TG 10:0_10:0 (*sn*-2)_14:0	99.62	0.09	99.73	0.09
	TG 10:0_14:0 (*sn*-2)_10:0	90.06	7.28	91.50	7.56
	TG 10:0_10:0 (*sn*-2)_16:0	99.61	0.08	99.72	0.16
	TG 10:0_16:0 (*sn*-2)_10:0	94.22	4.13	95.25	4.09
	TG 10:0_10:0 (*sn*-2)_18:0	99.74	0.07	99.69	0.05
	TG 10:0_18:0 (*sn*-2)_10:0	97.43	1.94	97.88	1.92
	TG 10:0_10:0 (*sn*-2)_18:1	99.19	0.34	99.27	0.31
	TG 10:0_18:1 (*sn*-2)_10:0	95.71	3.84	96.58	3.85
**TG 16:0_16:0_X**	TG 16:0_16:0 (*sn*-2)_4:0	68.48	16.26	72.75	17.24
	TG 16:0_4:0 (*sn*-2)_16:0	99.93	0.02	99.94	0.01
	TG 16:0_16:0 (*sn*-2)_8:0	99.47	0.02	99.55	0.02
	TG 16:0_8:0 (*sn*-2)_16:0	82.69	6.96	86.44	1.00
	TG 16:0_16:0 (*sn*-2)_14:0	99.40	0.04	99.48	0.06
	TG 16:0_14:0 (*sn*-2)_16:0	92.44	4.30	92.83	4.50
	TG 16:0_16:0 (*sn*-2)_18:0	99.68	0.11	99.70	0.09
	TG 16:0_18:0 (*sn*-2)_16:0	99.98	0.00	99.96	0.02

	TG 16:0_16:0 (*sn*-2)_18:1	99.14	0.06	99.20	0.06
	TG 16:0_18:1 (*sn*-2)_16:0	98.93	0.53	98.83	0.56
	TG 16:0_16:0 (*sn*-2)_18:2	99.36	0.03	99.53	0.14
	TG 16:0_18:2 (*sn*-2)_16:0	95.19	2.11	96.10	2.09
**TG 18:0_18:0_X**	TG 18:0_18:0 (*sn*-2)_16:0	99.57	0.02	99.57	0.01
	TG 18:0_16:0 (*sn*-2)_18:0	99.90	0.01	99.98	0.00
	TG 18:0_18:0 (*sn*-2)_18:1	98.34	0.14	98.52	0.11
	TG 18:0_18:1 (*sn*-2)_18:0	90.82	4.09	91.28	3.58
	TG 18:0_18:0 (*sn*-2)_18:2	99.27	0.21	99.41	0.22
	TG 18:0_18:2 (*sn*-2)_18:0	99.79	0.11	99.80	0.13
**TG 18:1_18:1_X**	TG 18:1_18:1 (*sn*-2)_4:0	71.97	12.68	78.94	13.29
	TG 18:1_4:0 (*sn*-2)_18:1	99.89	0.01	99.83	0.13
	TG 18:1_18:1 (*sn*-2)_12:0	98.89	0.28	98.97	0.23
	TG 18:1_12:0 (*sn*-2)_18:1	92.23	4.82	93.53	4.82
	TG 18:1_18:1 (*sn*-2)_14:0	99.43	0.14	99.34	0.14
	TG 18:1_14:0 (*sn*-2)_18:1	97.99	1.38	98.04	1.38
	TG 18:1_18:1 (*sn*-2)_16:0	99.49	0.15	99.09	0.30
	TG 18:1_16:0 (*sn*-2)_18:1	99.93	0.10	99.98	0.01
	TG 18:1_18:1 (*sn*-2)_18:0	99.15	0.38	99.08	0.32
	TG 18:1_18:0 (*sn*-2)_18:1	99.95	0.03	99.94	0.04
	TG 18:1_18:1 (*sn*-2)_18:2	90.47	6.02	91.00	5.96
	TG 18:1_18:2 (*sn*-2)_18:1	92.07	4.04	92.69	4.07
	TG 18:1_18:1 (*sn*-2)_18:3	74.91	9.03	79.77	8.43
	TG 18:1_18:3 (*sn*-2)_18:1	99.96	0.00	99.94	0.01
**TG 18:2_18:2_X**	TG 18:2_18:2 (*sn*-2)_18:1	98.83	0.01	98.94	0.01
	TG 18:2_18:1 (*sn*-2)_18:2	81.65	1.76	84.72	1.84
	TG 18:2_18:2 (*sn*-2)_18:3	95.79	1.65	96.22	1.67
	TG 18:2_18:3 (*sn*-2)_18:2	78.51	6.43	79.78	6.44
	**Average**	**94.52**	**2.52**	**95.42**	**2.33**

Table S3 Calibration results for the LOOCV models and the final model, showing the calculated abundance within a triplet for each ABC type regioisomer standard.

**ABC type**	**LOOCV model calibration**		**Final model calibration**	
**Regioisomer**	**Result (%)**	**Standard deviation**	**Result (%)**	**Standard deviation**
TG 4:0_16:0 (*sn*-2)_18:1	96.66	3.72	95.47	3.45
TG 4:0_18:1 (*sn*-2)_16:0	99.87	0.09	99.98	0.01
TG 16:0_4:0 (*sn*-2)_18:1	99.40	0.13	97.15	2.17
TG 6:0_16:0 (*sn*-2)_18:1	93.47	0.39	94.91	0.38
TG 6:0_18:1 (*sn*-2)_16:0	98.51	0.13	99.17	0.07
TG 16:0_6:0 (*sn*-2)_18:1	91.80	2.38	94.50	2.06
TG 18:1_18:2 (*sn*-2)_12:0	92.08	1.44	93.75	1.43
TG 18:1_12:0 (*sn*-2)_18:2	92.38	1.18	94.12	1.07
TG 18:2_18:1 (*sn*-2)_12:0	95.34	1.19	97.17	1.09
TG 18:1_16:0 (*sn*-2)_20:0	99.95	0.06	99.88	0.07
TG 18:1_20:0 (*sn*-2)_16:0	99.88	0.04	99.64	0.37
TG 16:0_18:1 (*sn*-2)_20:0	99.94	0.04	99.55	0.31
TG 18:1_16:0 (*sn*-2)_18:2	92.22	1.42	93.62	1.17
TG 18:1_18:2 (*sn*-2)_16:0	94.91	0.19	94.96	0.14
TG 16:0_18:1 (*sn*-2)_18:2	98.62	0.53	98.89	0.05
TG 18:1_16:0 (*sn*-2)_12:0	93.63	0.66	95.72	0.60
TG 18:1_12:0 (*sn*-2)_16:0	96.37	0.42	97.05	0.46
TG 16:0_18:1 (*sn*-2)_12:0	92.47	0.41	94.52	0.32
TG 16:0_18:1 (*sn*-2)_18:0	93.47	0.58	94.17	0.45
TG 16:0_18:0 (*sn*-2)_18:1	99.27	0.40	99.09	0.48
TG 18:1_16:0 (*sn*-2)_18:0	99.55	0.16	99.81	0.12
**Average**	**96.18**	**0.74**	**96.82**	**0.77**

Table S4 FA composition in eight mammalian milk species expressed as relative mass percentage (%).

			Pseudo-ruminant	Ruminants			Non-ruminants			
**Trivial name (in elution order)**	Species level	DB-position level	Camel	Cow	Goat	Sheep	**Human**	Dog	Horse	Pig
Butyric acid	4:0	4:0	0.7 ± 0.1	1.6 ± 0.1	1.0 ± 0.1	1.5 ± 0.1	0.0 ± 0.1	0.1 ± 0.0	0.3 ± 0.1	0.2 ± 0.0
Hexanoic acid	6:0	6:0	0.1 ± 0.0	1.8 ± 0.0	1.6 ± 0.1	2.3 ± 0.1	0.2 ± 0.0	0.1 ± 0.0	0.2 ± 0.0	nd
Caprylic acid	8:0	8:0	0.1 ± 0.0	1.6 ± 0.0	2.8 ± 0.2	3.3 ± 0.1	0.1 ± 0.0	0.0 ± 0.0	1.9 ± 0.2	nd
**Capric acid**	**10:0**	**10:0**	0.2 ± 0.0	3.6 ± 0.1	10.7 ± 0.6[d]	9.8 ± 0.2[d]	1.2 ± 0.0	0.1 ± 0.0	4.9 ± 0.3[e]	nd
Undecanoic acid	11:0	11:0	[1]nd	0.1 ± 0.0	0.1 ± 0.0	0.2 ± 0.0	nd	nd	nd	nd
**Lauric acid**	**12:0**	**12:0**	1.2 ± 0.1	4.1 ± 0.1[e]	5.8 ± 0.3	5.4 ± 0.0[e]	6.0 ± 0.2	0.3 ± 0.0	6.1 ± 0.2[e]	0.0 ± 0.0
Tridecylic acid	13:0	13:0	nd	0.1 ± 0.0	0.1 ± 0.0	0.7 ± 0.9	0.0 ± 0.0	nd	0.0 ± 0.0	nd
**Myristic acid**	**14:0**	**14:0**	11.4 ± 0.4[d]	12.9 ± 0.1[c]	12.2 ± 0.2[c]	9.9 ± 0.1[d]	7.2 ± 0.1[d]	3.3 ± 0.0	7.8 ± 0.1[d]	1.6 ± 0.0
Myristoleic acid	14:1	14:1(9)	0.5 ± 0.0	1.1 ± 0.0	0.3 ± 0.0	0.1 ± 0.0	0.3 ± 0.0	0.2 ± 0.0	0.6 ± 0.0	nd
Pentadecanoic acid	15:0	15:0	1.8 ± 0.0	1.2 ± 0.0	1.2 ± 0.0	0.8 ± 0.0	0.3 ± 0.0	0.1 ± 0.0	0.4 ± 0.0	0.2 ± 0.0
	15:1	15:1	0.4 ± 0.0	0.3 ± 0.0	0.3 ± 0.0	nd	nd	nd	0.3 ± 0.0	nd
**Palmitic acid**	**16:0**	**16:0**	25.5 ± 0.7[a]	35.2 ± 0.2[a]	27.5 ± 0.5[a]	21.9 ± 0.5[b]	19.9 ± 0.2[b]	26.4 ± 0.0[b]	24.8 ± 0.3[a]	24.9 ± 0.2[b]
**Palmitoleic acid**	**16:1**	**16:1(9)**	4.4 ± 0.1[e]	1.5 ± 0.0	0.8 ± 0.0	0.7 ± 0.0	2.4 ± 0.0	5.5 ± 0.0[d]	8.1 ± 0.2	2.4 ± 0.0
Margaric acid	17:0	17:0	1.3 ± 0.0	0.7 ± 0.0	0.7 ± 0.0	0.6 ± 0.0	0.3 ± 0.0	0.2 ± 0.0	0.3 ± 0.0	0.4 ± 0.0
Heptadecenoic acid	17:1	17:1	0.5 ± 0.0	0.2 ± 0.1	0.3 ± 0.1	0.2 ± 0.0	0.1 ± 0.0	0.2 ± 0.0	0.4 ± 0.0	nd
**Stearic acid**	**18:0**	**18:0**	18.3 ± 0.4[c]	10.3 ± 0.1[d]	8.7 ± 0.1[e]	11.3 ± 0.3[c]	6.7 ± 0.0[e]	4.9 ± 0.0[e]	1.8 ± 0.0	6.8 ± 0.1[d]
**Vaccenic acid**	**18:1**	**18:1(11)**	1.4 ± 0.1	0.6 ± 0.0	0.5 ± 0.0	0.8 ± 0.0	2.0 ± 0.0	2.2 ± 0.0	1.4 ± 0.0	2.7 ± 0.0[e]
trans-Oleic acid	18:1	18:1(9E)	0.8 ± 0.0	0.4 ± 0.0	0.3 ± 0.0	0.8 ± 0.0	0.2 ± 0.0	0.2 ± 0.1	0.1 ± 0.0	0.0 ± 0.0
**Oleic acid**	**18:1**	**18:1(9Z)**	22.2 ± 1.6[b]	18.9 ± 0.1[b]	20.6 ± 0.8[b]	24.0 ± 0.6[a]	38.5 ± 0.3[a]	35.7 ± 0.0[a]	19.4 ± 0.4[b]	33.9 ± 0.1[a]
trans-Linoleic acid	18:2	18:2(9E,12E)	1.1 ± 0.1	0.4 ± 0.0	0.5 ± 0.0	0.8 ± 0.0	0.1 ± 0.0	nd	nd	nd
**Linoleic acid (LA)**	**18:2**	**18:2(9Z,12Z)**	4.3 ± 0.1[e]	1.5 ± 0.0	1.9 ± 0.0	2.3 ± 0.0	10.1 ± 0.1[c]	15.1 ± 0.0[c]	5.6 ± 0.1[e]	21.1 ± 0.2[c]
γ-Linolenic acid	18:3	18:3(6,9,12)	nd	0.0 ± 0.0	0.0 ± 0.0	0.0 ± 0.0	0.1 ± 0.0	0.2 ± 0.0	0.4 ± 0.0	0.4 ± 0.0
**α-Linolenic acid (ALA)**	**18:3**	**18:3(9,12,15)**	2.1 ± 0.0	0.6 ± 0.0	0.9 ± 0.0	0.9 ± 0.0	1.9 ± 0.0	1.6 ± 0.0	12.7 ± 0.3[c]	1.5 ± 0.0
Arachidic acid	20:0	20:0	0.5 ± 0.0	0.2 ± 0.0	0.3 ± 0.0	0.3 ± 0.0	0.2 ± 0.0	0.1 ± 0.0	0.1 ± 0.1	0.1 ± 0.0
Gadoleic acid	20:1	20:1(11)	0.1 ± 0.0	0.0 ± 0.0	0.1 ± 0.0	0.3 ± 0.2	0.5 ± 0.0	0.4 ± 0.0	0.2 ± 0.0	0.3 ± 0.0
Eicosadienoic acid	20:2	20:2(11,14)	0.1 ± 0.0	0.0 ± 0.0	0.0 ± 0.0	0.0 ± 0.0	0.3 ± 0.0	0.4 ± 0.0	0.1 ± 0.0	0.5 ± 0.0
Dihomo-γ-linolenic acid	20:3	20:3(8,11,14)	[2]0.0 ± 0.0	0.1 ± 0.0	0.0 ± 0.0	0.0 ± 0.0	0.4 ± 0.0	0.2 ± 0.0	0.0 ± 0.0	0.3 ± 0.0
Eicosatrienoic acid	20:3	20:3(11,14,17)	0.1 ± 0.0	0.0 ± 0.0	nd	nd	0.1 ± 0.0	nd	0.3 ± 0.0	0.1 ± 0.0
Arachidonic acid (ARA)	20:4	20:4(5,8,11,14)	0.1 ± 0.0	0.1 ± 0.0	0.1 ± 0.0	0.2 ± 0.0	0.3 ± 0.0	1.2 ± 0.0	0.1 ± 0.0	1.0 ± 0.0
Eicosapentaenoic acid (EPA)	20:5	20:5(5,8,11,14,17)	0.1 ± 0.0	0.1 ± 0.0	0.1 ± 0.0	0.2 ± 0.0	0.1 ± 0.0	0.4 ± 0.0	0.0 ± 0.0	0.1 ± 0.0
Behenic acid	22:0	22:0	0.2 ± 0.0	0.1 ± 0.0	0.1 ± 0.0	0.1 ± 0.0	0.1 ± 0.0	nd	nd	0.1 ± 0.0
Gondoic acid	22:1	22:1(13)	0.0 ± 0.0	0.0 ± 0.0	0.0 ± 0.0	0.0 ± 0.0	0.1 ± 0.0	nd	0.2 ± 0.0	0.1 ± 0.0
Docosadienoic acid	22:2	22:2(13,16)	nd	nd	nd	nd	0.0 ± 0.0	nd	0.0 ± 0.0	nd
Tricosanoic acid	23:0	23:0	nd	0.0 ± 0.0	0.1 ± 0.0	nd	nd	nd	nd	nd
Adrenic acid	22:4	22:4(7,10,13,16)	0.1 ± 0.0	0.0 ± 0.0	nd	0.1 ± 0.0	0.1 ± 0.0	nd	nd	0.2 ± 0.0
Lignoceric acid	24:0	24:0	0.1 ± 0.0	0.1 ± 0.0	0.1 ± 0.0	nd	0.1 ± 0.0	nd	nd	0.1 ± 0.0
Docosapentaenoic acid (DPA)	22:5	22:5(7,10,13,16,19)	0.4 ± 0.0	0.1 ± 0.0	0.2 ± 0.0	0.5 ± 0.0	0.1 ± 0.0	0.5 ± 0.0	0.4 ± 0.1	0.7 ± 0.0
Docosahexaenoic acid (DHA)	22:6	22:6(4,7,10,13,16,19)	0.1 ± 0.0	0.1 ± 0.0	0.1 ± 0.0	0.2 ± 0.0	0.2 ± 0.0	0.4 ± 0.0	0.1 ± 0.0	0.1 ± 0.0
		SFAs	61.3 ± 1.5[C]	73.7 ± 0.3[A]	73.0 ± 0.9[A]	68.0 ± 0.5[B]	42.3 ± 0.5[E]	35.6 ± 0.1[F]	48.7 ± 0.7[D]	34.5 ± 0.2[F]
		UFAs	38.7 ± 1.5[D]	26.3 ± 0.3[F]	27.0 ± 0.9[F]	32.0 ± 0.5[E]	57.7 ± 0.9[B]	64.4 ± 0.1[A]	51.3 ± 0.7[C]	65.4 ± 0.3[A]
		SCFAs (C4)	0.7 ± 0.1[C]	1.6 ± 0.1[A]	1.0 ± 0.1[B]	1.5 ± 0.1[A]	0.0 ± 0.1[F]	0.1 ± 0.0[E]	0.3 ± 0.1[D]	0.2 ± 0.0[E]
		MUFAs	30.0 ± 1.5[C]	23.0 ± 0.2[E]	22.9 ± 0.8[E]	26.8 ± 0.5[D]	44.2 ± 0.4[A]	44.5 ± 0.1[A]	31.4 ± 0.5[C]	39.6 ± 0.1[B]
		PUFAs	8.3 ± 0.0[D]	3.1 ± 0.1[G]	3.8 ± 0.1[F]	5.2 ± 0.1[E]	13.5 ± 0.1[C]	19.9 ± 0.1[B]	19.7 ± 0.2[B]	26.0 ± 0.2[A]
		n-3 PUFAs	2.6 ± 0.1[C]	0.9 ± 0.0[G]	1.2 ± 0.0[F]	1.8 ± 0.0[E]	2.5 ± 0.0[D]	2.8 ± 0.1[B]	13.4 ± 0.2[A]	2.5 ± 0.0[D]
		n-6 PUFAs	5.6 ± 0.0[E]	2.2 ± 0.0[H]	2.6 ± 0.1[G]	3.4 ± 0.0[F]	11.0 ± 0.1[C]	17.1 ± 0.0[B]	6.2 ± 0.1[D]	23.3 ± 0.2[A]
		n-6/n-3	2.1 ± 0.0[E]	2.4 ± 0.1[D]	2.2 ± 0.1[E]	1.9 ± 0.0[F]	4.4 ± 0.0[C]	6.1 ± 0.1[B]	0.5 ± 0.0[G]	9.5 ± 0.2[A]
		SFAs/UFAs	1.6 ± 0.1[C]	2.8 ± 0.0[A]	2.7 ± 0.1[A]	2.1 ± 0.0[B]	0.0 ± 0.0[F]	0.6 ± 0.0[E]	0.9 ± 0.0[D]	0.5 ± 0.0[E]
		SFAs/PUFAs	7.4 ± 0.1[D]	24.1 ± 0.5[A]	19.1 ± 0.6[B]	13.0 ± 0.2[C]	0.0 ± 0.0[G]	1.8 ± 0.0[F]	2.5 ± 0.1[E]	1.3 ± 0.0[F]
		MCFAs (c6-12)	1.6 ± 0.1[E]	11.2 ± 0.2[C]	21.0 ± 1.1[A]	20.9 ± 0.4[A]	7.4 ± 0.3[D]	0.4 ± 0.0[F]	13.1 ± 0.8[B]	0.0 ± 0.0[F]
		LCFAs (C13-20)	97.0 ± 0.0[B]	86.7 ± 0.2[D]	77.4 ± 1.2[F]	76.8 ± 0.5[F]	91.8 ± 0.2[C]	98.6 ± 0.0[A]	84.8 ± 0.9[E]	98.4 ± 0.1[AB]
		VLCFAs (C22-24)	0.8 ± 0.0[B]	0.5 ± 0.0[B]	0.5 ± 0.1[B]	0.9 ± 0.1[B]	0.7 ± 0.0[B]	0.8 ± 0.0[B]	1.7 ± 0.5[A]	1.4 ± 0.1[A]

[1] nd Not detected.

[2] 0.0 ± 0.0 Values are lower than 0.05.

Lowercases a-e represent comparison among the abundance of FAs in one milk species. Uppercases A-H represent comparison among eight milk species.

Abbreviations: LCFA, long-chain fatty caid; MCFA, medium-chain fatty acid; MUFA, monounsaturated fatty acid; PUFA, polyunsaturated fatty acid; SFA, saturated fatty acid; SCFA, short-chain fatty acid; UFA, unsaturated fatty acid. VLCFA, very long-chain fatty acid.

DB-position level denotation is used when referring to FA composition analysis. Species level is for the regiospecific-level discussion since the MS/MS method used in this study did not provide information on the location of the DBs. ‘Z’ is omitted in DB-position level when only the cis structure presents at the same species level.

Table S5 TG species and regioisomers composition in eight mammalian milk species (mol%). (Values of TG species are marked with lowercases (a–e) to indicate their abundance within each milk type.)

Quick index

ACN 26

ACN 28

ACN 30

ACN 32

ACN 33

ACN 34

ACN 35

ACN 36

ACN 37

ACN 38

ACN 39

ACN 40

ACN 42

ACN 44

ACN 46

ACN 47

ACN 48

ACN 49

ACN 50

ACN 51

ACN 52

ACN 54

ACN	TG species	TG molecular species	TG regioisomer	Camel		Cow		Goat		Sheep		Human		Dog		Horse		Pig	
				in TG species	of total TGs	in TG species	of total TGs	in TG species	of total TGs	in TG species	of total TGs	in TG species	of total TGs	in TG species	of total TGs	in TG species	of total TGs	in TG species	of total TGs
**26**	**26:0**			**nd**		**0.5 ± 0.0**		**0.4 ± 0.1**		**1.5 ± 0.0**		**nd**		**nd**		**nd**		**nd**	
		4:0_10:0_12:0	10:0_12:0(*sn*-2)_4:0			0.7 ± 0.9	0.0 ± 0.0	11.1 ± 19.2	0.0 ± 0.1	0.0 ± 0.0	0.0 ± 0.0								
			12:0_10:0(*sn*-2)_4:0			20.8 ± 29.4	0.1 ± 0.1	18.7 ± 16.7	0.1 ± 0.1	0.0 ± 0.0	0.0 ± 0.0								
			12:0_4:0(*sn*-2)_10:0			13.0 ± 18.4	0.1 ± 0.1	23.9 ± 18.9	0.1 ± 0.1	47.3 ± 10.1	0.7 ± 0.2								
		4:0_6:0_16:0	16:0_4:0(*sn*-2)_6:0			20.8 ± 29.4	0.1 ± 0.1	16.7 ± 23.6	0.1 ± 0.1	18.3 ± 3.5	0.3 ± 0.1								
			16:0_6:0(*sn*-2)_4:0			30.3 ± 42.8	0.1 ± 0.2	12.8 ± 18.0	0.1 ± 0.1	0.0 ± 0.0	0.0 ± 0.0								
			6:0_16:0(*sn*-2)_4:0			0.0 ± 0.0	0.0 ± 0.0	0.0 ± 0.0	0.0 ± 0.0	0.0 ± 0.0	0.0 ± 0.0								
		4:0_8:0_14:0	14:0_4:0(*sn*-2)_8:0			8.2 ± 14.2	0.0 ± 0.1	10.4 ± 17.4	0.0 ± 0.1	8.8 ± 0.8	0.1 ± 0.0								
			14:0_8:0(*sn*-2)_4:0			8.4 ± 10.2	0.0 ± 0.1	14.1 ± 24.3	0.1 ± 0.1	0.0 ± 0.0	0.0 ± 0.0								
			8:0_14:0(*sn*-2)_4:0			17.3 ± 25.3	0.1 ± 0.1	0.0 ± 0.0	0.0 ± 0.0	0.0 ± 0.0	0.0 ± 0.0								
		6:0_10:0_10:0	10:0_10:0(*sn*-2)_6:0			12.5 ± 17.7	0.1 ± 0.1												
			10:0_6:0(*sn*-2)_10:0			1.8 ± 2.4	0.0 ± 0.0												
		6:0_14:0_6:0	14:0_6:0(*sn*-2)_6:0			15.0 ± 17.7	0.1 ± 0.1	18.0 ± 15.0	0.1 ± 0.1	14.9 ± 4.6	0.2 ± 0.1								
			6:0_14:0(*sn*-2)_6:0			9.7 ± 11.6	0.0 ± 0.1	0.3 ± 0.5	0.0 ± 0.0	0.0 ± 0.0	0.0 ± 0.0								
		6:0_8:0_12:0	12:0_6:0(*sn*-2)_8:0					12.7 ± 7.9	0.1 ± 0.0	12.9 ± 8.7	0.2 ± 0.1								
			12:0_8:0(*sn*-2)_6:0					4.6 ± 7.9	0.0 ± 0.0	0.0 ± 0.0	0.0 ± 0.0								
			8:0_12:0(*sn*-2)_6:0					0.0 ± 0.0	0.0 ± 0.0	0.0 ± 0.0	0.0 ± 0.0								
**28**	**28:0**			**nd**		**1.3 ± 0.1**		**1.6 ± 0.1**		**2.9 ± 0.2**		**nd**		**nd**		**nd**		**nd**	
		4:0_10:0_14:0	10:0_14:0(*sn*-2)_4:0			0.0 ± 0.0	0.0 ± 0.0	6.1 ± 13.5	0.1 ± 0.2	0.0 ± 0.1	0.0 ± 0.0								
			14:0_10:0(*sn*-2)_4:0			1.4 ± 2.5	0.0 ± 0.0	4.0 ± 9.0	0.1 ± 0.1	0.0 ± 0.1	0.0 ± 0.0								
			14:0_4:0(*sn*-2)_10:0			38.2 ± 9.3	0.5 ± 0.2	34.1 ± 19.3	0.6 ± 0.3	56.0 ± 4.2	1.6 ± 0.2								
		4:0_12:0_12:0	12:0_12:0(*sn*-2)_4:0					1.0 ± 2.1	0.0 ± 0.0	0.0 ± 0.1	0.0 ± 0.0								
			12:0_4:0(*sn*-2)_12:0					8.9 ± 5.0	0.1 ± 0.1	7.9 ± 3.9	0.2 ± 0.1								
		4:0_8:0_16:0	16:0_4:0(*sn*-2)_8:0			19.7 ± 17.1	0.3 ± 0.2	11.2 ± 6.1	0.2 ± 0.1	7.7 ± 7.6	0.2 ± 0.2								
			16:0_8:0(*sn*-2)_4:0			11.5 ± 19.9	0.1 ± 0.3	5.3 ± 10.6	0.1 ± 0.2	0.0 ± 0.0	0.0 ± 0.0								
			8:0_16:0(*sn*-2)_4:0			0.0 ± 0.0	0.0 ± 0.0	0.0 ± 0.0	0.0 ± 0.0	0.0 ± 0.0	0.0 ± 0.0								
		6:0_6:0_16:0	16:0_6:0(*sn*-2)_6:0			17.1 ± 3.7	0.2 ± 0.1	12.8 ± 1.3	0.2 ± 0.0	19.5 ± 5.2	0.6 ± 0.2								
			6:0_16:0(*sn*-2)_6:0			0.0 ± 0.0	0.0 ± 0.0	0.0 ± 0.0	0.0 ± 0.0	0.0 ± 0.0	0.0 ± 0.0								
		6:0_8:0_14:0	14:0_6:0(*sn*-2)_8:0			12.1 ± 7.9	0.2 ± 0.1												
			14:0_8:0(*sn*-2)_6:0			0.0 ± 0.0	0.0 ± 0.0												
			8:0_14:0(*sn*-2)_6:0			0.0 ± 0.0	0.0 ± 0.0												
		8:0_10:0_10:0	10:0_10:0(*sn*-2)_8:0					4.4 ± 9.8	0.1 ± 0.2										
			10:0_8:0(*sn*-2)_10:0					8.3 ± 5.1	0.1 ± 0.1										
		8:0_8:0_12:0	12:0_8:0(*sn*-2)_8:0					6.8 ± 4.8	0.1 ± 0.1	10.7 ± 1.6	0.3 ± 0.1								
			8:0_12:0(*sn*-2)_8:0					5.4 ± 10.9	0.1 ± 0.2	0.0 ± 0.1	0.0 ± 0.0								
**30**	**30:0**			**nd**		**2.4 ± 0.1**		**3.1 ± 0.4**[e]		**4.7 ± 0.1**		**nd**		**nd**		**nd**		**nd**	
		4:0_10:0_16:0	10:0_16:0(*sn*-2)_4:0			0.0 ± 0.0	0.0 ± 0.0	0.1 ± 0.3	0.0 ± 0.0	0.0 ± 0.1	0.0 ± 0.0								
			16:0_10:0(*sn*-2)_4:0			5.6 ± 9.7	0.1 ± 0.2	0.0 ± 0.0	0.0 ± 0.0	0.0 ± 0.1	0.0 ± 0.0								
			16:0_4:0(*sn*-2)_10:0			40.3 ± 11.1	1.0 ± 0.3	48.3 ± 6.3	1.5 ± 0.4	49.1 ± 2.8	2.3 ± 0.2								
		4:0_12:0_14:0	12:0_14:0(*sn*-2)_4:0			0.0 ± 0.0	0.0 ± 0.0	0.0 ± 0.0	0.0 ± 0.0	0.0 ± 0.1	0.0 ± 0.0								
			14:0_12:0(*sn*-2)_4:0			0.0 ± 0.0	0.0 ± 0.0	0.1 ± 0.1	0.0 ± 0.0	0.0 ± 0.1	0.0 ± 0.0								
			14:0_4:0(*sn*-2)_12:0			28.4 ± 1.4	0.7 ± 0.1	18.4 ± 10.0	0.6 ± 0.4	9.9 ± 3.7	0.5 ± 0.2								
		6:0_10:0_14:0	10:0_14:0(*sn*-2)_6:0					0.0 ± 0.0	0.0 ± 0.0	0.2 ± 0.5	0.0 ± 0.0								
			14:0_10:0(*sn*-2)_6:0					0.0 ± 0.0	0.0 ± 0.0	0.2 ± 0.3	0.0 ± 0.0								
			14:0_6:0(*sn*-2)_10:0					19.2 ± 1.4	0.6 ± 0.1	8.0 ± 0.9	0.4 ± 0.0								
		6:0_12:0_12:0	12:0_12:0(*sn*-2)_6:0							0.1 ± 0.1	0.0 ± 0.0								
			12:0_6:0(*sn*-2)_12:0							8.0 ± 2.7	0.4 ± 0.1								
		6:0_6:0_18:0	18:0_6:0(*sn*-2)_6:0			5.5 ± 0.7	0.1 ± 0.0	5.5 ± 5.5	0.2 ± 0.2	7.9 ± 1.3	0.4 ± 0.1								
			6:0_18:0(*sn*-2)_6:0			0.0 ± 0.0	0.0 ± 0.0	0.0 ± 0.0	0.0 ± 0.0	0.0 ± 0.1	0.0 ± 0.0								
		6:0_8:0_16:0	16:0_6:0(*sn*-2)_8:0			4.9 ± 8.5	0.1 ± 0.2	16.6 ± 2.0	0.5 ± 0.1	5.4 ± 3.4	0.3 ± 0.2								
			16:0_8:0(*sn*-2)_6:0			15.4 ± 13.4	0.4 ± 0.3	1.0 ± 1.8	0.0 ± 0.1	0.5 ± 0.8	0.0 ± 0.0								
			8:0_16:0(*sn*-2)_6:0			0.0 ± 0.0	0.0 ± 0.0	0.0 ± 0.0	0.0 ± 0.0	0.5 ± 1.1	0.0 ± 0.1								
		8:0_8:0_14:0	14:0_8:0(*sn*-2)_8:0							10.0 ± 4.3	0.5 ± 0.2								
			8:0_14:0(*sn*-2)_8:0							0.0 ± 0.1	0.0 ± 0.0								
	**30:1**			**nd**		**0.3 ± 0.3**		**0.1 ± 0.0**		**1.1 ± 0.1**		**nd**		**nd**		**nd**		**nd**	
		4:0_10:0_16:1	10:0_16:1(*sn*-2)_4:0					0.0 ± 0.0	0.0 ± 0.0										
			16:1_10:0(*sn*-2)_4:0					0.0 ± 0.0	0.0 ± 0.0										
			16:1_4:0(*sn*-2)_10:0					16.5 ± 1.5	0.0 ± 0.0										
		4:0_8:0_18:1	18:1_4:0(*sn*-2)_8:0			0.0 ± 0.0	0.0 ± 0.0	14.7 ± 12.2	0.0 ± 0.0	7.3 ± 14.6	0.1 ± 0.2								
			18:1_8:0(*sn*-2)_4:0			58.7 ± 6.2	0.2 ± 0.2	55.3 ± 22.0	0.0 ± 0.0	73.6 ± 9.8	0.8 ± 0.2								
			8:0_18:1(*sn*-2)_4:0			8.7 ± 8.7	0.0 ± 0.1	9.2 ± 13.3	0.0 ± 0.0	19.2 ± 16.0	0.2 ± 0.2								
		6:0_6:0_18:1	18:1_6:0(*sn*-2)_6:0			18.3 ± 19.2	0.1 ± 0.1	12.4 ± 17.5	0.0 ± 0.0										
			6:0_18:1(*sn*-2)_6:0			14.3 ± 12.5	0.0 ± 0.1	12.8 ± 18.0	0.0 ± 0.0										

ACN	TG species	TG molecular species	TG regioisomer	Camel		Cow		Goat		Sheep		Human		Dog		Horse		Pig	
				in TG species	of total TGs	in TG species	of total TGs	in TG species	of total TGs	in TG species	of total TGs	in TG species	of total TGs	in TG species	of total TGs	in TG species	of total TGs	in TG species	of total TGs
**32**	**32:0**			**nd**		**4.6 ± 0.2**^{d}		**4.7 ± 0.2**^{d}		**6.1 ± 0.5**^{c}		**nd**		**nd**		**0.8 ± 0.0**		**nd**	
		10:0_10:0_12:0	10:0_12:0(*sn*-2)_10:0					3.6 ± 2.9	0.2 ± 0.1							13.2 ± 13.7	0.1 ± 0.1		
			12:0_10:0(*sn*-2)_10:0					4.5 ± 1.7	0.2 ± 0.1							11.8 ± 18.4	0.1 ± 0.1		
		4:0_10:0_18:0	10:0_18:0(*sn*-2)_4:0			2.2 ± 4.0	0.1 ± 0.2	2.8 ± 2.2	0.1 ± 0.1	0.1 ± 0.1	0.0 ± 0.0								
			18:0_10:0(*sn*-2)_4:0			6.3 ± 3.6	0.3 ± 0.2	1.1 ± 0.8	0.0 ± 0.0	0.1 ± 0.1	0.0 ± 0.0								
			18:0_4:0(*sn*-2)_10:0			0.9 ± 1.7	0.0 ± 0.1	4.3 ± 1.2	0.2 ± 0.1	14.4 ± 1.9	0.9 ± 0.2								
		4:0_12:0_16:0	12:0_16:0(*sn*-2)_4:0			0.1 ± 0.2	0.0 ± 0.0	2.3 ± 1.9	0.1 ± 0.1	0.1 ± 0.1	0.0 ± 0.0					14.2 ± 11.9	0.1 ± 0.1		
			16:0_12:0(*sn*-2)_4:0			15.1 ± 9.9	0.7 ± 0.5	3.6 ± 3.1	0.2 ± 0.2	0.1 ± 0.1	0.0 ± 0.0					0.4 ± 0.6	0.0 ± 0.0		
			16:0_4:0(*sn*-2)_12:0			25.4 ± 7.6	1.2 ± 0.4	19.3 ± 3.8	0.9 ± 0.2	14.1 ± 5.9	0.9 ± 0.4					1.8 ± 3.1	0.0 ± 0.0		
		4:0_14:0_14:0	14:0_14:0(*sn*-2)_4:0			0.1 ± 0.2	0.0 ± 0.0	1.4 ± 1.2	0.1 ± 0.1	0.1 ± 0.1	0.0 ± 0.0								
			14:0_4:0(*sn*-2)_14:0			26.1 ± 2.6	1.2 ± 0.2	17.6 ± 2.3	0.8 ± 0.1	18.6 ± 1.9	1.1 ± 0.2								
		6:0_10:0_16:0	10:0_16:0(*sn*-2)_6:0			0.7 ± 1.8	0.0 ± 0.1	4.6 ± 2.8	0.2 ± 0.1	0.5 ± 0.7	0.0 ± 0.0					3.0 ± 3.0	0.0 ± 0.0		
			16:0_10:0(*sn*-2)_6:0			9.0 ± 3.7	0.4 ± 0.2	1.6 ± 1.1	0.1 ± 0.1	0.2 ± 0.2	0.0 ± 0.0					0.7 ± 1.2	0.0 ± 0.0		
			16:0_6:0(*sn*-2)_10:0			6.6 ± 6.0	0.3 ± 0.3	15.8 ± 9.2	0.7 ± 0.5	32.4 ± 2.2	2.0 ± 0.3					5.3 ± 4.9	0.0 ± 0.0		
		6:0_12:0_14:0	12:0_14:0(*sn*-2)_6:0					0.8 ± 0.4	0.0 ± 0.0	0.2 ± 0.2	0.0 ± 0.0					5.9 ± 6.2	0.0 ± 0.0		
			14:0_12:0(*sn*-2)_6:0					0.7 ± 0.5	0.0 ± 0.0	0.1 ± 0.1	0.0 ± 0.0					2.3 ± 1.6	0.0 ± 0.0		
			14:0_6:0(*sn*-2)_12:0					1.7 ± 0.7	0.1 ± 0.0	0.9 ± 0.0	0.1 ± 0.0					16.0 ± 26.3	0.1 ± 0.2		
		6:0_8:0_18:0	18:0_6:0(*sn*-2)_8:0			0.2 ± 0.3	0.0 ± 0.0	0.8 ± 0.2	0.0 ± 0.0	1.3 ± 1.6	0.1 ± 0.1								
			18:0_8:0(*sn*-2)_6:0			0.6 ± 0.6	0.0 ± 0.0	0.7 ± 0.2	0.0 ± 0.0	0.6 ± 0.5	0.0 ± 0.0								
			8:0_18:0(*sn*-2)_6:0			1.6 ± 1.4	0.1 ± 0.1	1.8 ± 1.3	0.1 ± 0.1	0.7 ± 0.9	0.0 ± 0.1								
		8:0_10:0_14:0	10:0_14:0(*sn*-2)_8:0													11.2 ± 13.5	0.1 ± 0.1		
			14:0_10:0(*sn*-2)_8:0													4.7 ± 6.1	0.0 ± 0.0		
			14:0_8:0(*sn*-2)_10:0													2.5 ± 3.9	0.0 ± 0.0		
		8:0_12:0_12:0	12:0_12:0(*sn*-2)_8:0					0.9 ± 0.7	0.0 ± 0.0	0.1 ± 0.1	0.0 ± 0.0					9.1 ± 7.0	0.1 ± 0.1		
			12:0_8:0(*sn*-2)_12:0					2.1 ± 0.6	0.1 ± 0.0	10.6 ± 3.8	0.6 ± 0.3					7.1 ± 9.8	0.1 ± 0.1		
		8:0_8:0_16:0	16:0_8:0(*sn*-2)_8:0			6.5 ± 2.7	0.3 ± 0.1	7.2 ± 1.3	0.3 ± 0.1	6.0 ± 1.9	0.4 ± 0.1					5.3 ± 9.1	0.0 ± 0.1		
			8:0_16:0(*sn*-2)_8:0			0.0 ± 0.1	0.0 ± 0.0	0.8 ± 0.6	0.0 ± 0.0	0.1 ± 0.1	0.0 ± 0.0					7.9 ± 5.8	0.1 ± 0.0		
	**32:1**			**nd**		**0.3 ± 0.1**		**1.6 ± 0.2**		**2.4 ± 0.1**		**nd**		**nd**		**nd**		**nd**	
		4:0_10:0_18:1	10:0_18:1(*sn*-2)_4:0			0.6 ± 1.2	0.0 ± 0.0	3.8 ± 8.3	0.1 ± 0.1	0.0 ± 0.0	0.0 ± 0.0								
			18:1_10:0(*sn*-2)_4:0			62.8 ± 26.7	0.2 ± 0.1	29.1 ± 16.2	0.5 ± 0.3	44.1 ± 10.7	1.0 ± 0.3								
			18:1_4:0(*sn*-2)_10:0			4.4 ± 8.8	0.0 ± 0.0	31.5 ± 20.9	0.5 ± 0.4	55.9 ± 10.7	1.3 ± 0.3								
		4:0_12:0_16:1	12:0_16:1(*sn*-2)_4:0			0.0 ± 0.0	0.0 ± 0.0	3.4 ± 6.9	0.1 ± 0.1										
			16:1_12:0(*sn*-2)_4:0			0.0 ± 0.0	0.0 ± 0.0	0.0 ± 0.0	0.0 ± 0.0										
			16:1_4:0(*sn*-2)_12:0			6.5 ± 2.2	0.0 ± 0.0	7.0 ± 5.2	0.1 ± 0.1										
		4:0_14:0_14:1	14:0_14:1(*sn*-2)_4:0			5.3 ± 9.2	0.0 ± 0.0												
			14:0_4:0(*sn*-2)_14:1			6.8 ± 6.7	0.0 ± 0.0												
			14:1_14:0(*sn*-2)_4:0			0.0 ± 0.0	0.0 ± 0.0												
		6:0_10:0_16:1	10:0_16:1(*sn*-2)_6:0					3.0 ± 4.9	0.0 ± 0.1										
			16:1_10:0(*sn*-2)_6:0					2.6 ± 4.5	0.0 ± 0.1										
			16:1_6:0(*sn*-2)_10:0					4.1 ± 6.0	0.1 ± 0.1										
		6:0_12:0_14:1	12:0_14:1(*sn*-2)_6:0			6.5 ± 11.3	0.0 ± 0.0												
			14:1_12:0(*sn*-2)_6:0			0.0 ± 0.0	0.0 ± 0.0												
			14:1_6:0(*sn*-2)_12:0			3.7 ± 3.2	0.0 ± 0.0												
		6:0_8:0_18:1	18:1_6:0(*sn*-2)_8:0			16.9 ± 8.4	0.0 ± 0.0	4.1 ± 9.0	0.1 ± 0.1										
			18:1_8:0(*sn*-2)_6:0			7.6 ± 10.7	0.0 ± 0.0	16.7 ± 6.9	0.3 ± 0.1										
			8:0_18:1(*sn*-2)_6:0			0.0 ± 0.0	0.0 ± 0.0	0.6 ± 1.3	0.0 ± 0.0										
**33**	**33:0**			**nd**		**0.2 ± 0.0**		**0.1 ± 0.0**		**nd**		**nd**		**nd**		**nd**		**nd**	
		4:0_12:0_17:0	12:0_17:0(*sn*-2)_4:0					2.6 ± 2.8	0.0 ± 0.0										
			17:0_12:0(*sn*-2)_4:0					0.3 ± 0.4	0.0 ± 0.0										
			17:0_4:0(*sn*-2)_12:0					0.3 ± 0.4	0.0 ± 0.0										
		4:0_14:0_15:0	14:0_15:0(*sn*-2)_4:0			4.3 ± 8.5	0.0 ± 0.0	8.6 ± 14.9	0.0 ± 0.0										
			15:0_14:0(*sn*-2)_4:0			50.8 ± 48.0	0.1 ± 0.1	28.5 ± 24.4	0.0 ± 0.0										
			15:0_4:0(*sn*-2)_14:0			44.9 ± 52.4	0.1 ± 0.1	21.7 ± 26.5	0.0 ± 0.0										
		6:0_10:0_17:0	10:0_17:0(*sn*-2)_6:0					1.4 ± 1.3	0.0 ± 0.0										
			17:0_10:0(*sn*-2)_6:0					6.7 ± 9.4	0.0 ± 0.0										
			17:0_6:0(*sn*-2)_10:0					14.1 ± 10.4	0.0 ± 0.0										
		6:0_12:0_15:0	12:0_15:0(*sn*-2)_6:0					10.4 ± 14.7	0.0 ± 0.0										
			15:0_12:0(*sn*-2)_6:0					8.2 ± 11.6	0.0 ± 0.0										
			15:0_6:0(*sn*-2)_12:0					17.7 ± 20.9	0.0 ± 0.0										

ACN	TG species	TG molecular species	TG regioisomer	Camel		Cow		Goat		Sheep		Human		Dog		Horse		Pig	
				in TG species	of total TGs	in TG species	of total TGs	in TG species	of total TGs	in TG species	of total TGs	in TG species	of total TGs	in TG species	of total TGs	in TG species	of total TGs	in TG species	of total TGs
**34**	**34:0**			**nd**		**9.7 ± 0.4^{b}**		**6.8 ± 0.3^{ab}**		**5.9 ± 0.0^{c}**		**nd**		**nd**		**1.3 ± 0.2**		**nd**	
		10:0_10:0_14:0	10:0_14:0(*sn*-2)_10:0					3.8 ± 4.2	0.3 ± 0.3	1.8 ± 2.2	0.1 ± 0.1					0.0 ± 0.0	0.0 ± 0.0		
			14:0_10:0(*sn*-2)_10:0					0.6 ± 0.9	0.0 ± 0.1	5.9 ± 5.6	0.4 ± 0.3					14.8 ± 7.6	0.2 ± 0.1		
		10:0_12:0_12:0	12:0_10:0(*sn*-2)_12:0							1.5 ± 1.3	0.1 ± 0.1					12.4 ± 3.8	0.2 ± 0.1		
			12:0_12:0(*sn*-2)_10:0							1.3 ± 1.8	0.1 ± 0.1					0.1 ± 0.1	0.0 ± 0.0		
		4:0_12:0_18:0	12:0_18:0(*sn*-2)_4:0			0.1 ± 0.1	0.0 ± 0.0												
			18:0_12:0(*sn*-2)_4:0			2.6 ± 2.4	0.2 ± 0.2												
			18:0_4:0(*sn*-2)_12:0			0.8 ± 1.2	0.1 ± 0.1												
		4:0_14:0_16:0	14:0_16:0(*sn*-2)_4:0			0.0 ± 0.0	0.0 ± 0.0	0.1 ± 0.2	0.0 ± 0.0	0.3 ± 0.4	0.0 ± 0.0								
			16:0_14:0(*sn*-2)_4:0			19.7 ± 13.8	1.9 ± 1.4	3.9 ± 4.8	0.3 ± 0.3	2.0 ± 3.7	0.1 ± 0.2								
			16:0_4:0(*sn*-2)_14:0			55.3 ± 13.4	5.4 ± 1.5	48.9 ± 3.6	3.3 ± 0.4	45.7 ± 1.2	2.7 ± 0.1								
		6:0_10:0_18:0	10:0_18:0(*sn*-2)_6:0			2.1 ± 2.2	0.2 ± 0.2	2.3 ± 3.3	0.2 ± 0.2	2.9 ± 3.4	0.2 ± 0.2								
			18:0_10:0(*sn*-2)_6:0			1.6 ± 2.2	0.2 ± 0.2	0.1 ± 0.2	0.0 ± 0.0	0.3 ± 0.3	0.0 ± 0.0								
			18:0_6:0(*sn*-2)_10:0			2.1 ± 1.9	0.2 ± 0.2	7.7 ± 5.1	0.5 ± 0.4	6.1 ± 3.4	0.4 ± 0.2								
		6:0_12:0_16:0	12:0_16:0(*sn*-2)_6:0			0.1 ± 0.1	0.0 ± 0.0	0.6 ± 1.1	0.0 ± 0.1	0.8 ± 1.0	0.0 ± 0.1								
			16:0_12:0(*sn*-2)_6:0			6.3 ± 5.2	0.6 ± 0.5	0.4 ± 0.6	0.0 ± 0.0	0.5 ± 0.7	0.0 ± 0.0								
			16:0_6:0(*sn*-2)_12:0			6.0 ± 3.0	0.6 ± 0.3	12.6 ± 4.1	0.9 ± 0.3	15.0 ± 6.1	0.9 ± 0.4								
		8:0_10:0_16:0	10:0_16:0(*sn*-2)_8:0			0.1 ± 0.1	0.0 ± 0.0	1.0 ± 1.9	0.1 ± 0.1	1.7 ± 1.9	0.1 ± 0.1					3.2 ± 6.4	0.0 ± 0.1		
			16:0_10:0(*sn*-2)_8:0			0.1 ± 0.1	0.0 ± 0.0	0.3 ± 0.6	0.0 ± 0.0	0.5 ± 0.6	0.0 ± 0.0					0.1 ± 0.3	0.0 ± 0.0		
			16:0_8:0(*sn*-2)_10:0			4.0 ± 3.6	0.4 ± 0.4	17.7 ± 2.5	1.2 ± 0.2	10.2 ± 2.0	0.6 ± 0.1					35.8 ± 9.8	0.5 ± 0.2		
		8:0_12:0_14:0	12:0_14:0(*sn*-2)_8:0													0.1 ± 0.1	0.0 ± 0.0		
			14:0_12:0(*sn*-2)_8:0													0.1 ± 0.2	0.0 ± 0.0		
			14:0_8:0(*sn*-2)_12:0													33.4 ± 10.6	0.4 ± 0.2		
		8:0_8:0_18:0	18:0_8:0(*sn*-2)_8:0			2.0 ± 0.6	0.2 ± 0.1			4.6 ± 3.9	0.3 ± 0.2								
			8:0_18:0(*sn*-2)_8:0			0.0 ± 0.0	0.0 ± 0.0			0.1 ± 0.1	0.0 ± 0.0								
	**34:1**			**nd**		**1.4 ± 0.0**		**2.0 ± 0.1**		**2.3 ± 0.5**		**nd**		**nd**		**nd**		**nd**	
		4:0_12:0_18:1	12:0_18:1(*sn*-2)_4:0			0.0 ± 0.0	0.0 ± 0.0	1.3 ± 2.9	0.0 ± 0.1	1.0 ± 1.7	0.0 ± 0.0								
			18:1_12:0(*sn*-2)_4:0			42.7 ± 5.8	0.6 ± 0.1	28.0 ± 18.5	0.6 ± 0.4	3.7 ± 6.5	0.1 ± 0.2								
			18:1_4:0(*sn*-2)_12:0			5.3 ± 3.6	0.1 ± 0.1	9.2 ± 13.7	0.2 ± 0.3	34.4 ± 4.0	0.8 ± 0.3								
		4:0_14:0_16:1	14:0_16:1(*sn*-2)_4:0					1.5 ± 3.4	0.0 ± 0.1	3.1 ± 3.4	0.1 ± 0.1								
			16:1_14:0(*sn*-2)_4:0					4.2 ± 7.6	0.1 ± 0.2	0.3 ± 0.5	0.0 ± 0.0								
			16:1_4:0(*sn*-2)_14:0					8.8 ± 6.4	0.2 ± 0.1	7.5 ± 4.7	0.2 ± 0.1								
		4:0_14:1_16:0	14:1_16:0(*sn*-2)_4:0			0.0 ± 0.0	0.0 ± 0.0	1.5 ± 2.5	0.0 ± 0.1										
			16:0_14:1(*sn*-2)_4:0			6.2 ± 10.8	0.1 ± 0.2	0.0 ± 0.0	0.0 ± 0.0										
			16:0_4:0(*sn*-2)_14:1			21.4 ± 10.3	0.3 ± 0.2	4.8 ± 3.2	0.1 ± 0.1										
		6:0_10:0_18:1	10:0_18:1(*sn*-2)_6:0			0.0 ± 0.1	0.0 ± 0.0	2.0 ± 2.5	0.0 ± 0.1	6.7 ± 7.7	0.2 ± 0.2								
			18:1_10:0(*sn*-2)_6:0			15.8 ± 5.9	0.2 ± 0.1	27.8 ± 8.4	0.5 ± 0.2	22.8 ± 4.8	0.5 ± 0.2								
			18:1_6:0(*sn*-2)_10:0			1.4 ± 1.5	0.0 ± 0.0	2.2 ± 4.5	0.0 ± 0.1	9.4 ± 7.8	0.2 ± 0.2								
		8:0_8:0_18:1	18:1_8:0(*sn*-2)_8:0			7.2 ± 1.3	0.1 ± 0.0	11.3 ± 4.4	0.2 ± 0.1	10.9 ± 4.0	0.2 ± 0.1								
			8:0_18:1(*sn*-2)_8:0			0.0 ± 0.0	0.0 ± 0.0	0.0 ± 0.1	0.0 ± 0.0	0.1 ± 0.2	0.0 ± 0.0								
**35**	**35:0**			**nd**		**0.3 ± 0.1**		**0.2 ± 0.0**		**0.7 ± 0.0**		**nd**		**nd**		**nd**		**nd**	
		10:0_10:0_15:0	10:0_15:0(*sn*-2)_10:0					8.6 ± 2.1	0.0 ± 0.0	5.2 ± 8.0	0.0 ± 0.1								
			15:0_10:0(*sn*-2)_10:0					0.7 ± 0.8	0.0 ± 0.0	5.3 ± 9.1	0.0 ± 0.1								
		4:0_14:0_17:0	14:0_17:0(*sn*-2)_4:0			0.0 ± 0.0	0.0 ± 0.0	1.5 ± 2.2	0.0 ± 0.0	8.8 ± 15.3	0.1 ± 0.1								
			17:0_14:0(*sn*-2)_4:0			6.1 ± 6.8	0.0 ± 0.0	0.1 ± 0.2	0.0 ± 0.0	0.0 ± 0.0	0.0 ± 0.0								
			17:0_4:0(*sn*-2)_14:0			11.3 ± 10.1	0.0 ± 0.0	11.1 ± 10.0	0.0 ± 0.0	11.3 ± 9.1	0.1 ± 0.1								
		4:0_15:0_16:0	15:0_16:0(*sn*-2)_4:0			0.0 ± 0.0	0.0 ± 0.0	0.1 ± 0.1	0.0 ± 0.0	0.0 ± 0.0	0.0 ± 0.0								
			16:0_15:0(*sn*-2)_4:0			10.9 ± 11.8	0.0 ± 0.1	31.3 ± 22.3	0.1 ± 0.0	14.1 ± 15.7	0.1 ± 0.1								
			16:0_4:0(*sn*-2)_15:0			63.5 ± 8.8	0.2 ± 0.1	20.3 ± 27.1	0.0 ± 0.0	38.3 ± 15.3	0.3 ± 0.1								
		6:0_12:0_17:0	12:0_17:0(*sn*-2)_6:0					0.1 ± 0.2	0.0 ± 0.0	0.0 ± 0.0	0.0 ± 0.0								
			17:0_12:0(*sn*-2)_6:0					9.7 ± 16.6	0.0 ± 0.0	0.0 ± 0.0	0.0 ± 0.0								
			17:0_6:0(*sn*-2)_12:0					1.5 ± 2.2	0.0 ± 0.0	9.6 ± 8.6	0.1 ± 0.1								
		6:0_14:0_15:0	14:0_15:0(*sn*-2)_6:0			6.1 ± 8.0	0.0 ± 0.0	8.2 ± 6.0	0.0 ± 0.0	7.5 ± 10.6	0.1 ± 0.1								
			15:0_14:0(*sn*-2)_6:0			3.6 ± 3.2	0.0 ± 0.0	0.1 ± 0.2	0.0 ± 0.0	0.0 ± 0.0	0.0 ± 0.0								
			15:0_6:0(*sn*-2)_14:0			1.2 ± 2.1	0.0 ± 0.0	3.8 ± 6.6	0.0 ± 0.0	9.4 ± 13.4	0.1 ± 0.1								
		8:0_10:0_17:0	10:0_17:0(*sn*-2)_8:0					8.0 ± 3.6	0.0 ± 0.0	0.0 ± 0.0	0.0 ± 0.0								
			17:0_10:0(*sn*-2)_8:0					0.7 ± 1.0	0.0 ± 0.0	0.0 ± 0.0	0.0 ± 0.0								
			17:0_8:0(*sn*-2)_10:0					0.9 ± 1.3	0.0 ± 0.0	13.5 ± 0.3	0.1 ± 0.0								
		8:0_12:0_15:0	12:0_15:0(*sn*-2)_8:0					6.5 ± 1.0	0.0 ± 0.0	5.1 ± 1.9	0.0 ± 0.0								
			15:0_12:0(*sn*-2)_8:0					0.6 ± 0.8	0.0 ± 0.0	0.0 ± 0.0	0.0 ± 0.0								
			15:0_8:0(*sn*-2)_12:0					0.8 ± 1.1	0.0 ± 0.0	4.2 ± 5.9	0.0 ± 0.0								

ACN	TG species	TG molecular species	TG regioisomer	Camel		Cow		Goat		Sheep		Human		Dog		Horse		Pig	
				in TG species	of total TGs	in TG species	of total TGs	in TG species	of total TGs	in TG species	of total TGs	in TG species	of total TGs	in TG species	of total TGs	in TG species	of total TGs	in TG species	of total TGs
**36**	**36:0**			**nd**		**14.8 ± 0.4[a]**		**7.3 ± 1.3[a]**		**6.9 ± 0.2[b]**		**nd**		**1.0 ± 0.2**		**1.9 ± 0.1**		**nd**	
		10:0_10:0_16:0	10:0_16:0(*sn*-2)_10:0					11.5 ± 4.9	0.8 ± 0.5	9.5 ± 0.8	0.7 ± 0.1					13.5 ± 5.8	0.3 ± 0.1		
			16:0_10:0(*sn*-2)_10:0					3.3 ± 2.1	0.2 ± 0.2	1.9 ± 1.0	0.1 ± 0.1					5.5 ± 5.7	0.1 ± 0.1		
		10:0_12:0_14:0	12:0_14:0(*sn*-2)_10:0					1.5 ± 0.4	0.1 ± 0.0	1.7 ± 0.5	0.1 ± 0.0					8.6 ± 3.5	0.2 ± 0.1		
			14:0_10:0(*sn*-2)_12:0					1.6 ± 0.6	0.1 ± 0.1	1.6 ± 0.4	0.1 ± 0.0					4.5 ± 3.6	0.1 ± 0.1		
			14:0_12:0(*sn*-2)_10:0					1.9 ± 0.6	0.1 ± 0.1	1.8 ± 0.5	0.1 ± 0.0					10.0 ± 6.2	0.2 ± 0.1		
		12:0_12:0_12:0	12:0_12:0(*sn*-2)_12:0					1.1 ± 0.3	0.1 ± 0.0	1.2 ± 0.2	0.1 ± 0.0					6.7 ± 2.0	0.1 ± 0.0		
		4:0_14:0_18:0	14:0_18:0(*sn*-2)_4:0			7.1 ± 4.4	1.1 ± 0.7	4.4 ± 2.1	0.3 ± 0.2	7.0 ± 2.8	0.5 ± 0.2					2.8 ± 3.5	0.1 ± 0.1		
			18:0_14:0(*sn*-2)_4:0			7.6 ± 2.8	1.1 ± 0.4	2.9 ± 0.7	0.2 ± 0.1	4.9 ± 1.9	0.3 ± 0.1					0.3 ± 0.3	0.0 ± 0.0		
			18:0_4:0(*sn*-2)_14:0			1.3 ± 1.1	0.2 ± 0.2	1.6 ± 1.0	0.1 ± 0.1	1.1 ± 0.8	0.1 ± 0.1					0.3 ± 0.3	0.0 ± 0.0		
		4:0_16:0_16:0	16:0_16:0(*sn*-2)_4:0			57.5 ± 7.1	8.5 ± 1.3	29.4 ± 7.6	2.2 ± 0.9	33.5 ± 4.3	2.3 ± 0.4			99.8 ± 0.2	1.0 ± 0.2	6.5 ± 3.1	0.1 ± 0.1		
			16:0_4:0(*sn*-2)_16:0			5.0 ± 5.3	0.7 ± 0.8	10.0 ± 8.9	0.7 ± 0.8	2.8 ± 3.8	0.2 ± 0.3			0.2 ± 0.2	0.0 ± 0.0	4.8 ± 7.0	0.1 ± 0.1		
		6:0_12:0_18:0	12:0_18:0(*sn*-2)_6:0			1.0 ± 0.4	0.2 ± 0.1	1.1 ± 0.4	0.1 ± 0.0	1.5 ± 0.8	0.1 ± 0.1								
			18:0_12:0(*sn*-2)_6:0			1.8 ± 1.3	0.3 ± 0.2	1.8 ± 0.5	0.1 ± 0.1	2.3 ± 0.5	0.2 ± 0.0								
			18:0_6:0(*sn*-2)_12:0			0.3 ± 0.2	0.0 ± 0.0	1.1 ± 0.5	0.1 ± 0.1	1.1 ± 0.4	0.1 ± 0.0								
		6:0_14:0_16:0	14:0_16:0(*sn*-2)_6:0			4.3 ± 3.3	0.6 ± 0.5	4.3 ± 1.5	0.3 ± 0.2	4.4 ± 0.3	0.3 ± 0.0					3.0 ± 2.7	0.1 ± 0.1		
			16:0_14:0(*sn*-2)_6:0			11.9 ± 3.4	1.8 ± 0.6	11.1 ± 3.5	0.8 ± 0.4	8.5 ± 2.4	0.6 ± 0.2					1.8 ± 1.2	0.0 ± 0.0		
			16:0_6:0(*sn*-2)_14:0			0.8 ± 0.8	0.1 ± 0.1	2.3 ± 1.1	0.2 ± 0.1	2.1 ± 1.3	0.1 ± 0.1					2.2 ± 2.2	0.0 ± 0.0		
		8:0_10:0_18:0	10:0_18:0(*sn*-2)_8:0			1.4 ± 0.5	0.2 ± 0.1	0.8 ± 0.2	0.1 ± 0.0	0.9 ± 0.6	0.1 ± 0.0								
			18:0_10:0(*sn*-2)_8:0			0.5 ± 0.4	0.1 ± 0.1	1.1 ± 0.2	0.1 ± 0.0	1.7 ± 0.2	0.1 ± 0.0								
			18:0_8:0(*sn*-2)_10:0			0.3 ± 0.3	0.1 ± 0.0	1.2 ± 0.3	0.1 ± 0.0	1.3 ± 0.6	0.1 ± 0.0								
		8:0_12:0_16:0	12:0_16:0(*sn*-2)_8:0					0.9 ± 0.1	0.1 ± 0.0	1.7 ± 0.4	0.1 ± 0.0					7.1 ± 1.2	0.1 ± 0.0		
			16:0_12:0(*sn*-2)_8:0					2.1 ± 1.7	0.2 ± 0.1	3.5 ± 2.4	0.2 ± 0.2					10.9 ± 4.2	0.2 ± 0.1		
			16:0_8:0(*sn*-2)_12:0					1.3 ± 0.4	0.1 ± 0.0	1.8 ± 0.3	0.1 ± 0.0					5.6 ± 5.0	0.1 ± 0.1		
		8:0_14:0_14:0	14:0_14:0(*sn*-2)_8:0					1.2 ± 0.4	0.1 ± 0.0	1.3 ± 0.3	0.1 ± 0.0					3.3 ± 1.2	0.1 ± 0.0		
			14:0_8:0(*sn*-2)_14:0					1.1 ± 0.5	0.1 ± 0.0	0.8 ± 0.4	0.1 ± 0.0					2.5 ± 3.0	0.0 ± 0.1		
	**36:1**			**nd**		**3.8 ± 0.2**		**3.5 ± 0.1[c]**		**3.7 ± 0.0**		**nd**		**0.6 ± 0.2**		**0.9 ± 0.1**		**nd**	
		10:0_10:0_16:1	10:0_16:1(*sn*-2)_10:0													7.6 ± 6.4	0.1 ± 0.1		
			16:1_10:0(*sn*-2)_10:0													6.8 ± 8.6	0.1 ± 0.1		
		10:0_12:0_14:1	12:0_14:1(*sn*-2)_10:0													4.3 ± 6.1	0.0 ± 0.1		
			14:1_10:0(*sn*-2)_12:0													5.5 ± 3.7	0.0 ± 0.0		
			14:1_12:0(*sn*-2)_10:0													0.9 ± 0.7	0.0 ± 0.0		
		4:0_14:0_18:1	14:0_18:1(*sn*-2)_4:0			3.1 ± 6.1	0.1 ± 0.2	0.1 ± 0.1	0.0 ± 0.0	0.0 ± 0.0	0.0 ± 0.0					0.3 ± 0.5	0.0 ± 0.0		
			18:1_14:0(*sn*-2)_4:0			55.0 ± 6.6	2.1 ± 0.3	20.6 ± 16.4	0.7 ± 0.6	14.8 ± 5.7	0.6 ± 0.2					0.3 ± 0.4	0.0 ± 0.0		
			18:1_4:0(*sn*-2)_14:0			4.8 ± 8.5	0.2 ± 0.3	17.9 ± 13.2	0.6 ± 0.5	35.2 ± 4.5	1.3 ± 0.2					14.5 ± 3.9	0.1 ± 0.0		
		4:0_16:0_16:1	16:0_16:1(*sn*-2)_4:0			14.5 ± 11.0	0.5 ± 0.4	6.4 ± 10.6	0.2 ± 0.4	5.4 ± 8.0	0.2 ± 0.3			0.0 ± 0.0	0.0 ± 0.0	7.4 ± 12.0	0.1 ± 0.1		
			16:0_4:0(*sn*-2)_16:1			9.3 ± 9.2	0.3 ± 0.4	10.7 ± 7.3	0.4 ± 0.3	8.5 ± 6.8	0.3 ± 0.3			15.7 ± 31.2	0.1 ± 0.2	16.7 ± 11.2	0.1 ± 0.1		
			16:1_16:0(*sn*-2)_4:0			0.0 ± 0.0	0.0 ± 0.0	0.0 ± 0.0	0.0 ± 0.0	0.0 ± 0.0	0.0 ± 0.0			84.3 ± 31.2	0.5 ± 0.4	0.2 ± 0.3	0.0 ± 0.0		
		6:0_12:0_18:1	12:0_18:1(*sn*-2)_6:0			0.2 ± 0.2	0.0 ± 0.0	0.0 ± 0.1	0.0 ± 0.0	0.0 ± 0.0	0.0 ± 0.0								
			18:1_12:0(*sn*-2)_6:0			9.3 ± 2.5	0.3 ± 0.1	17.6 ± 0.9	0.6 ± 0.0	8.5 ± 3.8	0.3 ± 0.1								
			18:1_6:0(*sn*-2)_12:0			1.0 ± 1.9	0.0 ± 0.1	0.5 ± 0.6	0.0 ± 0.0	7.1 ± 1.7	0.3 ± 0.1								
		6:0_14:0_16:1	14:0_16:1(*sn*-2)_6:0					0.6 ± 1.0	0.0 ± 0.0										
			16:1_14:0(*sn*-2)_6:0					0.6 ± 1.0	0.0 ± 0.0										
			16:1_6:0(*sn*-2)_14:0					0.7 ± 1.1	0.0 ± 0.0										
		8:0_10:0_18:1	10:0_18:1(*sn*-2)_8:0			0.9 ± 1.5	0.0 ± 0.1	1.5 ± 3.0	0.1 ± 0.1	0.0 ± 0.0	0.0 ± 0.0					1.2 ± 2.4	0.0 ± 0.0		
			18:1_10:0(*sn*-2)_8:0			2.4 ± 2.2	0.1 ± 0.1	18.7 ± 4.8	0.7 ± 0.2	12.4 ± 5.7	0.5 ± 0.2					5.8 ± 10.7	0.1 ± 0.1		
			18:1_8:0(*sn*-2)_10:0			0.6 ± 1.0	0.0 ± 0.0	4.5 ± 5.4	0.2 ± 0.2	8.0 ± 8.2	0.3 ± 0.3					12.3 ± 7.1	0.1 ± 0.1		
		8:0_12:0_16:1	12:0_16:1(*sn*-2)_8:0													11.3 ± 11.2	0.1 ± 0.1		
			16:1_12:0(*sn*-2)_8:0													0.4 ± 0.5	0.0 ± 0.0		
			16:1_8:0(*sn*-2)_12:0													8.9 ± 5.5	0.1 ± 0.1		
		8:0_14:0_14:1	14:0_14:1(*sn*-2)_8:0													0.4 ± 0.5	0.0 ± 0.0		
			14:0_8:0(*sn*-2)_14:1													1.5 ± 1.1	0.0 ± 0.0		
			14:1_14:0(*sn*-2)_8:0													0.4 ± 0.3	0.0 ± 0.0		
	**36:2**			**nd**		**0.2 ± 0.0**		**nd**		**nd**		**nd**		**nd**		**nd**		**nd**	
		4:0_14:0_18:2	14:0_18:2(*sn*-2)_4:0			0.0 ± 0.0	0.0 ± 0.0												
			18:2_14:0(*sn*-2)_4:0			47.5 ± 35.2	0.1 ± 0.1												
			18:2_4:0(*sn*-2)_14:0			25.1 ± 16.9	0.0 ± 0.0												
		4:0_14:1_18:1	14:1_18:1(*sn*-2)_4:0			0.0 ± 0.0	0.0 ± 0.0												
			18:1_14:1(*sn*-2)_4:0			19.6 ± 20.3	0.0 ± 0.0												
			18:1_4:0(*sn*-2)_14:1			16.9 ± 18.3	0.0 ± 0.0												

ACN	TG species	TG molecular species	TG regioisomer	Camel		Cow		Goat		Sheep		Human		Dog		Horse		Pig	
				in TG species	of total TGs	in TG species	of total TGs	in TG species	of total TGs	in TG species	of total TGs	in TG species	of total TGs	in TG species	of total TGs	in TG species	of total TGs	in TG species	of total TGs
	**36:3**			**nd**		**nd**		**nd**		**nd**		**nd**		**nd**		**0.4 ± 0.1**		**nd**	
		4:0_14:0_18:0	14:0_18:3(*sn*-2)_4:0													29.8 ± 12.9	0.1 ± 0.1		
			18:3_14:0(*sn*-2)_4:0													1.2 ± 1.6	0.0 ± 0.0		
			18:3_4:0(*sn*-2)_14:0													0.0 ± 0.0	0.0 ± 0.0		
		8:0_10:0_18:3	10:0_18:3(*sn*-2)_8:0													7.4 ± 12.6	0.0 ± 0.1		
			18:3_10:0(*sn*-2)_8:0													71.9 ± 30.6	0.3 ± 0.2		
			18:3_8:0(*sn*-2)_10:0													0.1 ± 0.1	0.0 ± 0.0		
**37**	**37:0**			**nd**		**0.1 ± 0.0**		**0.1 ± 0.0**		**0.9 ± 0.0**		**nd**		**nd**		**nd**		**nd**	
		10:0_10:0_17:0	10:0_17:0(*sn*-2)_10:0					0.8 ± 0.6	0.0 ± 0.0	1.3 ± 2.1	0.0 ± 0.0								
			17:0_10:0(*sn*-2)_10:0					1.6 ± 2.2	0.0 ± 0.0	8.5 ± 8.6	0.1 ± 0.1								
		10:0_12:0_15:0	12:0_15:0(*sn*-2)_10:0					16.6 ± 5.5	0.0 ± 0.0	3.5 ± 4.1	0.0 ± 0.0								
			15:0_10:0(*sn*-2)_12:0					2.0 ± 2.2	0.0 ± 0.0	2.9 ± 2.7	0.0 ± 0.0								
			15:0_12:0(*sn*-2)_10:0					0.1 ± 0.2	0.0 ± 0.0	0.8 ± 1.2	0.0 ± 0.0								
		4:0_15:0_18:0	15:0_18:0(*sn*-2)_4:0			0.4 ± 0.8	0.0 ± 0.0	0.2 ± 0.2	0.0 ± 0.0	2.3 ± 4.0	0.0 ± 0.0								
			18:0_15:0(*sn*-2)_4:0			20.6 ± 7.9	0.0 ± 0.0	6.5 ± 5.7	0.0 ± 0.0	12.2 ± 10.9	0.1 ± 0.1								
			18:0_4:0(*sn*-2)_15:0			2.8 ± 3.9	0.0 ± 0.0	11.6 ± 11.9	0.0 ± 0.0	4.2 ± 7.3	0.0 ± 0.1								
		4:0_16:0_17:0	16:0_17:0(*sn*-2)_4:0			14.4 ± 11.4	0.0 ± 0.0	17.6 ± 19.9	0.0 ± 0.0	4.0 ± 5.8	0.0 ± 0.1								
			17:0_16:0(*sn*-2)_4:0			0.6 ± 1.1	0.0 ± 0.0	0.1 ± 0.1	0.0 ± 0.0	6.8 ± 6.0	0.1 ± 0.1								
			17:0_4:0(*sn*-2)_16:0			23.5 ± 9.2	0.0 ± 0.0	15.7 ± 16.8	0.0 ± 0.0	28.7 ± 14.6	0.2 ± 0.1								
		6:0_14:0_17:0	14:0_17:0(*sn*-2)_6:0			1.6 ± 2.1	0.0 ± 0.0	2.8 ± 5.2	0.0 ± 0.0	1.7 ± 2.2	0.0 ± 0.0								
			17:0_14:0(*sn*-2)_6:0			0.4 ± 0.7	0.0 ± 0.0	2.8 ± 5.5	0.0 ± 0.0	5.0 ± 7.0	0.0 ± 0.1								
			17:0_6:0(*sn*-2)_14:0			9.4 ± 7.4	0.0 ± 0.0	10.9 ± 8.1	0.0 ± 0.0	4.9 ± 5.6	0.0 ± 0.1								
		6:0_15:0_16:0	15:0_16:0(*sn*-2)_6:0			0.4 ± 0.7	0.0 ± 0.0	0.1 ± 0.2	0.0 ± 0.0	7.2 ± 9.8	0.1 ± 0.1								
			16:0_15:0(*sn*-2)_6:0			8.9 ± 12.7	0.0 ± 0.0	13.1 ± 16.0	0.0 ± 0.0	5.0 ± 8.2	0.0 ± 0.1								
			16:0_6:0(*sn*-2)_15:0			17.1 ± 11.5	0.0 ± 0.0	8.0 ± 15.4	0.0 ± 0.0	16.3 ± 26.6	0.1 ± 0.2								
		8:0_12:0_17:0	12:0_17:0(*sn*-2)_8:0							1.2 ± 1.7	0.0 ± 0.0								
			17:0_12:0(*sn*-2)_8:0							10.7 ± 15.1	0.1 ± 0.1								
			17:0_8:0(*sn*-2)_12:0							1.9 ± 2.7	0.0 ± 0.0								
	**37:1**			**nd**		**0.2 ± 0.2**		**nd**		**nd**		**nd**		**nd**		**nd**		**nd**	
		4:0_15:0_18:1	15:0_18:1(*sn*-2)_4:0			4.8 ± 7.4	0.0 ± 0.0												
			18:1_15:0(*sn*-2)_4:0			39.8 ± 24.0	0.1 ± 0.1												
			18:1_4:0(*sn*-2)_15:0			24.2 ± 29.6	0.0 ± 0.1												
		4:0_16:0_17:1	16:0_17:1(*sn*-2)_4:0			4.6 ± 7.2	0.0 ± 0.0												
			17:1_16:0(*sn*-2)_4:0			14.8 ± 14.2	0.0 ± 0.1												
			17:1_4:0(*sn*-2)_16:0			11.8 ± 11.7	0.0 ± 0.1												
**38**	**38:0**			**nd**		**7.1 ± 0.3[c]**		**6.7 ± 0.9[ab]**		**5.0 ± 0.1[de]**		**0.6 ± 0.0**		**nd**		**2.2 ± 0.1**		**nd**	
		10:0_10:0_18:0	10:0_18:0(*sn*-2)_10:0			1.3 ± 0.4	0.1 ± 0.0	7.3 ± 1.4	0.5 ± 0.2	3.5 ± 2.5	0.2 ± 0.1	0.0 ± 0.0	0.0 ± 0.0			1.1 ± 0.3	0.0 ± 0.0		
			18:0_10:0(*sn*-2)_10:0			1.3 ± 0.7	0.1 ± 0.1	1.7 ± 0.9	0.1 ± 0.1	1.3 ± 1.1	0.1 ± 0.1	9.1 ± 1.1	0.1 ± 0.0			0.6 ± 0.1	0.0 ± 0.0		
		10:0_12:0_16:0	12:0_16:0(*sn*-2)_10:0			0.6 ± 0.2	0.0 ± 0.0	2.8 ± 0.6	0.2 ± 0.1	1.1 ± 0.8	0.1 ± 0.0	0.0 ± 0.0	0.0 ± 0.0			15.2 ± 1.5	0.3 ± 0.0		
			16:0_10:0(*sn*-2)_12:0			0.5 ± 0.1	0.0 ± 0.0	4.7 ± 0.7	0.3 ± 0.1	2.0 ± 1.5	0.1 ± 0.1	38.1 ± 8.0	0.2 ± 0.1			13.3 ± 0.7	0.3 ± 0.0		
			16:0_12:0(*sn*-2)_10:0			0.6 ± 0.1	0.0 ± 0.0	8.0 ± 1.5	0.5 ± 0.2	2.7 ± 1.9	0.1 ± 0.1	0.0 ± 0.0	0.0 ± 0.0			15.8 ± 1.1	0.3 ± 0.0		
		10:0_14:0_14:0	14:0_10:0(*sn*-2)_14:0					2.6 ± 0.2	0.2 ± 0.0	1.0 ± 0.8	0.1 ± 0.0	14.0 ± 7.7	0.1 ± 0.1			3.0 ± 1.0	0.1 ± 0.0		
			14:0_14:0(*sn*-2)_10:0					3.7 ± 0.5	0.2 ± 0.1	1.4 ± 1.0	0.1 ± 0.1	0.0 ± 0.0	0.0 ± 0.0			5.4 ± 2.8	0.1 ± 0.1		
		12:0_12:0_14:0	12:0_14:0(*sn*-2)_12:0					1.3 ± 0.1	0.1 ± 0.0	0.7 ± 0.6	0.0 ± 0.0	0.0 ± 0.0	0.0 ± 0.0			6.0 ± 0.6	0.1 ± 0.0		
			14:0_12:0(*sn*-2)_12:0					1.4 ± 0.1	0.1 ± 0.0	0.7 ± 0.6	0.0 ± 0.0	41.1 ± 8.6	0.3 ± 0.1			4.1 ± 0.2	0.1 ± 0.0		
		4:0_16:0_18:0	16:0_18:0(*sn*-2)_4:0			17.3 ± 5.3	1.2 ± 0.4	11.7 ± 3.1	0.8 ± 0.3	9.8 ± 7.0	0.5 ± 0.4								
			18:0_16:0(*sn*-2)_4:0			14.5 ± 3.9	1.0 ± 0.3	4.0 ± 1.1	0.3 ± 0.1	5.0 ± 3.5	0.2 ± 0.2								
			18:0_4:0(*sn*-2)_16:0			4.1 ± 3.2	0.3 ± 0.2	1.9 ± 1.2	0.1 ± 0.1	2.1 ± 2.3	0.1 ± 0.1								
		6:0_14:0_18:0	14:0_18:0(*sn*-2)_6:0			3.8 ± 0.7	0.3 ± 0.1	1.5 ± 0.5	0.1 ± 0.0	1.5 ± 1.3	0.1 ± 0.1					0.9 ± 0.4	0.0 ± 0.0		
			18:0_14:0(*sn*-2)_6:0			8.4 ± 3.7	0.6 ± 0.3	2.6 ± 0.7	0.2 ± 0.1	2.7 ± 2.1	0.1 ± 0.1					0.6 ± 0.3	0.0 ± 0.0		
			18:0_6:0(*sn*-2)_14:0			1.3 ± 0.6	0.1 ± 0.0	1.5 ± 0.4	0.1 ± 0.0	1.4 ± 1.0	0.1 ± 0.1					0.6 ± 0.2	0.0 ± 0.0		
		6:0_16:0_16:0	16:0_16:0(*sn*-2)_6:0			28.5 ± 6.8	2.0 ± 0.6	16.8 ± 5.3	1.1 ± 0.5	8.5 ± 6.0	0.4 ± 0.3					3.4 ± 2.2	0.1 ± 0.0		
			16:0_6:0(*sn*-2)_16:0			7.4 ± 8.7	0.5 ± 0.6	9.5 ± 5.9	0.6 ± 0.5	6.4 ± 5.0	0.3 ± 0.3					3.4 ± 2.5	0.1 ± 0.1		
		8:0_12:0_18:0	12:0_18:0(*sn*-2)_8:0			1.3 ± 0.5	0.1 ± 0.0	0.9 ± 0.1	0.1 ± 0.0	0.5 ± 0.4	0.0 ± 0.0					1.3 ± 0.2	0.0 ± 0.0		
			18:0_12:0(*sn*-2)_8:0			1.9 ± 0.4	0.1 ± 0.0	1.5 ± 0.2	0.1 ± 0.0	1.3 ± 0.9	0.1 ± 0.0					0.7 ± 0.1	0.0 ± 0.0		
			18:0_8:0(*sn*-2)_12:0			0.9 ± 0.2	0.1 ± 0.0	1.2 ± 0.2	0.1 ± 0.0	1.0 ± 0.7	0.0 ± 0.0					0.7 ± 0.1	0.0 ± 0.0		
		8:0_14:0_16:0	14:0_16:0(*sn*-2)_8:0			2.3 ± 0.7	0.2 ± 0.1	3.1 ± 0.5	0.2 ± 0.1	1.2 ± 0.9	0.1 ± 0.0					8.3 ± 1.0	0.2 ± 0.0		
			16:0_14:0(*sn*-2)_8:0			3.2 ± 1.3	0.2 ± 0.1	6.0 ± 1.1	0.4 ± 0.1	2.3 ± 1.8	0.1 ± 0.1					9.8 ± 0.9	0.2 ± 0.0		
			16:0_8:0(*sn*-2)_14:0			1.1 ± 0.2	0.1 ± 0.0	4.1 ± 0.3	0.3 ± 0.1	1.8 ± 1.4	0.1 ± 0.1					5.8 ± 1.4	0.1 ± 0.0		
	**38:1**			**nd**		**7.2 ± 0.2[c]**		**5.2 ± 0.9[cd]**		**7.4 ± 0.0[a]**		**nd**		**2.6 ± 0.2**		**1.7 ± 0.1**		**nd**	
		10:0_10:0_18:1	10:0_18:1(*sn*-2)_10:0					11.8 ± 1.4	0.6 ± 0.2	7.0 ± 2.7	0.5 ± 0.2					2.1 ± 3.6	0.0 ± 0.1		
			18:1_10:0(*sn*-2)_10:0					5.0 ± 1.1	0.3 ± 0.1	2.8 ± 2.4	0.2 ± 0.2					12.7 ± 11.9	0.2 ± 0.2		

ACN	TG species	TG molecular species	TG regioisomer	Camel		Cow		Goat		Sheep		Human		Dog		Horse		Pig	
				in TG species	of total TGs	in TG species	of total TGs	in TG species	of total TGs	in TG species	of total TGs	in TG species	of total TGs	in TG species	of total TGs	in TG species	of total TGs	in TG species	of total TGs
		10:0_12:0_16:1	12:0_16:1(*sn*-2)_10:0													2.7 ± 4.6	0.0 ± 0.1		
			16:1_10:0(*sn*-2)_12:0													8.2 ± 8.2	0.1 ± 0.1		
			16:1_12:0(*sn*-2)_10:0													18.8 ± 20.2	0.3 ± 0.4		
		4:0_16:0_18:1	16:0_18:1(*sn*-2)_4:0			43.1 ± 1.1	3.1 ± 0.2	27.2 ± 2.7	1.4 ± 0.4	41.7 ± 10.4	3.1 ± 0.8			14.1 ± 15.5	0.4 ± 0.4	0.8 ± 1.4	0.0 ± 0.0		
			18:1_16:0(*sn*-2)_4:0			44.7 ± 8.6	3.2 ± 0.7	21.5 ± 7.1	1.1 ± 0.6	9.7 ± 8.6	0.7 ± 0.6			67.9 ± 6.4	1.7 ± 0.3	30.0 ± 4.5	0.5 ± 0.1		
			18:1_4:0(*sn*-2)_16:0			0.3 ± 0.6	0.0 ± 0.0	8.5 ± 7.1	0.4 ± 0.4	13.0 ± 11.4	1.0 ± 0.8			18.0 ± 18.0	0.5 ± 0.5	0.4 ± 0.7	0.0 ± 0.0		
		4:0_16:1_18:0	16:1_18:0(*sn*-2)_4:0							0.2 ± 0.2	0.0 ± 0.0								
			18:0_16:1(*sn*-2)_4:0							2.3 ± 0.7	0.2 ± 0.1								
			18:0_4:0(*sn*-2)_16:1							0.3 ± 0.2	0.0 ± 0.0								
		6:0_14:0_18:1	14:0_18:1(*sn*-2)_6:0			3.8 ± 2.3	0.3 ± 0.2	4.1 ± 3.0	0.2 ± 0.2	1.5 ± 1.9	0.1 ± 0.1								
			18:1_14:0(*sn*-2)_6:0			5.2 ± 9.1	0.4 ± 0.7	13.0 ± 3.0	0.7 ± 0.3	12.5 ± 4.1	0.9 ± 0.3								
			18:1_6:0(*sn*-2)_14:0			2.9 ± 4.9	0.2 ± 0.4	1.2 ± 1.1	0.1 ± 0.1	1.6 ± 2.1	0.1 ± 0.2								
		6:0_16:0_16:1	16:0_16:1(*sn*-2)_6:0							1.6 ± 0.7	0.1 ± 0.1								
			16:0_6:0(*sn*-2)_16:1							0.4 ± 0.3	0.0 ± 0.0								
			16:1_16:0(*sn*-2)_6:0							0.3 ± 0.2	0.0 ± 0.0								
		8:0_12:0_18:1	12:0_18:1(*sn*-2)_8:0					3.0 ± 1.2	0.2 ± 0.1	0.6 ± 0.6	0.0 ± 0.0								
			18:1_12:0(*sn*-2)_8:0					2.2 ± 2.5	0.1 ± 0.2	2.2 ± 2.0	0.2 ± 0.1								
			18:1_8:0(*sn*-2)_12:0					2.5 ± 0.4	0.1 ± 0.0	3.4 ± 2.3	0.2 ± 0.2								
		8:0_14:0_16:1	14:0_16:1(*sn*-2)_8:0													0.8 ± 1.4	0.0 ± 0.0		
			16:1_14:0(*sn*-2)_8:0													16.1 ± 10.8	0.3 ± 0.2		
			16:1_8:0(*sn*-2)_14:0													7.4 ± 11.5	0.1 ± 0.2		
	**38:2**			**nd**		**0.4 ± 0.1**		**0.2 ± 0.0**		**1.6 ± 0.0**		**nd**		**1.0 ± 0.2**		**0.4 ± 0.1**		**nd**	
		10:0_10:0_18:2	10:0_18:2(*sn*-2)_10:0							6.5 ± 3.7	0.1 ± 0.1					3.4 ± 6.8	0.0 ± 0.0		
			18:2_10:0(*sn*-2)_10:0							1.0 ± 1.7	0.0 ± 0.0					50.1 ± 36.4	0.2 ± 0.2		
		4:0_16:0_18:2	16:0_18:2(*sn*-2)_4:0			5.1 ± 5.9	0.0 ± 0.0	4.0 ± 7.8	0.0 ± 0.0	0.9 ± 1.5	0.0 ± 0.0			0.0 ± 0.0	0.0 ± 0.0				
			18:2_16:0(*sn*-2)_4:0			25.1 ± 18.0	0.1 ± 0.1	65.4 ± 37.9	0.1 ± 0.1	0.0 ± 0.0	0.0 ± 0.0			63.1 ± 2.6	0.6 ± 0.1				
			18:2_4:0(*sn*-2)_16:0			20.8 ± 16.6	0.1 ± 0.1	15.3 ± 30.2	0.0 ± 0.1	47.5 ± 2.9	0.8 ± 0.0			0.0 ± 0.0	0.0 ± 0.0				
		4:0_16:1_18:1	16:1_18:1(*sn*-2)_4:0			0.1 ± 0.1	0.0 ± 0.0	0.0 ± 0.0	0.0 ± 0.0	0.0 ± 0.0	0.0 ± 0.0			21.8 ± 13.2	0.2 ± 0.2	22.0 ± 6.1	0.1 ± 0.1		
			18:1_16:1(*sn*-2)_4:0			35.3 ± 6.3	0.1 ± 0.1	30.8 ± 5.4	0.1 ± 0.0	33.1 ± 1.0	0.5 ± 0.0			0.0 ± 0.0	0.0 ± 0.0	21.8 ± 10.5	0.1 ± 0.1		
			18:1_4:0(*sn*-2)_16:1			0.1 ± 0.2	0.0 ± 0.0	0.0 ± 0.0	0.0 ± 0.0	0.1 ± 0.1	0.0 ± 0.0			15.1 ± 13.2	0.1 ± 0.2	0.0 ± 0.0	0.0 ± 0.0		
		6:0_14:0_18:2	14:0_18:2(*sn*-2)_6:0			1.5 ± 2.2	0.0 ± 0.0			1.3 ± 2.0	0.0 ± 0.0								
			18:2_14:0(*sn*-2)_6:0			3.5 ± 3.8	0.0 ± 0.0			5.6 ± 5.4	0.1 ± 0.1								
			18:2_6:0(*sn*-2)_14:0			0.3 ± 0.6	0.0 ± 0.0			4.1 ± 5.2	0.1 ± 0.1								
		6:0_14:1_18:1	14:1_18:1(*sn*-2)_6:0			0.4 ± 0.9	0.0 ± 0.0												
			18:1_14:1(*sn*-2)_6:0			6.7 ± 4.4	0.0 ± 0.0												
			18:1_6:0(*sn*-2)_14:1			1.1 ± 2.3	0.0 ± 0.0												
		8:0_12:0_18:2	12:0_18:2(*sn*-2)_8:0													0.1 ± 0.1	0.0 ± 0.0		
			18:2_12:0(*sn*-2)_8:0													43.7 ± 22.3	0.2 ± 0.1		
			18:2_8:0(*sn*-2)_12:0													5.3 ± 7.0	0.0 ± 0.0		
**39**	**39:0**			**nd**		**nd**		**0.7 ± 0.2**		**nd**		**nd**		**nd**		**nd**		**nd**	
		10:0_14:0_15:0	14:0_15:0(*sn*-2)_10:0					21.6 ± 7.3	0.1 ± 0.1										
			15:0_10:0(*sn*-2)_14:0					0.4 ± 0.9	0.0 ± 0.0										
			15:0_14:0(*sn*-2)_10:0					4.4 ± 5.6	0.0 ± 0.0										
		12:0_12:0_15:0	12:0_15:0(*sn*-2)_12:0					5.0 ± 2.2	0.0 ± 0.0										
			15:0_12:0(*sn*-2)_12:0					0.0 ± 0.0	0.0 ± 0.0										
		6:0_15:0_18:0	15:0_18:0(*sn*-2)_6:0					0.0 ± 0.0	0.0 ± 0.0										
			18:0_15:0(*sn*-2)_6:0					16.2 ± 5.0	0.1 ± 0.1										
			18:0_6:0(*sn*-2)_15:0					2.9 ± 3.6	0.0 ± 0.0										
		6:0_16:0_17:0	16:0_17:0(*sn*-2)_6:0					2.2 ± 4.4	0.0 ± 0.0										
			17:0_16:0(*sn*-2)_6:0					0.0 ± 0.0	0.0 ± 0.0										
			17:0_6:0(*sn*-2)_16:0					24.6 ± 4.6	0.2 ± 0.1										
		8:0_14:0_17:0	14:0_17:0(*sn*-2)_8:0					0.0 ± 0.0	0.0 ± 0.0										
			17:0_14:0(*sn*-2)_8:0					3.9 ± 5.1	0.0 ± 0.0										
			17:0_8:0(*sn*-2)_14:0					4.2 ± 5.3	0.0 ± 0.0										
		8:0_15:0_16:0	15:0_16:0(*sn*-2)_8:0					0.0 ± 0.0	0.0 ± 0.0										
			16:0_15:0(*sn*-2)_8:0					17.1 ± 3.2	0.1 ± 0.1										
			16:0_8:0(*sn*-2)_15:0					0.0 ± 0.0	0.0 ± 0.0										
	**39:1**			**nd**		**nd**		**nd**		**0.3 ± 0.1**		**nd**		**nd**		**nd**		**nd**	
		4:0_17:0_18:1	17:0_18:1(*sn*-2)_4:0							7.7 ± 11.0	0.0 ± 0.0								
			18:1_17:0(*sn*-2)_4:0							54.9 ± 42.4	0.1 ± 0.1								
			18:1_4:0(*sn*-2)_17:0							20.9 ± 26.6	0.1 ± 0.1								

ACN	TG species	TG molecular species	TG regioisomer	Camel		Cow		Goat		Sheep		Human		Dog		Horse		Pig	
				in TG species	of total TGs	in TG species	of total TGs	in TG species	of total TGs	in TG species	of total TGs	in TG species	of total TGs	in TG species	of total TGs	in TG species	of total TGs	in TG species	of total TGs
		6:0_15:0_18:1	15:0_18:1(*sn*-2)_6:0							17.2 ± 17.1	0.0 ± 0.1								
			18:1_15:0(*sn*-2)_6:0							11.4 ± 16.1	0.0 ± 0.0								
			18:1_6:0(*sn*-2)_15:0							4.5 ± 6.4	0.0 ± 0.0								
**40**	**40:0**			**nd**		**4.0 ± 0.6**		**7.4 ± 0.2[a]**		**3.9 ± 0.1**		**1.3 ± 0.2**		**nd**		**3.2 ± 0.1**		**nd**	
		10:0_12:0_18:0	12:0_18:0(*sn*-2)_10:0			0.7 ± 0.3	0.0 ± 0.0			1.1 ± 0.5	0.0 ± 0.0					0.9 ± 0.4	0.0 ± 0.0		
			18:0_10:0(*sn*-2)_12:0			1.2 ± 0.5	0.0 ± 0.0			1.4 ± 0.8	0.1 ± 0.0					1.1 ± 0.4	0.0 ± 0.0		
			18:0_12:0(*sn*-2)_10:0			1.4 ± 0.8	0.1 ± 0.0			2.8 ± 1.8	0.1 ± 0.1					1.1 ± 0.4	0.0 ± 0.0		
		10:0_14:0_16:0	14:0_16:0(*sn*-2)_10:0			2.5 ± 1.4	0.1 ± 0.1	7.1 ± 2.2	0.5 ± 0.2	4.5 ± 2.5	0.2 ± 0.1					9.6 ± 5.6	0.3 ± 0.2		
			16:0_10:0(*sn*-2)_14:0			6.4 ± 4.5	0.3 ± 0.2	2.8 ± 3.8	0.2 ± 0.3	2.7 ± 1.5	0.1 ± 0.1					8.0 ± 6.6	0.3 ± 0.2		
			16:0_14:0(*sn*-2)_10:0			16.4 ± 5.7	0.6 ± 0.3	39.6 ± 5.3	2.9 ± 0.5	24.5 ± 6.2	0.9 ± 0.3					23.0 ± 7.9	0.7 ± 0.3		
		12:0_12:0_16:0	12:0_16:0(*sn*-2)_12:0			2.7 ± 1.2	0.1 ± 0.1	5.9 ± 1.8	0.4 ± 0.1	4.8 ± 1.4	0.2 ± 0.1	1.1 ± 1.2	0.0 ± 0.0			12.6 ± 3.7	0.4 ± 0.1		
			16:0_12:0(*sn*-2)_12:0			1.0 ± 0.7	0.0 ± 0.0	0.8 ± 0.9	0.1 ± 0.1	1.2 ± 1.0	0.0 ± 0.0	56.6 ± 5.9	0.7 ± 0.2			4.2 ± 4.6	0.1 ± 0.2		
		12:0_14:0_14:0	14:0_12:0(*sn*-2)_14:0			1.6 ± 0.3	0.1 ± 0.0			1.0 ± 0.3	0.0 ± 0.0	38.9 ± 2.0	0.5 ± 0.1			3.2 ± 2.1	0.1 ± 0.1		
			14:0_14:0(*sn*-2)_12:0			2.1 ± 0.8	0.1 ± 0.0			1.7 ± 0.8	0.1 ± 0.0	3.5 ± 4.1	0.0 ± 0.1			5.5 ± 1.0	0.2 ± 0.0		
		4:0_18:0_18:0	18:0_18:0(*sn*-2)_4:0			11.2 ± 1.0	0.4 ± 0.1	6.7 ± 0.8	0.5 ± 0.1	14.6 ± 1.8	0.6 ± 0.1								
			18:0_4:0(*sn*-2)_18:0			0.5 ± 0.4	0.0 ± 0.0	0.2 ± 0.2	0.0 ± 0.0	0.4 ± 0.3	0.0 ± 0.0								
		6:0_16:0_18:0	16:0_18:0(*sn*-2)_6:0			19.9 ± 3.8	0.8 ± 0.3	11.1 ± 2.2	0.8 ± 0.2	17.6 ± 5.2	0.7 ± 0.2								
			18:0_16:0(*sn*-2)_6:0			6.0 ± 3.1	0.2 ± 0.2	1.3 ± 1.0	0.1 ± 0.1	2.6 ± 2.1	0.1 ± 0.1								
			18:0_6:0(*sn*-2)_16:0			0.6 ± 0.5	0.0 ± 0.0	0.3 ± 0.2	0.0 ± 0.0	0.7 ± 0.6	0.0 ± 0.0								
		8:0_14:0_18:0	14:0_18:0(*sn*-2)_8:0			1.1 ± 0.4	0.0 ± 0.0	4.0 ± 3.3	0.3 ± 0.3	1.7 ± 0.5	0.1 ± 0.0					3.6 ± 4.3	0.1 ± 0.1		
			18:0_14:0(*sn*-2)_8:0			4.2 ± 3.0	0.2 ± 0.1	1.3 ± 0.7	0.1 ± 0.1	3.4 ± 0.9	0.1 ± 0.0					1.3 ± 1.0	0.0 ± 0.0		
			18:0_8:0(*sn*-2)_14:0			1.0 ± 0.7	0.0 ± 0.0	0.4 ± 0.3	0.0 ± 0.0	0.7 ± 0.5	0.0 ± 0.0					0.9 ± 0.7	0.0 ± 0.0		
		8:0_16:0_16:0	16:0_16:0(*sn*-2)_8:0			16.9 ± 4.2	0.7 ± 0.3	18.1 ± 2.2	1.3 ± 0.2	12.9 ± 1.8	0.5 ± 0.1					20.2 ± 6.1	0.7 ± 0.2		
			16:0_8:0(*sn*-2)_16:0			3.3 ± 5.4	0.1 ± 0.2	0.4 ± 0.4	0.0 ± 0.0	0.9 ± 0.8	0.0 ± 0.0					5.9 ± 7.6	0.2 ± 0.3		
	**40:1**			**nd**		**4.1 ± 0.1**		**4.6 ± 0.5[d]**		**5.2 ± 0.0[d]**		**nd**		**0.3 ± 0.1**		**3.7 ± 0.3[de]**		**nd**	
		10:0_12:0_18:1	12:0_18:1(*sn*-2)_10:0			0.6 ± 0.2	0.0 ± 0.0	4.1 ± 0.6	0.2 ± 0.0	3.3 ± 1.6	0.2 ± 0.1					5.9 ± 4.5	0.2 ± 0.2		
			18:1_10:0(*sn*-2)_12:0			1.0 ± 0.3	0.0 ± 0.0	2.1 ± 0.8	0.1 ± 0.0	5.5 ± 3.0	0.3 ± 0.2					4.7 ± 3.1	0.2 ± 0.1		
			18:1_12:0(*sn*-2)_10:0			2.3 ± 1.3	0.1 ± 0.1	11.9 ± 3.0	0.5 ± 0.2	5.0 ± 2.1	0.3 ± 0.1					10.1 ± 5.8	0.4 ± 0.2		
		10:0_14:0_16:1	14:0_16:1(*sn*-2)_10:0			0.6 ± 0.1	0.0 ± 0.0	1.6 ± 1.1	0.1 ± 0.1							3.1 ± 3.5	0.1 ± 0.1		
			16:1_10:0(*sn*-2)_14:0			0.5 ± 0.1	0.0 ± 0.0	0.9 ± 0.6	0.0 ± 0.0							3.8 ± 3.9	0.1 ± 0.2		
			16:1_14:0(*sn*-2)_10:0			0.4 ± 0.1	0.0 ± 0.0	1.1 ± 0.5	0.0 ± 0.0							10.6 ± 1.9	0.4 ± 0.1		
		10:0_14:1_16:0	14:1_16:0(*sn*-2)_10:0			0.4 ± 0.1	0.0 ± 0.0									2.9 ± 2.8	0.1 ± 0.1		
			16:0_10:0(*sn*-2)_14:1			0.4 ± 0.1	0.0 ± 0.0									0.7 ± 0.6	0.0 ± 0.0		
			16:0_14:1(*sn*-2)_10:0			0.7 ± 0.1	0.0 ± 0.0									0.7 ± 0.7	0.0 ± 0.0		
		12:0_12:0_16:1	12:0_16:1(*sn*-2)_12:0													1.1 ± 1.4	0.0 ± 0.1		
			16:1_12:0(*sn*-2)_12:0													7.0 ± 3.3	0.3 ± 0.1		
		4:0_18:0_18:1	18:0_18:1(*sn*-2)_4:0			7.5 ± 1.4	0.3 ± 0.1	3.8 ± 1.2	0.2 ± 0.1	18.0 ± 1.9	0.9 ± 0.1			11.3 ± 16.0	0.0 ± 0.1				
			18:0_4:0(*sn*-2)_18:1			1.0 ± 0.5	0.0 ± 0.0	2.1 ± 0.7	0.1 ± 0.0	0.6 ± 0.8	0.0 ± 0.0			0.0 ± 0.0	0.0 ± 0.0				
			18:1_18:0(*sn*-2)_4:0			22.9 ± 3.6	0.9 ± 0.2	10.0 ± 1.3	0.5 ± 0.1	17.4 ± 2.1	0.9 ± 0.1			17.8 ± 18.0	0.1 ± 0.1				
		6:0_16:0_18:1	16:0_18:1(*sn*-2)_6:0			23.6 ± 7.0	1.0 ± 0.3	14.8 ± 2.1	0.7 ± 0.2	27.1 ± 5.0	1.4 ± 0.3			15.4 ± 24.6	0.0 ± 0.1	1.0 ± 1.2	0.0 ± 0.0		
			18:1_16:0(*sn*-2)_6:0			17.0 ± 7.4	0.7 ± 0.3	13.4 ± 3.7	0.6 ± 0.2	2.9 ± 4.7	0.1 ± 0.2			55.0 ± 42.3	0.2 ± 0.2	5.6 ± 7.5	0.2 ± 0.3		
			18:1_6:0(*sn*-2)_16:0			9.5 ± 6.9	0.4 ± 0.3	13.9 ± 0.7	0.6 ± 0.1	8.1 ± 5.0	0.4 ± 0.3			15.1 ± 29.8	0.0 ± 0.1	4.8 ± 4.6	0.2 ± 0.2		
		6:0_16:1_18:0	16:1_18:0(*sn*-2)_6:0			0.3 ± 0.1	0.0 ± 0.0	0.6 ± 0.1	0.0 ± 0.0										
			18:0_16:1(*sn*-2)_6:0			0.5 ± 0.0	0.0 ± 0.0	0.9 ± 0.3	0.0 ± 0.0										
			18:0_6:0(*sn*-2)_16:1			0.3 ± 0.1	0.0 ± 0.0	0.6 ± 0.1	0.0 ± 0.0										
		8:0_14:0_18:1	14:0_18:1(*sn*-2)_8:0			1.6 ± 1.0	0.1 ± 0.0	3.8 ± 1.4	0.2 ± 0.1	1.6 ± 1.2	0.1 ± 0.1					2.0 ± 2.2	0.1 ± 0.1		
			18:1_14:0(*sn*-2)_8:0			4.3 ± 2.1	0.2 ± 0.1	7.6 ± 2.5	0.4 ± 0.2	2.5 ± 3.5	0.1 ± 0.2					7.5 ± 6.9	0.3 ± 0.3		
			18:1_8:0(*sn*-2)_14:0			2.2 ± 1.1	0.1 ± 0.0	2.5 ± 1.4	0.1 ± 0.1	7.9 ± 3.5	0.4 ± 0.2					7.8 ± 9.8	0.3 ± 0.4		
		8:0_14:1_18:0	14:1_18:0(*sn*-2)_8:0			0.3 ± 0.0	0.0 ± 0.0												
			18:0_14:1(*sn*-2)_8:0			0.5 ± 0.1	0.0 ± 0.0												
			18:0_8:0(*sn*-2)_14:1			0.3 ± 0.0	0.0 ± 0.0												
		8:0_16:0_16:1	16:0_16:1(*sn*-2)_8:0			1.1 ± 0.4	0.0 ± 0.0	2.3 ± 0.4	0.1 ± 0.0							2.9 ± 3.3	0.1 ± 0.1		
			16:0_8:0(*sn*-2)_16:1			0.6 ± 0.3	0.0 ± 0.0	1.1 ± 0.3	0.0 ± 0.0							8.4 ± 7.2	0.3 ± 0.3		
			16:1_16:0(*sn*-2)_8:0			0.5 ± 0.1	0.0 ± 0.0	1.0 ± 0.1	0.0 ± 0.0							10.3 ± 7.5	0.4 ± 0.3		

ACN	TG species	TG molecular species	TG regioisomer	Camel		Cow		Goat		Sheep		Human		Dog		Horse		Pig	
				in TG species	of total TGs	in TG species	of total TGs	in TG species	of total TGs	in TG species	of total TGs	in TG species	of total TGs	in TG species	of total TGs	in TG species	of total TGs	in TG species	of total TGs
	**40:2**			**nd**		**1.2 ± 0.1**		**1.5 ± 0.3**		**3.1 ± 0.2**		**nd**		**0.4 ± 0.0**		**nd**		**nd**	
		4:0_18:0_18:2	18:0_18:2(*sn*-2)_4:0			2.3 ± 2.1	0.0 ± 0.0												
			18:0_4:0(*sn*-2)_18:2			0.0 ± 0.0	0.0 ± 0.0												
			18:2_18:0(*sn*-2)_4:0			13.7 ± 1.0	0.2 ± 0.0												
		4:0_18:1_18:1	18:1_18:1(*sn*-2)_4:0			79.8 ± 13.5	1.0 ± 0.2	100.0 ± 0.0	1.5 ± 0.3	91.3 ± 7.6	2.9 ± 0.4			99.8 ± 0.1	0.4 ± 0.0				
			18:1_4:0(*sn*-2)_18:1			0.0 ± 0.0	0.0 ± 0.0	0.0 ± 0.0	0.0 ± 0.0	0.0 ± 0.1	0.0 ± 0.0			0.2 ± 0.1	0.0 ± 0.0				
		6:0_16:0_18:2	16:0_18:2(*sn*-2)_6:0			7.6 ± 6.6	0.1 ± 0.1												
			18:2_16:0(*sn*-2)_6:0			3.3 ± 5.8	0.0 ± 0.1												
			18:2_6:0(*sn*-2)_16:0			0.0 ± 0.0	0.0 ± 0.0												
		6:0_16:1_18:1	16:1_18:1(*sn*-2)_6:0							8.6 ± 7.5	0.3 ± 0.2								
			18:1_16:1(*sn*-2)_6:0							0.0 ± 0.1	0.0 ± 0.0								
			18:1_6:0(*sn*-2)_16:1							0.0 ± 0.1	0.0 ± 0.0								
	**40:3**			**nd**		**0.2 ± 0.2**		**nd**		**0.3 ± 0.0**		**nd**		**0.2 ± 0.1**		**0.7 ± 0.1**		**nd**	
		10:0_12:0_18:3	12:0_18:3(*sn*-2)_10:0													16.5 ± 19.9	0.1 ± 0.2		
			18:3_10:0(*sn*-2)_12:0													20.6 ± 19.4	0.2 ± 0.2		
			18:3_12:0(*sn*-2)_10:0													12.6 ± 4.2	0.1 ± 0.0		
		4:0_16:0_20:3	16:0_20:3(*sn*-2)_4:0							12.6 ± 6.2	0.0 ± 0.0								
			20:3_16:0(*sn*-2)_4:0							0.5 ± 0.7	0.0 ± 0.0								
			20:3_4:0(*sn*-2)_16:0							0.0 ± 0.0	0.0 ± 0.0								
		4:0_18:0_18:3	18:0_18:3(*sn*-2)_4:0			0.1 ± 0.1	0.0 ± 0.0			0.0 ± 0.0	0.0 ± 0.0								
			18:0_4:0(*sn*-2)_18:3			0.1 ± 0.2	0.0 ± 0.0			0.0 ± 0.0	0.0 ± 0.0								
			18:3_18:0(*sn*-2)_4:0			25.3 ± 5.6	0.1 ± 0.1			25.8 ± 3.4	0.1 ± 0.0								
		4:0_18:1_18:2	18:1_18:2(*sn*-2)_4:0			39.5 ± 15.4	0.1 ± 0.1			32.2 ± 23.0	0.1 ± 0.1			8.6 ± 14.9	0.0 ± 0.0				
			18:1_4:0(*sn*-2)_18:2			2.8 ± 5.5	0.0 ± 0.0			12.1 ± 21.4	0.0 ± 0.1			0.2 ± 0.3	0.0 ± 0.0				
			18:2_18:1(*sn*-2)_4:0			44.9 ± 14.8	0.1 ± 0.1			36.2 ± 13.0	0.1 ± 0.0			91.1 ± 14.8	0.2 ± 0.2				
		6:0_16:0_18:3	16:0_18:3(*sn*-2)_6:0													3.0 ± 5.9	0.0 ± 0.0		
			18:3_16:0(*sn*-2)_6:0													8.9 ± 10.1	0.1 ± 0.1		
			18:3_6:0(*sn*-2)_16:0													8.6 ± 11.7	0.1 ± 0.1		
		8:0_14:0_18:3	14:0_18:3(*sn*-2)_8:0													14.6 ± 11.9	0.1 ± 0.1		
			18:3_14:0(*sn*-2)_8:0													10.8 ± 2.4	0.1 ± 0.0		
			18:3_8:0(*sn*-2)_14:0													4.3 ± 6.5	0.0 ± 0.1		
**42**	**42:0**			**0.8 ± 0.1**		**3.4 ± 0.2**		**7.3 ± 0.1[a]**		**3.0 ± 0.3**		**1.5 ± 0.1**		**nd**		**2.9 ± 0.2**		**nd**	
		10:0_14:0_18:0	14:0_18:0(*sn*-2)_10:0			2.0 ± 0.8	0.1 ± 0.0	1.3 ± 0.9	0.1 ± 0.1	2.2 ± 0.6	0.1 ± 0.0	3.3 ± 1.3	0.1 ± 0.0			4.4 ± 4.4	0.1 ± 0.1		
			18:0_10:0(*sn*-2)_14:0			2.2 ± 1.9	0.1 ± 0.1	0.8 ± 0.9	0.1 ± 0.1	4.5 ± 1.9	0.1 ± 0.1	7.1 ± 2.9	0.1 ± 0.1			0.5 ± 0.7	0.0 ± 0.0		
			18:0_14:0(*sn*-2)_10:0			7.0 ± 1.6	0.2 ± 0.1	7.6 ± 2.6	0.6 ± 0.2	8.0 ± 3.3	0.2 ± 0.1	3.4 ± 1.1	0.1 ± 0.0			0.6 ± 0.7	0.0 ± 0.0		
		10:0_16:0_16:0	16:0_10:0(*sn*-2)_16:0			4.1 ± 4.3	0.1 ± 0.2	0.7 ± 0.9	0.0 ± 0.1	8.3 ± 4.6	0.2 ± 0.2	15.0 ± 4.8	0.2 ± 0.1			4.5 ± 7.5	0.1 ± 0.2		
			16:0_16:0(*sn*-2)_10:0			39.4 ± 3.1	1.3 ± 0.2	54.5 ± 1.9	4.0 ± 0.2	34.7 ± 5.8	1.0 ± 0.3	6.4 ± 3.6	0.1 ± 0.1			50.7 ± 9.9	1.5 ± 0.4		
		12:0_12:0_18:0	12:0_18:0(*sn*-2)_12:0	0.9 ± 1.8	0.0 ± 0.0	2.0 ± 0.5	0.1 ± 0.0	2.0 ± 1.4	0.1 ± 0.1	1.8 ± 0.5	0.1 ± 0.0	7.0 ± 4.2	0.1 ± 0.1						
			18:0_12:0(*sn*-2)_12:0	28.3 ± 2.7	0.2 ± 0.0	1.4 ± 0.9	0.0 ± 0.0	0.2 ± 0.2	0.0 ± 0.0	1.2 ± 0.3	0.0 ± 0.0	12.9 ± 8.6	0.2 ± 0.1						
		12:0_14:0_16:0	14:0_16:0(*sn*-2)_12:0	1.5 ± 2.9	0.0 ± 0.0	4.1 ± 1.2	0.1 ± 0.0	5.1 ± 2.0	0.4 ± 0.1	2.6 ± 0.4	0.1 ± 0.0	9.6 ± 4.4	0.1 ± 0.1			15.7 ± 16.1	0.5 ± 0.5		
			16:0_12:0(*sn*-2)_14:0	97.4 ± 32.1	0.8 ± 0.3	6.2 ± 2.8	0.2 ± 0.1	3.4 ± 4.1	0.2 ± 0.3	5.4 ± 1.2	0.2 ± 0.1	13.8 ± 0.7	0.2 ± 0.0			9.2 ± 10.8	0.3 ± 0.3		
			16:0_14:0(*sn*-2)_12:0	5.6 ± 11.2	0.0 ± 0.1	11.7 ± 5.6	0.4 ± 0.2	6.6 ± 1.5	0.5 ± 0.1	4.8 ± 0.7	0.1 ± 0.0	11.2 ± 5.6	0.2 ± 0.1			7.0 ± 5.2	0.2 ± 0.2		
		14:0_14:0_14:0	14:0_14:0(*sn*-2)_14:0	66.3 ± 17.7	0.5 ± 0.2	3.2 ± 1.0	0.1 ± 0.0	3.4 ± 1.1	0.2 ± 0.1	2.3 ± 0.5	0.1 ± 0.0	10.2 ± 3.8	0.2 ± 0.1			4.7 ± 0.2	0.1 ± 0.0		
		6:0_18:0_18:0	18:0_18:0(*sn*-2)_6:0			4.8 ± 1.0	0.2 ± 0.0	5.1 ± 1.1	0.4 ± 0.1	8.5 ± 2.5	0.3 ± 0.1								
			18:0_6:0(*sn*-2)_18:0			0.9 ± 0.8	0.0 ± 0.0	0.3 ± 0.3	0.0 ± 0.0	1.8 ± 0.4	0.1 ± 0.0								
		8:0_16:0_18:0	16:0_18:0(*sn*-2)_8:0			5.9 ± 3.1	0.2 ± 0.1	5.5 ± 2.2	0.4 ± 0.2	5.4 ± 1.4	0.2 ± 0.1					3.7 ± 1.7	0.1 ± 0.1		
			18:0_16:0(*sn*-2)_8:0			4.2 ± 1.9	0.1 ± 0.1	2.9 ± 2.6	0.2 ± 0.2	5.4 ± 0.8	0.2 ± 0.0					0.9 ± 0.7	0.0 ± 0.0		
			18:0_8:0(*sn*-2)_16:0			1.7 ± 1.7	0.1 ± 0.1	0.7 ± 0.8	0.0 ± 0.1	3.1 ± 0.6	0.1 ± 0.0					0.7 ± 0.7	0.0 ± 0.0		
	**42:1**			**nd**		**2.4 ± 0.2**		**4.9 ± 0.2[d]**		**4.9 ± 0.2[de]**		**1.9 ± 0.3**		**nd**		**5.8 ± 0.1[b]**		**nd**	
		10:0_14:0_18:1	14:0_18:1(*sn*-2)_10:0			10.1 ± 8.0	0.2 ± 0.2	4.0 ± 6.0	0.2 ± 0.3	9.7 ± 1.2	0.5 ± 0.1	0.0 ± 0.0	0.0 ± 0.0			3.6 ± 4.3	0.2 ± 0.3		
			18:1_10:0(*sn*-2)_14:0			4.8 ± 4.6	0.1 ± 0.1	7.1 ± 4.6	0.3 ± 0.2	12.9 ± 5.2	0.6 ± 0.3	35.9 ± 4.0	0.7 ± 0.2			6.5 ± 6.2	0.4 ± 0.4		
			18:1_14:0(*sn*-2)_10:0			10.8 ± 7.2	0.3 ± 0.2	31.9 ± 5.7	1.6 ± 0.3	10.1 ± 6.3	0.5 ± 0.3	2.9 ± 2.8	0.1 ± 0.1			10.7 ± 1.4	0.6 ± 0.1		
		10:0_14:1_18:0	14:1_18:0(*sn*-2)_10:0			1.4 ± 1.3	0.0 ± 0.0												
			18:0_10:0(*sn*-2)_14:1			0.7 ± 0.6	0.0 ± 0.0												
			18:0_14:1(*sn*-2)_10:0			1.2 ± 1.2	0.0 ± 0.0												
		10:0_16:0_16:1	16:0_10:0(*sn*-2)_16:1			1.0 ± 0.9	0.0 ± 0.0	3.9 ± 5.8	0.2 ± 0.3							17.3 ± 14.9	1.0 ± 0.9		
			16:0_16:1(*sn*-2)_10:0			2.8 ± 2.7	0.1 ± 0.1	2.8 ± 2.8	0.1 ± 0.1							3.0 ± 4.4	0.2 ± 0.3		
			16:1_16:0(*sn*-2)_10:0			1.1 ± 1.0	0.0 ± 0.0	1.1 ± 1.2	0.1 ± 0.1							8.2 ± 6.5	0.5 ± 0.4		
		12:0_12:0_18:1	12:0_18:1(*sn*-2)_12:0			1.7 ± 1.4	0.0 ± 0.0	0.5 ± 0.9	0.0 ± 0.0	2.0 ± 0.5	0.1 ± 0.0	0.0 ± 0.0	0.0 ± 0.0			1.1 ± 1.6	0.1 ± 0.1		
			18:1_12:0(*sn*-2)_12:0			1.0 ± 1.1	0.0 ± 0.0	5.6 ± 1.9	0.3 ± 0.1	1.3 ± 0.4	0.1 ± 0.0	61.2 ± 4.5	1.1 ± 0.3			9.3 ± 5.4	0.5 ± 0.3		
		12:0_14:0_16:1	14:0_16:1(*sn*-2)_12:0			2.2 ± 2.7	0.1 ± 0.1									1.7 ± 1.9	0.1 ± 0.1		
			16:1_12:0(*sn*-2)_14:0			0.5 ± 0.4	0.0 ± 0.0									6.3 ± 5.5	0.4 ± 0.3		
			16:1_14:0(*sn*-2)_12:0			0.4 ± 0.4	0.0 ± 0.0									2.5 ± 2.3	0.1 ± 0.1		

ACN	TG species	TG molecular species	TG regioisomer	Camel		Cow		Goat		Sheep		Human		Dog		Horse		Pig	
				in TG species	of total TGs	in TG species	of total TGs	in TG species	of total TGs	in TG species	of total TGs	in TG species	of total TGs	in TG species	of total TGs	in TG species	of total TGs	in TG species	of total TGs
		12:0_14:1_16:0	14:1_16:0(*sn*-2)_12:0			0.8 ± 0.5	0.0 ± 0.0												
			16:0_12:0(*sn*-2)_14:1			0.8 ± 0.5	0.0 ± 0.0												
			16:0_14:1(*sn*-2)_12:0			1.5 ± 1.3	0.0 ± 0.0												
		14:0_14:0_14:1	14:0_14:0(*sn*-2)_14:1			0.8 ± 0.2	0.0 ± 0.0									1.5 ± 1.2	0.1 ± 0.1		
			14:0_14:1(*sn*-2)_14:0			0.8 ± 0.4	0.0 ± 0.0									0.6 ± 0.6	0.0 ± 0.0		
		6:0_18:0_18:1	18:0_18:1(*sn*-2)_6:0			8.6 ± 8.0	0.2 ± 0.2	0.3 ± 0.4	0.0 ± 0.0	13.3 ± 3.4	0.7 ± 0.2								
			18:0_6:0(*sn*-2)_18:1			4.1 ± 5.1	0.1 ± 0.1	3.5 ± 3.9	0.2 ± 0.2	1.4 ± 0.8	0.1 ± 0.0								
			18:1_18:0(*sn*-2)_6:0			9.8 ± 7.2	0.2 ± 0.2	9.5 ± 3.7	0.5 ± 0.2	11.0 ± 4.3	0.5 ± 0.2								
		8:0_16:0_18:1	16:0_18:1(*sn*-2)_8:0			10.6 ± 15.7	0.3 ± 0.4	0.8 ± 1.4	0.0 ± 0.1	13.4 ± 1.1	0.7 ± 0.1					3.6 ± 5.4	0.2 ± 0.3		
			18:1_16:0(*sn*-2)_8:0			3.5 ± 3.8	0.1 ± 0.1	6.6 ± 6.6	0.3 ± 0.3	4.4 ± 2.7	0.2 ± 0.1					22.5 ± 8.5	1.3 ± 0.5		
			18:1_8:0(*sn*-2)_16:0			23.1 ± 18.8	0.6 ± 0.5	27.8 ± 8.0	1.4 ± 0.4	20.4 ± 2.9	1.0 ± 0.2					4.6 ± 4.5	0.3 ± 0.3		
	**42:2**			**nd**		**0.7 ± 0.0**		**1.3 ± 0.2**		**1.5 ± 0.1**		**nd**		**nd**		**1.8 ± 0.4**		**nd**	
		10:0_14:0_18:2	14:0_18:2(*sn*-2)_10:0			5.8 ± 1.9	0.0 ± 0.0	4.6 ± 6.1	0.1 ± 0.1							1.1 ± 1.8	0.0 ± 0.0		
			18:2_10:0(*sn*-2)_14:0			0.5 ± 0.9	0.0 ± 0.0	6.9 ± 10.2	0.1 ± 0.1							0.5 ± 0.8	0.0 ± 0.0		
			18:2_14:0(*sn*-2)_10:0			1.1 ± 2.0	0.0 ± 0.0	8.4 ± 7.0	0.1 ± 0.1							18.2 ± 7.5	0.3 ± 0.2		
		10:0_14:1_18:1	14:1_18:1(*sn*-2)_10:0			2.3 ± 1.7	0.0 ± 0.0									4.8 ± 4.1	0.1 ± 0.1		
			18:1_10:0(*sn*-2)_14:1			0.2 ± 0.5	0.0 ± 0.0									0.6 ± 0.5	0.0 ± 0.0		
			18:1_14:1(*sn*-2)_10:0			3.0 ± 4.8	0.0 ± 0.0									2.7 ± 0.7	0.0 ± 0.0		
		10:0_16:1_16:1	16:1_10:0(*sn*-2)_16:1					2.5 ± 3.7	0.0 ± 0.1							7.4 ± 9.6	0.1 ± 0.2		
			16:1_16:1(*sn*-2)_10:0					2.8 ± 3.0	0.0 ± 0.0							5.4 ± 4.9	0.1 ± 0.1		
		12:0_12:0_18:2	12:0_18:2(*sn*-2)_12:0													0.4 ± 0.3	0.0 ± 0.0		
			18:2_12:0(*sn*-2)_12:0													7.6 ± 4.0	0.1 ± 0.1		
		14:0_14:1_14:1	14:0_14:1(*sn*-2)_14:1			3.3 ± 2.4	0.0 ± 0.0												
			14:1_14:0(*sn*-2)_14:1			0.2 ± 0.3	0.0 ± 0.0												
		6:0_18:0_18:2	18:0_18:2(*sn*-2)_6:0			3.9 ± 2.3	0.0 ± 0.0	0.0 ± 0.0	0.0 ± 0.0										
			18:0_6:0(*sn*-2)_18:2			0.3 ± 0.5	0.0 ± 0.0	0.0 ± 0.0	0.0 ± 0.0										
			18:2_18:0(*sn*-2)_6:0			7.2 ± 2.8	0.1 ± 0.0	10.5 ± 3.8	0.1 ± 0.1										
		6:0_18:1_18:1	18:1_18:1(*sn*-2)_6:0			63.5 ± 12.2	0.5 ± 0.1	48.9 ± 8.3	0.6 ± 0.2	91.6 ± 5.0	1.4 ± 0.2								
			18:1_6:0(*sn*-2)_18:1			0.5 ± 1.0	0.0 ± 0.0	2.7 ± 5.4	0.0 ± 0.1	0.3 ± 0.6	0.0 ± 0.0								
		8:0_16:0_18:2	16:0_18:2(*sn*-2)_8:0			7.1 ± 2.1	0.1 ± 0.0	4.3 ± 5.2	0.1 ± 0.1							5.2 ± 8.3	0.1 ± 0.2		
			18:2_16:0(*sn*-2)_8:0			1.7 ± 3.0	0.0 ± 0.0	5.2 ± 7.0	0.1 ± 0.1							8.7 ± 11.9	0.2 ± 0.3		
			18:2_8:0(*sn*-2)_16:0			0.6 ± 1.0	0.0 ± 0.0	5.9 ± 7.1	0.1 ± 0.1							9.5 ± 11.0	0.2 ± 0.2		
		8:0_16:1_18:1	16:1_18:1(*sn*-2)_8:0			3.8 ± 3.3	0.0 ± 0.0			4.2 ± 4.9	0.1 ± 0.1					11.8 ± 15.0	0.2 ± 0.3		
			18:1_16:1(*sn*-2)_8:0			2.7 ± 3.8	0.0 ± 0.0			3.7 ± 6.8	0.1 ± 0.1					7.6 ± 9.3	0.1 ± 0.2		
			18:1_8:0(*sn*-2)_16:1			0.2 ± 0.4	0.0 ± 0.0			0.2 ± 0.3	0.0 ± 0.0					12.6 ± 13.3	0.2 ± 0.3		
	**42:3**			**nd**		**nd**		**nd**		**nd**		**nd**		**nd**		**2.0 ± 0.2**		**nd**	
		10:0_14:0_18:3	14:0_18:3(*sn*-2)_10:0													0.9 ± 1.0	0.0 ± 0.0		
			18:3_10:0(*sn*-2)_14:0													8.3 ± 11.4	0.2 ± 0.2		
			18:3_14:0(*sn*-2)_10:0													21.4 ± 9.1	0.4 ± 0.2		
		12:0_12:0_18:3	12:0_18:3(*sn*-2)_12:0													2.5 ± 3.8	0.0 ± 0.1		
			18:3_12:0(*sn*-2)_12:0													9.9 ± 4.7	0.2 ± 0.1		
		8:0_16:0_18:3	16:0_18:3(*sn*-2)_8:0													5.5 ± 11.1	0.1 ± 0.2		
			18:3_16:0(*sn*-2)_8:0													29.1 ± 25.3	0.6 ± 0.6		
			18:3_8:0(*sn*-2)_16:0													11.9 ± 19.8	0.2 ± 0.4		
		8:0_16:1_18:2	16:1_18:2(*sn*-2)_8:0													2.9 ± 4.5	0.1 ± 0.1		
			18:2_16:1(*sn*-2)_8:0													4.1 ± 4.0	0.1 ± 0.1		
			18:2_8:0(*sn*-2)_16:1													3.6 ± 3.4	0.1 ± 0.1		
**44**	**44:0**			**2.2 ± 0.1**		**2.9 ± 0.3**		**3.3 ± 0.3**[e]		**1.9 ± 0.3**		**1.6 ± 0.2**		**nd**		**1.1 ± 0.1**		**nd**	
		10:0_16:0_18:0	16:0_18:0(*sn*-2)_10:0	0.2 ± 0.2	0.0 ± 0.0	1.2 ± 1.1	0.0 ± 0.0	5.6 ± 2.5	0.2 ± 0.1	11.3 ± 3.9	0.2 ± 0.1	15.0 ± 2.2	0.2 ± 0.1			4.4 ± 6.1	0.0 ± 0.1		
			18:0_10:0(*sn*-2)_16:0	3.9 ± 0.5	0.1 ± 0.0	6.5 ± 2.4	0.2 ± 0.1	5.5 ± 3.3	0.2 ± 0.1	3.8 ± 4.3	0.1 ± 0.1	0.3 ± 0.4	0.0 ± 0.0			5.4 ± 6.8	0.1 ± 0.1		
			18:0_16:0(*sn*-2)_10:0	0.7 ± 0.4	0.0 ± 0.0	16.3 ± 3.3	0.5 ± 0.1	28.1 ± 2.8	0.9 ± 0.2	33.4 ± 5.4	0.6 ± 0.2	6.4 ± 8.5	0.1 ± 0.1			3.9 ± 3.6	0.0 ± 0.0		
		12:0_14:0_18:0	14:0_18:0(*sn*-2)_12:0	0.2 ± 0.1	0.0 ± 0.0	0.5 ± 0.4	0.0 ± 0.0	0.8 ± 0.4	0.0 ± 0.0			19.0 ± 20.0	0.3 ± 0.4			2.4 ± 4.0	0.0 ± 0.0		
			18:0_12:0(*sn*-2)_14:0	8.4 ± 1.7	0.2 ± 0.1	3.2 ± 2.2	0.1 ± 0.1	2.2 ± 0.8	0.1 ± 0.0			14.7 ± 24.9	0.2 ± 0.4			2.5 ± 3.9	0.0 ± 0.0		
			18:0_14:0(*sn*-2)_12:0	3.2 ± 1.5	0.1 ± 0.0	3.6 ± 2.4	0.1 ± 0.1	2.3 ± 1.7	0.1 ± 0.1			7.7 ± 7.9	0.1 ± 0.1			0.7 ± 0.9	0.0 ± 0.0		
		12:0_16:0_16:0	16:0_12:0(*sn*-2)_16:0	10.5 ± 2.2	0.2 ± 0.1	6.3 ± 5.3	0.2 ± 0.2	3.7 ± 0.5	0.1 ± 0.0	6.4 ± 2.4	0.1 ± 0.1	12.0 ± 22.4	0.2 ± 0.4			0.4 ± 0.3	0.0 ± 0.0		
			16:0_16:0(*sn*-2)_12:0	7.0 ± 4.1	0.2 ± 0.1	31.1 ± 6.3	0.9 ± 0.3	27.9 ± 1.4	0.9 ± 0.1	19.7 ± 2.6	0.4 ± 0.1	19.7 ± 13.4	0.3 ± 0.3			60.7 ± 2.9	0.7 ± 0.1		
		14:0_14:0_16:0	14:0_16:0(*sn*-2)_14:0	35.4 ± 7.5	0.8 ± 0.2	21.3 ± 3.8	0.6 ± 0.2	15.8 ± 2.5	0.5 ± 0.1	11.8 ± 4.0	0.2 ± 0.1	8.8 ± 5.9	0.1 ± 0.1			17.6 ± 5.4	0.2 ± 0.1		
			16:0_14:0(*sn*-2)_14:0	30.5 ± 8.0	0.7 ± 0.2	7.8 ± 2.9	0.2 ± 0.1	6.0 ± 1.4	0.2 ± 0.1	4.4 ± 3.2	0.1 ± 0.1	2.0 ± 2.4	0.0 ± 0.0			3.4 ± 4.7	0.0 ± 0.1		
		18:0_18:0_8:0	18:0_18:0(*sn*-2)_8:0			3.1 ± 1.0	0.1 ± 0.0	4.0 ± 0.9	0.1 ± 0.0	8.6 ± 1.6	0.2 ± 0.1								
			18:0_8:0(*sn*-2)_18:0			0.3 ± 0.2	0.0 ± 0.0	0.4 ± 0.3	0.0 ± 0.0	0.6 ± 0.6	0.0 ± 0.0								

ACN	TG species	TG molecular species	TG regioisomer	Camel		Cow		Goat		Sheep		Human		Dog		Horse		Pig	
				in TG species	of total TGs	in TG species	of total TGs	in TG species	of total TGs	in TG species	of total TGs	in TG species	of total TGs	in TG species	of total TGs	in TG species	of total TGs	in TG species	of total TGs
	**44:1**			**0.8 ± 0.2**		**2.3 ± 0.1**		**5.9 ± 0.3^{bc}**		**5.9 ± 0.3^{bc}**		**5.7 ± 0.3**		**nd**		**5.8 ± 0.1^{b}**		**nd**	
		10:0_16:0_18:1	16:0_18:1(*sn*-2)_10:0	0.0 ± 0.1	0.0 ± 0.0	14.3 ± 12.6	0.3 ± 0.3	30.8 ± 10.1	1.8 ± 0.7	30.8 ± 10.1	1.8 ± 0.7	0.0 ± 0.0	0.0 ± 0.0			3.8 ± 6.2	0.2 ± 0.4		
			18:1_10:0(*sn*-2)_16:0	14.8 ± 5.5	0.1 ± 0.1	31.2 ± 5.2	0.7 ± 0.1	19.7 ± 9.7	1.2 ± 0.6	19.7 ± 9.7	1.2 ± 0.6	16.2 ± 12.7	0.9 ± 0.8			12.4 ± 9.3	0.7 ± 0.6		
			18:1_16:0(*sn*-2)_10:0	0.2 ± 0.2	0.0 ± 0.0	13.3 ± 11.1	0.3 ± 0.3	27.2 ± 3.6	1.6 ± 0.3	27.2 ± 3.6	1.6 ± 0.3	23.4 ± 13.4	1.3 ± 0.8			36.7 ± 6.4	2.1 ± 0.4		
		10:0_16:1_18:0	16:1_18:0(*sn*-2)_10:0													1.4 ± 0.7	0.1 ± 0.0		
			18:0_10:0(*sn*-2)_16:1													0.5 ± 0.3	0.0 ± 0.0		
			18:0_16:1(*sn*-2)_10:0													0.4 ± 0.4	0.0 ± 0.0		
		12:0_14:0_18:1	14:0_18:1(*sn*-2)_12:0	0.7 ± 1.2	0.0 ± 0.0	6.0 ± 2.4	0.1 ± 0.1	2.2 ± 1.6	0.1 ± 0.1	2.2 ± 1.6	0.1 ± 0.1	4.9 ± 9.7	0.3 ± 0.6			4.4 ± 2.7	0.3 ± 0.2		
			18:1_12:0(*sn*-2)_14:0	26.3 ± 7.9	0.2 ± 0.1	7.3 ± 4.6	0.2 ± 0.1	2.9 ± 1.0	0.2 ± 0.1	2.9 ± 1.0	0.2 ± 0.1	0.9 ± 0.7	0.1 ± 0.0			3.7 ± 2.4	0.2 ± 0.1		
			18:1_14:0(*sn*-2)_12:0	2.6 ± 4.8	0.0 ± 0.0	2.8 ± 2.3	0.1 ± 0.1	8.4 ± 4.7	0.5 ± 0.3	8.4 ± 4.7	0.5 ± 0.3	50.2 ± 9.1	2.9 ± 0.7			10.2 ± 3.0	0.6 ± 0.2		
		12:0_14:1_18:0	14:1_18:0(*sn*-2)_12:0	4.6 ± 6.5	0.0 ± 0.1	2.3 ± 2.1	0.1 ± 0.1												
			18:0_12:0(*sn*-2)_14:1	5.1 ± 6.6	0.0 ± 0.1	0.8 ± 0.7	0.0 ± 0.0												
			18:0_14:1(*sn*-2)_12:0	0.1 ± 0.1	0.0 ± 0.0	2.0 ± 2.7	0.0 ± 0.1												
		12:0_16:0_16:1	16:0_12:0(*sn*-2)_16:1	8.3 ± 7.3	0.1 ± 0.1											3.1 ± 1.5	0.2 ± 0.1		
			16:0_16:1(*sn*-2)_12:0	6.9 ± 13.4	0.1 ± 0.1											1.4 ± 2.1	0.1 ± 0.1		
			16:1_16:0(*sn*-2)_12:0	0.9 ± 1.7	0.0 ± 0.0											16.4 ± 5.6	1.0 ± 0.3		
		14:0_14:0_16:1	14:0_16:1(*sn*-2)_14:0	9.0 ± 17.2	0.1 ± 0.2							0.0 ± 0.0	0.0 ± 0.0			0.9 ± 1.0	0.1 ± 0.1		
			16:1_14:0(*sn*-2)_14:0	20.9 ± 14.2	0.2 ± 0.1							5.9 ± 1.7	0.3 ± 0.1			3.2 ± 0.6	0.2 ± 0.0		
		14:0_14:1_16:0	14:0_16:0(*sn*-2)_14:1	2.6 ± 3.8	0.0 ± 0.0	3.1 ± 2.1	0.1 ± 0.1	0.5 ± 0.3	0.0 ± 0.0	0.5 ± 0.3	0.0 ± 0.0					0.9 ± 0.4	0.1 ± 0.0		
			16:0_14:0(*sn*-2)_14:1	4.1 ± 0.5	0.0 ± 0.0	2.0 ± 1.4	0.0 ± 0.0	0.6 ± 0.2	0.0 ± 0.0	0.6 ± 0.2	0.0 ± 0.0					0.6 ± 0.3	0.0 ± 0.0		
			16:0_14:1(*sn*-2)_14:0	4.4 ± 7.5	0.0 ± 0.1	3.7 ± 4.7	0.1 ± 0.1	0.8 ± 0.3	0.0 ± 0.0	0.8 ± 0.3	0.0 ± 0.0					0.3 ± 0.1	0.0 ± 0.0		
		8:0_18:0_18:1	18:0_18:1(*sn*-2)_8:0			1.2 ± 1.7	0.0 ± 0.0	2.1 ± 0.6	0.1 ± 0.0	2.1 ± 0.6	0.1 ± 0.0								
			18:0_8:0(*sn*-2)_18:1			7.5 ± 2.9	0.2 ± 0.1	0.9 ± 1.1	0.1 ± 0.1	0.9 ± 1.1	0.1 ± 0.1								
			18:1_18:0(*sn*-2)_8:0			2.6 ± 2.8	0.1 ± 0.1	3.8 ± 1.8	0.2 ± 0.1	3.8 ± 1.8	0.2 ± 0.1								
	**44:2**			**nd**		**0.3 ± 0.0**		**0.6 ± 0.1**		**0.6 ± 0.1**		**0.9 ± 0.1**		**nd**		**2.8 ± 0.3**		**nd**	
		10:0_16:0_18:2	16:0_18:2(*sn*-2)_10:0			10.7 ± 5.4	0.0 ± 0.0	5.7 ± 4.9	0.0 ± 0.0	5.7 ± 4.9	0.0 ± 0.0	19.1 ± 16.6	0.2 ± 0.2			0.5 ± 0.8	0.0 ± 0.0		
			18:2_10:0(*sn*-2)_16:0			6.6 ± 6.3	0.0 ± 0.0	18.0 ± 6.8	0.1 ± 0.1	18.0 ± 6.8	0.1 ± 0.1	0.0 ± 0.0	0.0 ± 0.0			0.3 ± 0.5	0.0 ± 0.0		
			18:2_16:0(*sn*-2)_10:0			5.4 ± 3.6	0.0 ± 0.0	16.0 ± 16.1	0.1 ± 0.1	16.0 ± 16.1	0.1 ± 0.1	24.5 ± 24.1	0.2 ± 0.2			29.4 ± 2.9	0.8 ± 0.2		
		10:0_16:1_18:1	16:1_18:1(*sn*-2)_10:0			3.5 ± 2.9	0.0 ± 0.0	4.9 ± 2.3	0.0 ± 0.0	4.9 ± 2.3	0.0 ± 0.0					18.5 ± 9.1	0.5 ± 0.3		
			18:1_10:0(*sn*-2)_16:1			1.1 ± 0.9	0.0 ± 0.0	6.3 ± 4.8	0.0 ± 0.0	6.3 ± 4.8	0.0 ± 0.0					1.2 ± 1.7	0.0 ± 0.1		
			18:1_16:1(*sn*-2)_10:0			4.8 ± 4.3	0.0 ± 0.0	1.7 ± 1.9	0.0 ± 0.0	1.7 ± 1.9	0.0 ± 0.0					7.8 ± 8.2	0.2 ± 0.3		
		12:0_14:0_18:2	14:0_18:2(*sn*-2)_12:0			6.1 ± 3.3	0.0 ± 0.0	1.2 ± 1.3	0.0 ± 0.0	1.2 ± 1.3	0.0 ± 0.0	21.4 ± 16.2	0.2 ± 0.2			0.5 ± 0.6	0.0 ± 0.0		
			18:2_12:0(*sn*-2)_14:0			2.4 ± 2.9	0.0 ± 0.0	5.0 ± 4.2	0.0 ± 0.0	5.0 ± 4.2	0.0 ± 0.0	0.0 ± 0.1	0.0 ± 0.0			0.7 ± 1.2	0.0 ± 0.0		
			18:2_14:0(*sn*-2)_12:0			0.8 ± 0.6	0.0 ± 0.0	0.5 ± 0.4	0.0 ± 0.0	0.5 ± 0.4	0.0 ± 0.0	45.9 ± 38.3	0.4 ± 0.4			11.3 ± 4.2	0.3 ± 0.2		
		12:0_14:1_18:1	14:1_18:1(*sn*-2)_12:0			1.0 ± 0.2	0.0 ± 0.0									1.6 ± 0.9	0.0 ± 0.0		
			18:1_12:0(*sn*-2)_14:1			0.6 ± 0.6	0.0 ± 0.0									0.4 ± 0.4	0.0 ± 0.0		
			18:1_14:1(*sn*-2)_12:0			0.7 ± 0.6	0.0 ± 0.0									0.8 ± 0.8	0.0 ± 0.0		
		12:0_16:1_16:1	16:1_12:0(*sn*-2)_16:1			4.7 ± 4.9	0.0 ± 0.0	3.3 ± 3.3	0.0 ± 0.0	3.3 ± 3.3	0.0 ± 0.0					2.3 ± 2.2	0.1 ± 0.1		
			16:1_16:1(*sn*-2)_12:0			0.5 ± 0.4	0.0 ± 0.0	0.8 ± 0.1	0.0 ± 0.0	0.8 ± 0.1	0.0 ± 0.0					4.6 ± 2.5	0.1 ± 0.1		
		14:0_14:1_16:1	14:0_16:1(*sn*-2)_14:1			2.4 ± 1.0	0.0 ± 0.0												
			16:1_14:0(*sn*-2)_14:1			0.9 ± 0.5	0.0 ± 0.0												
			16:1_14:1(*sn*-2)_14:0			1.5 ± 1.4	0.0 ± 0.0												
		14:1_14:1_16:0	14:1_16:0(*sn*-2)_14:1			1.6 ± 0.9	0.0 ± 0.0												
			16:0_14:1(*sn*-2)_14:1			2.4 ± 2.7	0.0 ± 0.0												
		8:0_18:0_18:2	18:0_18:2(*sn*-2)_8:0			1.1 ± 1.2	0.0 ± 0.0												
			18:0_8:0(*sn*-2)_18:2			1.4 ± 1.5	0.0 ± 0.0												
			18:2_18:0(*sn*-2)_8:0			4.7 ± 5.3	0.0 ± 0.0												
		8:0_18:1_18:1	18:1_18:1(*sn*-2)_8:0			29.3 ± 19.6	0.1 ± 0.1	24.0 ± 16.6	0.1 ± 0.1	24.0 ± 16.6	0.1 ± 0.1					18.6 ± 6.3	0.5 ± 0.2		
			18:1_8:0(*sn*-2)_18:1			13.1 ± 19.0	0.0 ± 0.1	16.4 ± 17.4	0.1 ± 0.1	16.4 ± 17.4	0.1 ± 0.1					2.1 ± 3.5	0.1 ± 0.1		
	**44:3**			**nd**		**nd**		**nd**		**nd**		**nd**		**nd**		**3.2 ± 0.5**		**nd**	
		10:0_16:0_18:3	16:0_18:3(*sn*-2)_10:0													0.3 ± 0.4	0.0 ± 0.0		
			18:3_10:0(*sn*-2)_16:0													5.0 ± 4.3	0.2 ± 0.2		
			18:3_16:0(*sn*-2)_10:0													48.7 ± 11.4	1.5 ± 0.6		
		10:0_16:1_18:2	16:1_18:2(*sn*-2)_10:0													2.3 ± 3.6	0.1 ± 0.1		
			18:2_10:0(*sn*-2)_16:1													5.5 ± 4.2	0.2 ± 0.2		
			18:2_16:1(*sn*-2)_10:0													4.7 ± 7.7	0.1 ± 0.3		
		12:0_14:0_18:3	14:0_18:3(*sn*-2)_12:0													0.3 ± 0.3	0.0 ± 0.0		
			18:3_12:0(*sn*-2)_14:0													5.5 ± 5.0	0.2 ± 0.2		
			18:3_14:0(*sn*-2)_12:0													15.7 ± 6.4	0.5 ± 0.3		
		8:0_18:1_18:2	18:1_18:2(*sn*-2)_8:0													2.2 ± 3.1	0.1 ± 0.1		
			18:1_8:0(*sn*-2)_18:2													1.3 ± 2.0	0.0 ± 0.1		
			18:2_18:1(*sn*-2)_8:0													8.2 ± 3.5	0.3 ± 0.2		

ACN	TG species	TG molecular species	TG regioisomer	Camel		Cow		Goat		Sheep		Human		Dog		Horse		Pig	
				in TG species	of total TGs	in TG species	of total TGs	in TG species	of total TGs	in TG species	of total TGs	in TG species	of total TGs	in TG species	of total TGs	in TG species	of total TGs	in TG species	of total TGs
	**44:4**			**nd**		**nd**		**nd**		**nd**		**nd**		**nd**		**1.4 ± 0.1**		**nd**	
		10:0_16:1_18:3	16:1_18:3(*sn*-2)_10:0													0.0 ± 0.0	0.0 ± 0.0		
			18:3_10:0(*sn*-2)_16:1													7.7 ± 6.7	0.1 ± 0.1		
			18:3_16:1(*sn*-2)_10:0													37.3 ± 10.1	0.5 ± 0.2		
		8:0_18:1_18:3	18:1_18:3(*sn*-2)_8:0													1.7 ± 2.9	0.0 ± 0.0		
			18:1_8:0(*sn*-2)_18:3													31.1 ± 9.2	0.4 ± 0.1		
			18:3_18:1(*sn*-2)_8:0													22.3 ± 10.3	0.3 ± 0.2		
**46**	**46:0**			**5.3 ± 0.3**		**2.4 ± 0.1**		**1.4 ± 0.1**		**0.9 ± 0.2**		**1.9 ± 0.1**		**nd**		**0.7 ± 0.1**		**nd**	
		10:0_18:0_18:0	18:0_10:0(*sn*-2)_18:0			1.2 ± 0.4	0.0 ± 0.0	0.6 ± 0.4	0.0 ± 0.0	0.4 ± 0.3	0.0 ± 0.0								
			18:0_18:0(*sn*-2)_10:0			3.0 ± 0.6	0.1 ± 0.0	13.0 ± 0.5	0.2 ± 0.0	26.1 ± 3.9	0.2 ± 0.1								
		12:0_16:0_18:0	16:0_18:0(*sn*-2)_12:0	0.1 ± 0.1	0.0 ± 0.0	0.3 ± 0.3	0.0 ± 0.0	1.0 ± 0.6	0.0 ± 0.0	3.9 ± 3.5	0.0 ± 0.0	16.0 ± 3.5	0.3 ± 0.1			2.1 ± 3.6	0.0 ± 0.0		
			18:0_12:0(*sn*-2)_16:0	13.3 ± 2.9	0.7 ± 0.2	21.9 ± 2.5	0.5 ± 0.1	18.1 ± 11.1	0.3 ± 0.2	5.8 ± 7.2	0.1 ± 0.1	17.8 ± 4.1	0.3 ± 0.1			9.5 ± 10.0	0.1 ± 0.1		
			18:0_16:0(*sn*-2)_12:0	0.2 ± 0.1	0.0 ± 0.0	0.8 ± 0.8	0.0 ± 0.0	7.1 ± 6.9	0.1 ± 0.1	20.7 ± 5.5	0.2 ± 0.1	25.5 ± 8.4	0.5 ± 0.2			5.9 ± 6.7	0.0 ± 0.0		
		14:0_14:0_18:0	14:0_18:0(*sn*-2)_14:0	10.8 ± 3.2	0.6 ± 0.2	11.8 ± 2.1	0.3 ± 0.1			5.4 ± 3.1	0.0 ± 0.0	11.5 ± 3.6	0.2 ± 0.1			3.9 ± 3.0	0.0 ± 0.0		
			18:0_14:0(*sn*-2)_14:0	17.5 ± 5.9	0.9 ± 0.4	1.9 ± 0.7	0.0 ± 0.0			0.7 ± 0.4	0.0 ± 0.0	6.2 ± 5.2	0.1 ± 0.1			0.7 ± 0.8	0.0 ± 0.0		
		14:0_16:0_16:0	16:0_14:0(*sn*-2)_16:0	33.4 ± 4.4	1.8 ± 0.3	20.2 ± 10.1	0.5 ± 0.3	13.7 ± 10.1	0.2 ± 0.2	4.7 ± 6.6	0.0 ± 0.1	10.2 ± 3.9	0.2 ± 0.1			9.9 ± 11.3	0.1 ± 0.1		
			16:0_16:0(*sn*-2)_14:0	21.0 ± 4.7	1.1 ± 0.3	38.9 ± 13.5	0.9 ± 0.4	46.5 ± 8.5	0.7 ± 0.2	33.8 ± 11.1	0.3 ± 0.2	12.9 ± 1.6	0.2 ± 0.0			68.1 ± 13.5	0.5 ± 0.1		
		15:0_15:0_16:0	15:0_16:0(*sn*-2)_15:0	1.6 ± 0.6	0.1 ± 0.0														
			16:0_15:0(*sn*-2)_15:0	2.0 ± 0.7	0.1 ± 0.0														
	**46:1**			**3.6 ± 0.1**		**2.7 ± 0.3**		**3.0 ± 0.2[e]**		**2.9 ± 0.3**		**10.2 ± 0.5[b]**		**0.9 ± 0.1**		**4.8 ± 0.6[c]**		**nd**	
		10:0_18:0_18:1	18:0_10:0(*sn*-2)_18:1			6.0 ± 3.5	0.2 ± 0.1	20.4 ± 4.5	0.6 ± 0.2	33.1 ± 3.3	1.0 ± 0.2								
			18:0_18:1(*sn*-2)_10:0			0.5 ± 0.3	0.0 ± 0.0	2.5 ± 1.7	0.1 ± 0.1	1.4 ± 1.4	0.0 ± 0.0								
			18:1_18:0(*sn*-2)_10:0			6.4 ± 3.4	0.2 ± 0.1	11.3 ± 5.9	0.3 ± 0.2	13.3 ± 4.6	0.4 ± 0.2								
		12:0_16:0_18:1	16:0_18:1(*sn*-2)_12:0	3.1 ± 2.9	0.1 ± 0.1	1.3 ± 1.3	0.0 ± 0.0	18.8 ± 4.0	0.6 ± 0.2	15.0 ± 5.6	0.4 ± 0.2	6.3 ± 12.5	0.6 ± 1.3	0.0 ± 0.0	0.0 ± 0.0	0.0 ± 0.0	0.0 ± 0.0		
			18:1_12:0(*sn*-2)_16:0	6.1 ± 0.4	0.2 ± 0.0	32.5 ± 7.1	0.9 ± 0.3	27.9 ± 5.8	0.8 ± 0.2	18.9 ± 2.8	0.6 ± 0.1	20.2 ± 2.3	2.1 ± 0.3	0.0 ± 0.0	0.0 ± 0.0	19.6 ± 7.5	1.0 ± 0.5		
			18:1_16:0(*sn*-2)_12:0	4.9 ± 4.1	0.2 ± 0.2	7.8 ± 6.5	0.2 ± 0.2	0.9 ± 0.8	0.0 ± 0.0	2.6 ± 3.5	0.1 ± 0.1	49.6 ± 11.9	5.1 ± 1.5	29.0 ± 3.5	0.3 ± 0.1	41.1 ± 8.0	2.0 ± 0.6		
		12:0_16:1_18:0	16:1_18:0(*sn*-2)_12:0	2.0 ± 0.6	0.1 ± 0.0	3.5 ± 0.9	0.1 ± 0.0					0.0 ± 0.0	0.0 ± 0.0						
			18:0_12:0(*sn*-2)_16:1	1.7 ± 1.3	0.1 ± 0.0	1.0 ± 0.4	0.0 ± 0.0					2.9 ± 1.3	0.3 ± 0.2						
			18:0_16:1(*sn*-2)_12:0	1.2 ± 1.1	0.0 ± 0.0	0.3 ± 0.2	0.0 ± 0.0					0.0 ± 0.0	0.0 ± 0.0						
		14:0_14:0_18:1	14:0_18:1(*sn*-2)_14:0	3.6 ± 3.1	0.1 ± 0.1	0.3 ± 0.2	0.0 ± 0.0	4.9 ± 4.9	0.1 ± 0.2	1.2 ± 0.9	0.0 ± 0.0	1.1 ± 2.2	0.1 ± 0.2			0.0 ± 0.0	0.0 ± 0.0		
			18:1_14:0(*sn*-2)_14:0	35.0 ± 7.5	1.3 ± 0.3	19.4 ± 1.2	0.5 ± 0.1	13.3 ± 4.2	0.4 ± 0.1	11.1 ± 2.3	0.3 ± 0.1	20.7 ± 2.4	2.1 ± 0.3			14.5 ± 4.2	0.7 ± 0.3		
		14:0_14:1_18:0	14:0_18:0(*sn*-2)_14:1	1.3 ± 0.6	0.0 ± 0.0	4.0 ± 0.8	0.1 ± 0.0												
			18:0_14:0(*sn*-2)_14:1	1.0 ± 0.4	0.0 ± 0.0	0.9 ± 0.5	0.0 ± 0.0												
			18:3_16:0(*sn*-2)_10:0													48.7 ± 11.4	1.5 ± 0.6		
		14:0_16:0_16:1	16:0_14:0(*sn*-2)_16:1	13.5 ± 2.4	0.5 ± 0.1	2.8 ± 0.4	0.1 ± 0.0			0.8 ± 0.4	0.0 ± 0.0			0.0 ± 0.0	0.0 ± 0.0	24.8 ± 5.7	1.2 ± 0.4		
			16:0_16:1(*sn*-2)_14:0	4.2 ± 5.1	0.2 ± 0.2	0.4 ± 0.2	0.0 ± 0.0			2.1 ± 2.0	0.1 ± 0.1			0.0 ± 0.0	0.0 ± 0.0	0.0 ± 0.0	0.0 ± 0.0		
			16:1_16:0(*sn*-2)_14:0	15.0 ± 5.3	0.5 ± 0.2	2.7 ± 0.3	0.1 ± 0.0			0.5 ± 0.2	0.0 ± 0.0			62.2 ± 3.1	0.6 ± 0.1	0.0 ± 0.0	0.0 ± 0.0		
		14:1_16:0_16:0	16:0_14:1(*sn*-2)_16:0	2.8 ± 3.2	0.1 ± 0.1	1.6 ± 1.5	0.0 ± 0.0							0.0 ± 0.0	0.0 ± 0.0				
			16:0_16:0(*sn*-2)_14:1	2.0 ± 1.2	0.1 ± 0.0	8.5 ± 2.3	0.2 ± 0.1							8.8 ± 2.4	0.1 ± 0.0				
		15:0_15:0_16:1	15:0_16:1(*sn*-2)_15:0	0.8 ± 0.6	0.0 ± 0.0														
			16:1_15:0(*sn*-2)_15:0	1.2 ± 0.4	0.0 ± 0.0														
	**46:2**			**0.9 ± 0.1**		**0.4 ± 0.3**		**1.0 ± 0.2**		**1.2 ± 0.1**		**1.5 ± 0.0**		**nd**		**2.2 ± 0.0**		**nd**	
		10:0_18:0_18:2	18:0_10:0(*sn*-2)_18:2			4.3 ± 2.1	0.0 ± 0.0	1.5 ± 1.3	0.0 ± 0.0	0.6 ± 1.0	0.0 ± 0.0								
			18:0_18:2(*sn*-2)_10:0			0.5 ± 0.7	0.0 ± 0.0	6.7 ± 1.9	0.1 ± 0.0	9.1 ± 2.5	0.1 ± 0.0								
			18:2_18:0(*sn*-2)_10:0			4.5 ± 1.1	0.0 ± 0.0	1.1 ± 1.0	0.0 ± 0.0	0.5 ± 0.9	0.0 ± 0.0								
		10:0_18:1_18:1	18:1_10:0(*sn*-2)_18:1			42.0 ± 6.2	0.2 ± 0.1	48.6 ± 6.5	0.5 ± 0.2	31.9 ± 15.6	0.4 ± 0.2					8.0 ± 5.6	0.2 ± 0.1		
			18:1_18:1(*sn*-2)_10:0			6.5 ± 10.1	0.0 ± 0.1	26.7 ± 6.7	0.3 ± 0.1	46.2 ± 17.2	0.6 ± 0.3					16.9 ± 5.5	0.4 ± 0.1		
		12:0_16:0_18:2	16:0_18:2(*sn*-2)_12:0	0.6 ± 0.7	0.0 ± 0.0	5.5 ± 6.9	0.0 ± 0.0	6.9 ± 2.0	0.1 ± 0.0	5.8 ± 2.4	0.1 ± 0.0	9.9 ± 9.4	0.1 ± 0.1			14.1 ± 5.2	0.3 ± 0.1		
			18:2_12:0(*sn*-2)_16:0	22.0 ± 1.7	0.2 ± 0.0	6.9 ± 5.4	0.0 ± 0.0	4.2 ± 3.5	0.0 ± 0.0	3.5 ± 3.6	0.0 ± 0.0	12.0 ± 13.8	0.2 ± 0.2			6.4 ± 4.1	0.1 ± 0.1		
			18:2_16:0(*sn*-2)_12:0	0.1 ± 0.1	0.0 ± 0.0	3.7 ± 5.0	0.0 ± 0.0	1.7 ± 1.6	0.0 ± 0.0	0.2 ± 0.4	0.0 ± 0.0	59.0 ± 7.1	0.9 ± 0.1			7.8 ± 4.8	0.2 ± 0.1		
		12:0_16:1_18:1	16:1_18:1(*sn*-2)_12:0	0.3 ± 0.3	0.0 ± 0.0	0.8 ± 1.1	0.0 ± 0.0									11.8 ± 1.9	0.3 ± 0.0		
			18:1_12:0(*sn*-2)_16:1	11.0 ± 5.4	0.1 ± 0.1	3.5 ± 3.2	0.0 ± 0.0									5.8 ± 3.4	0.1 ± 0.1		
			18:1_16:1(*sn*-2)_12:0	0.2 ± 0.2	0.0 ± 0.0	1.4 ± 1.7	0.0 ± 0.0									14.9 ± 6.8	0.3 ± 0.2		
		14:0_14:0_18:2	14:0_18:2(*sn*-2)_14:0	8.9 ± 6.2	0.1 ± 0.1	3.9 ± 4.3	0.0 ± 0.0	1.8 ± 0.2	0.0 ± 0.0	2.0 ± 0.7	0.0 ± 0.0	12.7 ± 8.5	0.2 ± 0.1			3.4 ± 1.6	0.1 ± 0.0		
			18:2_14:0(*sn*-2)_14:0	13.2 ± 7.0	0.1 ± 0.1	3.5 ± 2.4	0.0 ± 0.0	0.8 ± 0.7	0.0 ± 0.0	0.2 ± 0.4	0.0 ± 0.0	6.4 ± 8.4	0.1 ± 0.1			2.2 ± 0.8	0.0 ± 0.0		
		14:0_14:1_18:1	14:0_18:1(*sn*-2)_14:1	7.0 ± 5.8	0.1 ± 0.1	1.1 ± 1.1	0.0 ± 0.0												
			18:1_14:0(*sn*-2)_14:1	7.2 ± 13.2	0.1 ± 0.1	5.4 ± 6.2	0.0 ± 0.0												
			18:1_14:1(*sn*-2)_14:0	7.8 ± 6.7	0.1 ± 0.1	1.5 ± 1.3	0.0 ± 0.0												
		14:0_16:1_16:1	16:1_14:0(*sn*-2)_16:1	12.0 ± 6.9	0.1 ± 0.1	1.3 ± 0.4	0.0 ± 0.0									4.5 ± 3.1	0.1 ± 0.1		
			16:1_16:1(*sn*-2)_14:0	8.3 ± 5.7	0.1 ± 0.1	1.4 ± 0.2	0.0 ± 0.0									4.2 ± 1.3	0.1 ± 0.0		

ACN	TG species	TG molecular species	TG regioisomer	Camel		Cow		Goat		Sheep		Human		Dog		Horse		Pig	
				in TG species	of total TGs	in TG species	of total TGs	in TG species	of total TGs	in TG species	of total TGs	in TG species	of total TGs	in TG species	of total TGs	in TG species	of total TGs	in TG species	of total TGs
		14:1_14:1_18:0	14:1_18:0(*sn*-2)_14:1			0.6 ± 0.9	0.0 ± 0.0												
			18:0_14:1(*sn*-2)_14:1			4.1 ± 3.0	0.0 ± 0.0												
		14:1_16:0_16:1	16:0_14:1(*sn*-2)_16:1	0.8 ± 0.4	0.0 ± 0.0	3.3 ± 4.9	0.0 ± 0.0												
			16:0_16:1(*sn*-2)_14:1	0.5 ± 0.2	0.0 ± 0.0	0.7 ± 1.3	0.0 ± 0.0												
			16:1_16:0(*sn*-2)_14:1	0.4 ± 0.1	0.0 ± 0.0	2.5 ± 3.2	0.0 ± 0.0												
	**46:3**			**nd**		**nd**		**nd**		**nd**		**nd**		**nd**		**2.3 ± 0.5**		**nd**	
		10:0_18:1_18:2	18:1_10:0(*sn*-2)_18:2													9.5 ± 6.7	0.2 ± 0.2		
			18:1_18:2(*sn*-2)_10:0													12.5 ± 7.2	0.3 ± 0.2		
			18:2_18:1(*sn*-2)_10:0													0.0 ± 0.0	0.0 ± 0.0		
		12:0_16:0_18:3	16:0_18:3(*sn*-2)_12:0													2.6 ± 4.5	0.1 ± 0.1		
			18:3_12:0(*sn*-2)_16:0													17.9 ± 3.5	0.4 ± 0.2		
			18:3_16:0(*sn*-2)_12:0													36.7 ± 1.6	0.8 ± 0.2		
		12:0_16:1_18:2	16:1_18:2(*sn*-2)_12:0													6.5 ± 5.8	0.1 ± 0.2		
			18:2_12:0(*sn*-2)_16:1													3.2 ± 5.5	0.1 ± 0.1		
			18:2_16:1(*sn*-2)_12:0													0.0 ± 0.0	0.0 ± 0.0		
		14:0_14:0_18:3	14:0_18:3(*sn*-2)_14:0													0.4 ± 0.7	0.0 ± 0.0		
			18:3_14:0(*sn*-2)_14:0													10.6 ± 1.7	0.2 ± 0.1		
	**46:4**			**nd**		**nd**		**nd**		**nd**		**nd**		**nd**		**1.4 ± 0.1**		**nd**	
		10:0_18:1_18:3	18:1_10:0(*sn*-2)_18:3													40.9 ± 19.9	0.6 ± 0.3		
			18:1_18:3(*sn*-2)_10:0													3.2 ± 6.5	0.0 ± 0.1		
			18:3_18:1(*sn*-2)_10:0													27.2 ± 18.5	0.4 ± 0.3		
		10:0_18:2_18:2	18:2_10:0(*sn*-2)_18:2													8.4 ± 1.5	0.1 ± 0.0		
			18:2_18:2(*sn*-2)_10:0													0.0 ± 0.0	0.0 ± 0.0		
		12:0_16:1_18:3	16:1_18:3(*sn*-2)_12:0													0.0 ± 0.1	0.0 ± 0.0		
			18:3_12:0(*sn*-2)_16:1													12.2 ± 6.9	0.2 ± 0.1		
			18:3_16:1(*sn*-2)_12:0													17.7 ± 6.3	0.2 ± 0.1		
47	**47:0**			**0.4 ± 0.0**		**nd**		**nd**		**nd**		**nd**		**nd**		**nd**		**nd**	
		14:0_15:0_18:0	15:0_18:0(*sn*-2)_14:0	2.0 ± 3.6	0.0 ± 0.0														
			18:0_14:0(*sn*-2)_15:0	28.1 ± 14.9	0.1 ± 0.1														
			18:0_15:0(*sn*-2)_14:0	6.4 ± 8.0	0.0 ± 0.0														
		14:0_16:0_17:0	16:0_17:0(*sn*-2)_14:0	3.0 ± 5.8	0.0 ± 0.0														
			17:0_14:0(*sn*-2)_16:0	20.3 ± 11.1	0.1 ± 0.0														
			17:0_16:0(*sn*-2)_14:0	1.2 ± 2.1	0.0 ± 0.0														
		15:0_15:0_17:0	15:0_17:0(*sn*-2)_15:0	0.5 ± 0.7	0.0 ± 0.0														
			17:0_15:0(*sn*-2)_15:0	4.4 ± 0.3	0.0 ± 0.0														
		15:0_16:0_16:0	16:0_15:0(*sn*-2)_16:0	33.9 ± 2.7	0.1 ± 0.0														
			16:0_16:0(*sn*-2)_15:0	1.4 ± 2.7	0.0 ± 0.0														
	**47:1**			**1.6 ± 0.4**		**nd**		**nd**		**nd**		**nd**		**nd**		**nd**		**nd**	
		14:0_15:0_18:1	15:0_18:1(*sn*-2)_14:0	0.0 ± 0.0	0.0 ± 0.0														
			18:1_14:0(*sn*-2)_15:0	6.3 ± 5.7	0.1 ± 0.1														
			18:1_15:0(*sn*-2)_14:0	55.6 ± 3.8	0.9 ± 0.3														
		15:0_16:0_16:1	16:0_15:0(*sn*-2)_16:1	19.6 ± 18.2	0.3 ± 0.4														
			16:0_16:1(*sn*-2)_15:0	18.5 ± 20.0	0.3 ± 0.4														
			16:1_16:0(*sn*-2)_15:0	0.0 ± 0.0	0.0 ± 0.0														
48	**48:0**			**6.5 ± 0.2[e]**		**1.8 ± 0.1**		**0.7 ± 0.1**		**0.5 ± 0.1**	**6.5 ± 0.2[e]**	**0.7 ± 0.1**		**1.2 ± 0.2**		**0.3 ± 0**		**0.6 ± 0**	
		12:0_18:0_18:0	18:0_12:0(*sn*-2)_18:0	3.7 ± 0.6	0.2 ± 0.0	7.0 ± 1.9	0.1 ± 0.0			13.9 ± 4.6	0.1 ± 0.0								
			18:0_18:0(*sn*-2)_12:0	0.1 ± 0.2	0.0 ± 0.0	0.6 ± 0.9	0.0 ± 0.0			4.4 ± 3.3	0.0 ± 0.0								
		14:0_16:0_18:0	16:0_18:0(*sn*-2)_14:0	19.6 ± 5.5	1.3 ± 0.4	12.7 ± 5.0	0.2 ± 0.1	10.5 ± 7.5	0.1 ± 0.1	21.2 ± 2.3	0.1 ± 0.0	33.2 ± 24.1	0.2 ± 0.2	27.8 ± 7.0	0.3 ± 0.1				
			18:0_14:0(*sn*-2)_16:0	46.0 ± 9.5	3.0 ± 0.7	36.1 ± 10.9	0.7 ± 0.2	43.5 ± 24.0	0.3 ± 0.2	19.5 ± 0.7	0.1 ± 0.0	27.3 ± 31.9	0.2 ± 0.3	0.5 ± 0.2	0.0 ± 0.0				
			18:0_16:0(*sn*-2)_14:0	3.1 ± 4.1	0.2 ± 0.3	6.3 ± 6.9	0.1 ± 0.1	9.2 ± 10.6	0.1 ± 0.1	20.9 ± 1.2	0.1 ± 0.0	11.3 ± 19.4	0.1 ± 0.2	8.0 ± 6.5	0.1 ± 0.1				
		14:0_17:0_17:0	17:0_14:0(*sn*-2)_17:0	0.9 ± 0.1	0.1 ± 0.0														
			17:0_17:0(*sn*-2)_14:0	0.7 ± 0.1	0.0 ± 0.0														
		15:0_15:0_18:0	15:0_18:0(*sn*-2)_15:0	0.3 ± 0.2	0.0 ± 0.0														
			18:0_15:0(*sn*-2)_15:0	1.4 ± 0.7	0.1 ± 0.0														
		16:0_16:0_16:0	16:0_16:0(*sn*-2)_16:0	25.2 ± 1.5	1.6 ± 0.1	37.3 ± 3.2	0.7 ± 0.1	36.8 ± 6.7	0.3 ± 0.1	20.0 ± 1.7	0.1 ± 0.0	28.2 ± 6.1	0.2 ± 0.1	63.7 ± 2.5	0.8 ± 0.2	100.0 ± 0.0	0.3 ± 0.0	100.0 ± 0.0	0.6 ± 0.0
	**48:1**			**9.8 ± 0.4[c]**		**3.6 ± 0.1**		**2.9 ± 0.0[e]**		**2.1 ± 0.1**		**8.0 ± 0.4[d]**		**5.2 ± 0.1[e]**		**3.9 ± 0.1[d]**		**1.4 ± 0.1**	
		12:0_18:0_18:1	18:0_12:0(*sn*-2)_18:1	2.5 ± 0.7	0.2 ± 0.1	11.9 ± 2.1	0.4 ± 0.1	10.1 ± 6.8	0.3 ± 0.2	21.0 ± 5.9	0.4 ± 0.1	17.6 ± 6.8	1.4 ± 0.6						
			18:0_18:1(*sn*-2)_12:0	0.3 ± 0.2	0.0 ± 0.0	0.1 ± 0.1	0.0 ± 0.0	3.4 ± 6.5	0.1 ± 0.2	2.9 ± 5.0	0.1 ± 0.1	0.1 ± 0.1	0.0 ± 0.0						
			18:1_18:0(*sn*-2)_12:0	2.1 ± 1.1	0.2 ± 0.1	0.6 ± 1.0	0.0 ± 0.0	1.6 ± 1.9	0.0 ± 0.1	2.1 ± 3.4	0.0 ± 0.1	6.0 ± 5.3	0.5 ± 0.4						

ACN	TG species	TG molecular species	TG regioisomer	Camel		Cow		Goat		Sheep		Human		Dog		Horse		Pig	
				in TG species	of total TGs	in TG species	of total TGs	in TG species	of total TGs	in TG species	of total TGs	in TG species	of total TGs	in TG species	of total TGs	in TG species	of total TGs	in TG species	of total TGs
		14:0_16:0_18:1	16:0_18:1(*sn*-2)_14:0	1.5 ± 1.4	0.1 ± 0.1	18.0 ± 12.6	0.7 ± 0.5	6.3 ± 8.1	0.2 ± 0.2	9.0 ± 8.0	0.2 ± 0.2	6.1 ± 5.4	0.5 ± 0.4	0.0 ± 0.0	0.0 ± 0.0	0.0 ± 0.0	0.0 ± 0.0	1.2 ± 2.3	0.0 ± 0.0
			18:1_14:0(*sn*-2)_16:0	57.8 ± 4.0	5.7 ± 0.6	66.4 ± 13.1	2.4 ± 0.6	73.0 ± 10.6	2.1 ± 0.3	59.7 ± 2.9	1.3 ± 0.1	45.7 ± 8.5	3.6 ± 0.8	0.0 ± 0.0	0.0 ± 0.0	22.6 ± 6.9	0.9 ± 0.3	40.1 ± 7.2	0.6 ± 0.2
			18:1_16:0(*sn*-2)_14:0	5.3 ± 3.2	0.5 ± 0.3	3.0 ± 3.2	0.1 ± 0.1	5.7 ± 3.9	0.2 ± 0.1	5.2 ± 5.1	0.1 ± 0.1	24.5 ± 9.0	2.0 ± 0.8	54.8 ± 2.7	2.9 ± 0.2	46.1 ± 6.7	1.8 ± 0.3	22.2 ± 8.7	0.3 ± 0.2
		14:0_16:1_18:0	16:1_18:0(*sn*-2)_14:0	15.6 ± 1.4	1.5 ± 0.2														
			18:0_14:0(*sn*-2)_16:1	2.4 ± 3.3	0.2 ± 0.3														
			18:0_16:1(*sn*-2)_14:0	0.1 ± 0.1	0.0 ± 0.0														
		14:1_16:0_18:0	16:0_18:0(*sn*-2)_14:1	1.6 ± 0.4	0.2 ± 0.0														
			18:0_14:1(*sn*-2)_16:0	0.2 ± 0.1	0.0 ± 0.0														
			18:0_16:0(*sn*-2)_14:1	0.5 ± 0.2	0.1 ± 0.0														
		15:0_15:0_18:1	15:0_18:1(*sn*-2)_15:0	0.2 ± 0.2	0.0 ± 0.0														
			18:1_15:0(*sn*-2)_15:0	1.2 ± 0.4	0.1 ± 0.0														
		16:0_16:0_16:1	16:0_16:0(*sn*-2)_16:1	7.8 ± 1.8	0.8 ± 0.2									45.2 ± 2.7	2.3 ± 0.2	31.3 ± 2.4	1.2 ± 0.1	36.1 ± 5.9	0.5 ± 0.1
			16:0_16:1(*sn*-2)_16:0	1.7 ± 1.7	0.2 ± 0.2									0.0 ± 0.0	0.0 ± 0.0	0.0 ± 0.0	0.0 ± 0.0	0.4 ± 0.9	0.0 ± 0.0
	**48:2**			**2.4 ± 0.3**		**0.6 ± 0.2**		**0.7 ± 0.1**		**0.8 ± 0.1**		**2.7 ± 0.3**		**2.2 ± 0.1**		**2.2 ± 0.3**		**0.5 ± 0.0**	
		12:0_18:0_18:2	18:0_12:0(*sn*-2)_18:2	1.5 ± 0.8	0.0 ± 0.0	3.5 ± 4.7	0.0 ± 0.0	2.7 ± 2.6	0.0 ± 0.0	8.2 ± 0.9	0.1 ± 0.0	12.6 ± 1.5	0.3 ± 0.1			2.0 ± 1.1	0.0 ± 0.0		
			18:0_18:2(*sn*-2)_12:0	0.7 ± 0.3	0.0 ± 0.0	0.3 ± 0.3	0.0 ± 0.0	2.2 ± 3.1	0.0 ± 0.0	0.1 ± 0.1	0.0 ± 0.0	0.1 ± 0.1	0.0 ± 0.0			0.4 ± 0.3	0.0 ± 0.0		
			18:2_18:0(*sn*-2)_12:0	0.5 ± 0.2	0.0 ± 0.0	1.1 ± 1.0	0.0 ± 0.0	0.3 ± 0.2	0.0 ± 0.0	0.3 ± 0.4	0.0 ± 0.0	0.3 ± 0.4	0.0 ± 0.0			0.5 ± 0.8	0.0 ± 0.0		
		12:0_18:1_18:1	18:1_12:0(*sn*-2)_18:1	12.4 ± 4.0	0.3 ± 0.1	25.6 ± 1.0	0.1 ± 0.1	31.1 ± 21.4	0.2 ± 0.2	39.8 ± 28.6	0.3 ± 0.3	42.6 ± 3.2	1.1 ± 0.2			23.1 ± 5.4	0.5 ± 0.2		
			18:1_18:1(*sn*-2)_12:0	1.3 ± 1.3	0.0 ± 0.0	3.5 ± 3.3	0.0 ± 0.0	14.7 ± 17.4	0.1 ± 0.1	23.6 ± 32.1	0.2 ± 0.3	0.1 ± 0.1	0.0 ± 0.0			3.2 ± 5.9	0.1 ± 0.1		
		14:0_16:0_18:2	16:0_18:2(*sn*-2)_14:0	15.2 ± 2.9	0.4 ± 0.1	1.2 ± 2.0	0.0 ± 0.0	11.1 ± 12.6	0.1 ± 0.1	0.1 ± 0.2	0.0 ± 0.0	3.6 ± 7.2	0.1 ± 0.2	0.0 ± 0.0	0.0 ± 0.0	1.9 ± 1.4	0.0 ± 0.0	0.1 ± 0.2	0.0 ± 0.0
			18:2_14:0(*sn*-2)_16:0	12.7 ± 2.6	0.3 ± 0.1	13.4 ± 5.9	0.1 ± 0.1	16.7 ± 14.4	0.1 ± 0.1	14.0 ± 7.6	0.1 ± 0.1	20.7 ± 5.5	0.6 ± 0.2	0.0 ± 0.0	0.0 ± 0.0	14.7 ± 4.8	0.3 ± 0.1	52.7 ± 4.5	0.2 ± 0.0
			18:2_16:0(*sn*-2)_14:0	6.5 ± 1.8	0.2 ± 0.1	13.1 ± 7.6	0.1 ± 0.1	1.4 ± 1.9	0.0 ± 0.0	10.0 ± 8.6	0.1 ± 0.1	4.5 ± 4.8	0.1 ± 0.1	41.4 ± 3.5	0.9 ± 0.1	3.1 ± 4.2	0.1 ± 0.1	5.1 ± 4.6	0.0 ± 0.0
		14:0_16:1_18:1	16:1_18:1(*sn*-2)_14:0	9.6 ± 1.8	0.2 ± 0.1	1.0 ± 1.0	0.0 ± 0.0	0.4 ± 0.5	0.0 ± 0.0	0.1 ± 0.1	0.0 ± 0.0	1.4 ± 2.7	0.0 ± 0.1	0.0 ± 0.0	0.0 ± 0.0	2.8 ± 3.4	0.1 ± 0.1	0.2 ± 0.5	0.0 ± 0.0
			18:1_14:0(*sn*-2)_16:1	8.2 ± 1.1	0.2 ± 0.0	8.1 ± 7.7	0.0 ± 0.1	3.1 ± 5.1	0.0 ± 0.0	4.7 ± 4.1	0.0 ± 0.0	7.3 ± 4.4	0.2 ± 0.1	7.7 ± 4.8	0.2 ± 0.1	16.5 ± 7.0	0.4 ± 0.2	31.6 ± 6.5	0.1 ± 0.0
			18:1_16:1(*sn*-2)_14:0	9.4 ± 1.9	0.2 ± 0.1	0.8 ± 0.8	0.0 ± 0.0	6.6 ± 11.6	0.0 ± 0.1	7.4 ± 11.3	0.1 ± 0.1	4.7 ± 5.2	0.1 ± 0.2	0.0 ± 0.0	0.0 ± 0.0	9.9 ± 7.9	0.2 ± 0.2	0.1 ± 0.1	0.0 ± 0.0
		14:1_16:0_18:1	16:0_18:1(*sn*-2)_14:1	2.0 ± 0.6	0.0 ± 0.0	1.6 ± 2.8	0.0 ± 0.0	0.2 ± 0.3	0.0 ± 0.0					0.0 ± 0.0	0.0 ± 0.0				
			18:1_14:1(*sn*-2)_16:0	2.7 ± 1.7	0.1 ± 0.0	1.6 ± 2.7	0.0 ± 0.0	13.9 ± 16.3	0.1 ± 0.1					0.0 ± 0.0	0.0 ± 0.0				
			18:1_16:0(*sn*-2)_14:1	2.0 ± 0.5	0.0 ± 0.0	23.4 ± 3.3	0.1 ± 0.1	3.2 ± 5.2	0.0 ± 0.0					17.8 ± 2.7	0.4 ± 0.1				
		14:1_16:1_18:0	16:1_18:0(*sn*-2)_14:1	1.7 ± 0.9	0.0 ± 0.0	1.0 ± 0.1	0.0 ± 0.0	0.9 ± 1.0	0.0 ± 0.0										
			18:0_14:1(*sn*-2)_16:1	1.2 ± 0.4	0.0 ± 0.0	0.5 ± 0.1	0.0 ± 0.0	0.4 ± 0.0	0.0 ± 0.0										
			18:0_16:1(*sn*-2)_14:1	1.0 ± 0.3	0.0 ± 0.0	0.6 ± 0.1	0.0 ± 0.0	0.3 ± 0.0	0.0 ± 0.0										
		16:0_16:1_16:1	16:0_16:1(*sn*-2)_16:1	9.5 ± 3.6	0.2 ± 0.1	1.0 ± 1.1	0.0 ± 0.0	4.5 ± 3.2	0.0 ± 0.0	4.5 ± 6.1	0.0 ± 0.1	2.2 ± 2.0	0.1 ± 0.1	0.0 ± 0.0	0.0 ± 0.0	16.4 ± 7.0	0.4 ± 0.2	0.7 ± 1.4	0.0 ± 0.0
			16:1_16:0(*sn*-2)_16:1	2.0 ± 0.8	0.0 ± 0.0	5.1 ± 4.7	0.0 ± 0.0	1.3 ± 1.4	0.0 ± 0.0	2.2 ± 3.6	0.0 ± 0.0	0.8 ± 0.9	0.0 ± 0.0	33.0 ± 4.8	0.7 ± 0.1	5.6 ± 7.3	0.1 ± 0.2	9.6 ± 6.0	0.0 ± 0.0
	**48:3**			**1.2 ± 0.1**		**nd**		**nd**		**nd**		**0.7 ± 0.1**		**0.2 ± 0.1**		**1.7 ± 0.3**		**nd**	
		12:0_18:1_18:2	18:1_12:0(*sn*-2)_18:2									32.3 ± 55.9	0.2 ± 0.5			20.6 ± 5.8	0.3 ± 0.2		
			18:1_18:2(*sn*-2)_12:0									18.4 ± 29.5	0.1 ± 0.2			0.9 ± 1.8	0.0 ± 0.0		
			18:2_18:1(*sn*-2)_12:0									49.3 ± 49.8	0.4 ± 0.4			2.4 ± 2.8	0.0 ± 0.1		
		14:0_16:0_18:3	16:0_18:3(*sn*-2)_14:0	0.0 ± 0.0	0.0 ± 0.0											12.3 ± 19.9	0.2 ± 0.4		
			18:3_14:0(*sn*-2)_16:0	42.0 ± 7.9	0.5 ± 0.1											28.1 ± 19.9	0.5 ± 0.4		
			18:3_16:0(*sn*-2)_14:0	10.2 ± 17.7	0.1 ± 0.2											17.8 ± 17.9	0.3 ± 0.4		
		14:0_16:1_18:2	16:1_18:2(*sn*-2)_14:0	0.0 ± 0.0	0.0 ± 0.0									0.0 ± 0.0	0.0 ± 0.0	1.4 ± 1.8	0.0 ± 0.0		
			18:2_14:0(*sn*-2)_16:1	45.9 ± 8.5	0.6 ± 0.1									60.8 ± 3.9	0.1 ± 0.1	10.5 ± 8.1	0.2 ± 0.2		
			18:2_16:1(*sn*-2)_14:0	1.9 ± 2.4	0.0 ± 0.0									0.1 ± 0.3	0.0 ± 0.0	4.0 ± 4.3	0.1 ± 0.1		
		14:1_16:1_18:1	16:1_18:1(*sn*-2)_14:1											39.1 ± 3.8	0.1 ± 0.0				
			18:1_14:1(*sn*-2)_16:1											0.0 ± 0.0	0.0 ± 0.0				
			18:1_16:1(*sn*-2)_14:1											0.0 ± 0.0	0.0 ± 0.0				
		16:1_16:1_16:1	16:1_16:1(*sn*-2)_16:1													6.0 ± 3.5	0.1 ± 0.1		
	**48:4**			**nd**		**nd**		**nd**		**nd**		**nd**		**nd**		**1.6 ± 0.2**		**nd**	
		12:0_18:1_18:3	18:1_12:0(*sn*-2)_18:3													24.0 ± 28.8	0.4 ± 0.5		
			18:1_18:3(*sn*-2)_12:0													14.0 ± 11.4	0.2 ± 0.2		
			18:3_18:1(*sn*-2)_12:0													26.4 ± 29.2	0.4 ± 0.5		
		14:0_16:1_18:3	16:1_18:3(*sn*-2)_14:0													6.5 ± 12.1	0.1 ± 0.2		
			18:3_14:0(*sn*-2)_16:1													9.4 ± 7.5	0.2 ± 0.1		
			18:3_16:1(*sn*-2)_14:0													19.8 ± 13.9	0.3 ± 0.3		
**49**	**49:0**			**0.7 ± 0.0**		**nd**		**nd**		**nd**		**nd**		**nd**		**nd**		**nd**	
		14:0_17:0_18:0	17:0_18:0(*sn*-2)_14:0	1.1 ± 1.2	0.0 ± 0.0														
			18:0_14:0(*sn*-2)_17:0	11.9 ± 8.2	0.1 ± 0.1														
			18:0_17:0(*sn*-2)_14:0	8.1 ± 6.2	0.1 ± 0.0														
		15:0_16:0_18:0	16:0_18:0(*sn*-2)_15:0	15.9 ± 12.7	0.1 ± 0.1														
			18:0_15:0(*sn*-2)_16:0	48.2 ± 15.3	0.3 ± 0.1														
			18:0_16:0(*sn*-2)_15:0	1.0 ± 1.1	0.0 ± 0.0														

ACN	TG species	TG molecular species	TG regioisomer	Camel		Cow		Goat		Sheep		Human		Dog		Horse		Pig	
				in TG species	of total TGs	in TG species	of total TGs	in TG species	of total TGs	in TG species	of total TGs	in TG species	of total TGs	in TG species	of total TGs	in TG species	of total TGs	in TG species	of total TGs
		15:0_17:0_17:0	17:0_15:0(*sn*-2)_17:0	1.7 ± 1.1	0.0 ± 0.0														
			17:0_17:0(*sn*-2)_15:0	0.2 ± 0.2	0.0 ± 0.0														
		16:0_16:0_17:0	16:0_17:0(*sn*-2)_16:0	0.3 ± 0.3	0.0 ± 0.0														
			17:0_16:0(*sn*-2)_16:0	12.6 ± 3.0	0.1 ± 0.0														
	**49:1**			**0.9 ± 0.0**		**nd**		**nd**		**nd**		**nd**		**nd**		**nd**		**nd**	
		14:0_17:0_18:1	17:0_18:1(*sn*-2)_14:0	0.6 ± 0.8	0.0 ± 0.0														
			18:1_14:0(*sn*-2)_17:0	11.8 ± 7.7	0.1 ± 0.1														
			18:1_17:0(*sn*-2)_14:0	5.1 ± 6.0	0.0 ± 0.1														
		14:0_17:1_18:0	17:1_18:0(*sn*-2)_14:0	1.0 ± 1.4	0.0 ± 0.0														
			18:0_14:0(*sn*-2)_17:1	4.4 ± 3.7	0.0 ± 0.0														
			18:0_17:1(*sn*-2)_14:0	0.2 ± 0.2	0.0 ± 0.0														
		15:0_16:0_18:1	16:0_18:1(*sn*-2)_15:0	6.6 ± 7.9	0.1 ± 0.1														
			18:1_15:0(*sn*-2)_16:0	31.4 ± 10.9	0.3 ± 0.1														
			18:1_16:0(*sn*-2)_15:0	16.4 ± 12.9	0.2 ± 0.1														
		15:0_16:1_18:0	16:1_18:0(*sn*-2)_15:0	3.3 ± 5.0	0.0 ± 0.0														
			18:0_15:0(*sn*-2)_16:1	4.7 ± 4.3	0.0 ± 0.0														
			18:0_16:1(*sn*-2)_15:0	5.4 ± 8.6	0.1 ± 0.1														
		15:0_17:0_17:1	17:0_15:0(*sn*-2)_17:1	1.0 ± 0.4	0.0 ± 0.0														
			17:0_17:1(*sn*-2)_15:0	0.4 ± 0.4	0.0 ± 0.0														
			17:1_17:0(*sn*-2)_15:0	0.8 ± 0.7	0.0 ± 0.0														
		15:1_16:0_18:0	16:0_18:0(*sn*-2)_15:1	1.5 ± 2.1	0.0 ± 0.0														
			18:0_15:1(*sn*-2)_16:0	4.0 ± 5.6	0.0 ± 0.1														
			18:0_16:0(*sn*-2)_15:1	2.7 ± 3.7	0.0 ± 0.0														
		16:0_16:0_17:1	16:0_17:1(*sn*-2)_16:0	0.9 ± 1.3	0.0 ± 0.0														
			17:1_16:0(*sn*-2)_16:0	3.1 ± 0.5	0.0 ± 0.0														
		16:0_16:1_17:0	16:0_17:0(*sn*-2)_16:1	3.1 ± 2.3	0.0 ± 0.0														
			17:0_16:0(*sn*-2)_16:1	2.0 ± 0.8	0.0 ± 0.0														
			17:0_16:1(*sn*-2)_16:0	0.3 ± 0.5	0.0 ± 0.0														
	**49:2**			**0.4 ± 0.1**		**nd**		**nd**		**nd**		**nd**		**nd**		**nd**		**nd**	
		14:0_17:0_18:2	17:0_18:2(*sn*-2)_14:0	0.3 ± 0.5	0.0 ± 0.0														
			18:2_14:0(*sn*-2)_17:0	0.1 ± 0.2	0.0 ± 0.0														
			18:2_17:0(*sn*-2)_14:0	13.0 ± 7.1	0.0 ± 0.0														
		15:0_16:0_18:2	16:0_18:2(*sn*-2)_15:0	1.9 ± 3.7	0.0 ± 0.0														
			18:2_15:0(*sn*-2)_16:0	12.6 ± 20.9	0.0 ± 0.1														
			18:2_16:0(*sn*-2)_15:0	12.9 ± 11.2	0.0 ± 0.1														
		15:0_16:1_18:1	16:1_18:1(*sn*-2)_15:0	8.7 ± 10.1	0.0 ± 0.0														
			18:1_15:0(*sn*-2)_16:1	26.0 ± 24.9	0.1 ± 0.1														
			18:1_16:1(*sn*-2)_15:0	0.7 ± 1.5	0.0 ± 0.0														
		15:0_17:1_17:1	17:1_15:0(*sn*-2)_17:1	5.3 ± 7.1	0.0 ± 0.0														
			17:1_17:1(*sn*-2)_15:0	1.5 ± 2.2	0.0 ± 0.0														
		15:1_16:0_18:1	16:0_18:1(*sn*-2)_15:1	17.2 ± 16.9	0.1 ± 0.1														
			18:1_15:1(*sn*-2)_16:0	0.2 ± 0.4	0.0 ± 0.0														
			18:1_16:0(*sn*-2)_15:1	12.0 ± 13.7	0.0 ± 0.1														
		16:0_16:1_17:1	16:0_17:1(*sn*-2)_16:1	1.4 ± 1.9	0.0 ± 0.0														
			17:1_16:0(*sn*-2)_16:1	2.0 ± 0.9	0.0 ± 0.0														
			17:1_16:1(*sn*-2)_16:0	0.8 ± 1.1	0.0 ± 0.0														
		16:1_16:1_17:0	16:1_17:0(*sn*-2)_16:1	5.9 ± 0.5	0.0 ± 0.0														
			17:0_16:1(*sn*-2)_16:1	0.1 ± 0.2	0.0 ± 0.0														
		15:0_17:0_17:0	17:0_15:0(*sn*-2)_17:0	1.7 ± 1.1	0.0 ± 0.0														
			17:0_17:0(*sn*-2)_15:0	0.2 ± 0.2	0.0 ± 0.0														
		16:0_16:0_17:0	16:0_17:0(*sn*-2)_16:0	0.3 ± 0.3	0.0 ± 0.0														
			17:0_16:0(*sn*-2)_16:0	12.6 ± 3.0	0.1 ± 0.0														
**50**	**50:0**			**5.1 ± 0.3**		**1.1 ± 0.1**		**0.5 ± 0.1**		**0.4 ± 0.1**		**0.9 ± 0.1**		**0.7 ± 0.4**		**nd**		**0.7 ± 0.0**	
		14:0_16:0_20:0	16:0_20:0(*sn*-2)_14:0	0.1 ± 0.1	0.0 ± 0.0	0.0 ± 0.0	0.0 ± 0.0												
			20:0_14:0(*sn*-2)_16:0	1.0 ± 0.4	0.1 ± 0.0	1.3 ± 0.3	0.0 ± 0.0												
			20:0_16:0(*sn*-2)_14:0	0.8 ± 0.2	0.0 ± 0.0	1.2 ± 0.3	0.0 ± 0.0												
		14:0_18:0_18:0	18:0_14:0(*sn*-2)_18:0	20.8 ± 2.3	1.1 ± 0.2	17.2 ± 3.2	0.2 ± 0.1	20.5 ± 4.8	0.1 ± 0.0	23.9 ± 3.7	0.1 ± 0.0								
			18:0_18:0(*sn*-2)_14:0	2.8 ± 1.1	0.1 ± 0.1	3.5 ± 1.6	0.0 ± 0.0	5.5 ± 7.0	0.0 ± 0.0	11.1 ± 1.5	0.0 ± 0.0								
		16:0_16:0_18:0	16:0_18:0(*sn*-2)_16:0	11.7 ± 4.3	0.6 ± 0.3	19.9 ± 2.1	0.2 ± 0.0	21.6 ± 18.1	0.1 ± 0.1	36.9 ± 10.3	0.1 ± 0.1	13.7 ± 20.6	0.1 ± 0.2	39.8 ± 4.8	0.3 ± 0.2			65.4 ± 11.7	0.4 ± 0.1
			18:0_16:0(*sn*-2)_16:0	62.3 ± 5.6	3.2 ± 0.4	57.4 ± 3.7	0.6 ± 0.1	52.5 ± 20.1	0.3 ± 0.1	28.1 ± 14.3	0.1 ± 0.1	86.3 ± 20.6	0.8 ± 0.2	60.3 ± 4.8	0.4 ± 0.3			34.6 ± 11.7	0.2 ± 0.1

ACN	TG species	TG molecular species	TG regioisomer	Camel		Cow		Goat		Sheep		Human		Dog		Horse		Pig	
				in TG species	of total TGs	in TG species	of total TGs	in TG species	of total TGs	in TG species	of total TGs	in TG species	of total TGs	in TG species	of total TGs	in TG species	of total TGs	in TG species	of total TGs
		16:0_17:0_17:0	17:0_16:0(*sn*-2)_17:0	0.6 ± 0.1	0.0 ± 0.0														
			17:0_17:0(*sn*-2)_16:0	0.7 ± 0.1	0.0 ± 0.0														
	**50:1**			**12.8 ± 0.3**^{a}		**4.3 ± 0.2**^{de}		**2.9 ± 0.2**^{e}		**1.8 ± 0.1**		**8.6 ± 0.2**^{c}		**11.1 ± 0.3**^{d}		**3.4 ± 0.1**		**8.3 ± 0.1**^{c}	
		14:0_18:0_18:1	18:0_14:0(*sn*-2)_18:1	33.9 ± 5.9	4.3 ± 0.9	28.1 ± 1.6	1.2 ± 0.1	24.7 ± 4.0	0.7 ± 0.2	33.0 ± 5.2	0.6 ± 0.1	30.7 ± 8.5	2.6 ± 0.8	0.0 ± 0.0	0.0 ± 0.0			7.6 ± 1.2	0.6 ± 0.1
			18:0_18:1(*sn*-2)_14:0	0.4 ± 0.7	0.0 ± 0.1	0.0 ± 0.0	0.0 ± 0.0	0.1 ± 0.3	0.0 ± 0.0	3.0 ± 2.4	0.1 ± 0.0	0.0 ± 0.0	0.0 ± 0.0	0.0 ± 0.0	0.0 ± 0.0			0.0 ± 0.1	0.0 ± 0.0
			18:1_18:0(*sn*-2)_14:0	3.3 ± 4.1	0.4 ± 0.5	0.6 ± 1.2	0.0 ± 0.1	3.3 ± 3.2	0.1 ± 0.1	4.0 ± 3.3	0.1 ± 0.1	3.0 ± 5.8	0.3 ± 0.5	11.4 ± 0.8	1.3 ± 0.1			1.7 ± 2.9	0.1 ± 0.2
		16:0_16:0_18:1	16:0_18:1(*sn*-2)_16:0	8.4 ± 14.1	1.1 ± 1.8	5.9 ± 6.5	0.3 ± 0.3	3.3 ± 6.7	0.1 ± 0.2	8.8 ± 6.9	0.2 ± 0.1	1.8 ± 3.6	0.2 ± 0.3	0.0 ± 0.0	0.0 ± 0.0	1.4 ± 2.2	0.0 ± 0.1	4.0 ± 5.5	0.3 ± 0.5
			18:1_16:0(*sn*-2)_16:0	40.7 ± 9.9	5.2 ± 1.4	65.4 ± 6.8	2.8 ± 0.4	68.6 ± 7.9	2.0 ± 0.3	51.3 ± 7.1	0.9 ± 0.2	64.5 ± 5.5	5.6 ± 0.6	75.1 ± 1.8	8.3 ± 0.4	98.6 ± 2.2	3.4 ± 0.2	89.1 ± 7.8	7.4 ± 0.7
		16:0_16:1_18:0	16:0_18:0(*sn*-2)_16:1	4.8 ± 4.8	0.6 ± 0.6									6.8 ± 8.2	0.8 ± 0.9				
			18:0_16:0(*sn*-2)_16:1	8.8 ± 6.5	1.1 ± 0.9									6.8 ± 5.7	0.8 ± 0.7				
			18:0_16:1(*sn*-2)_16:0	3.1 ± 5.6	0.4 ± 0.7									0.0 ± 0.0	0.0 ± 0.0				
	**50:2**			**8.5 ± 0.5**^{d}		**1.4 ± 0.2**		**1.1 ± 0.1**		**0.7 ± 0.1**		**7.1 ± 0.3**^{e}		**11.9 ± 0.7**^{c}		**5.0 ± 0.2**^{c}		**8.0 ± 0.1**^{c}	
		14:0_18:0_18:2	18:0_14:0(*sn*-2)_18:2	5.0 ± 0.8	0.4 ± 0.1			4.9 ± 2.5	0.1 ± 0.0	7.9 ± 1.8	0.1 ± 0.0			0.0 ± 0.0	0.0 ± 0.0			1.7 ± 1.2	0.1 ± 0.1
			18:0_18:2(*sn*-2)_14:0	1.5 ± 1.0	0.1 ± 0.1			0.2 ± 0.3	0.0 ± 0.0	1.1 ± 1.6	0.0 ± 0.0			0.0 ± 0.0	0.0 ± 0.0			0.2 ± 0.3	0.0 ± 0.0
			18:2_18:0(*sn*-2)_14:0	3.4 ± 2.2	0.3 ± 0.2			1.8 ± 2.7	0.0 ± 0.0	1.0 ± 0.2	0.0 ± 0.0			18.7 ± 2.4	2.2 ± 0.4			2.0 ± 1.9	0.2 ± 0.2
		14:0_18:1_18:1	18:1_14:0(*sn*-2)_18:1	33.6 ± 3.6	2.8 ± 0.5	60.8 ± 1.1	0.8 ± 0.1	61.3 ± 7.2	0.7 ± 0.1	38.1 ± 34.8	0.3 ± 0.3	32.4 ± 31.1	2.3 ± 2.3			28.4 ± 6.8	1.4 ± 0.4	11.1 ± 5.8	0.9 ± 0.5
			18:1_18:1(*sn*-2)_14:0	4.3 ± 3.7	0.4 ± 0.3	0.0 ± 0.0	0.0 ± 0.0	0.3 ± 0.5	0.0 ± 0.0	29.7 ± 34.1	0.2 ± 0.3	21.5 ± 24.9	1.5 ± 1.9			0.2 ± 0.2	0.0 ± 0.0	3.3 ± 5.8	0.3 ± 0.5
		16:0_16:0_18:2	16:0_18:2(*sn*-2)_16:0	3.9 ± 2.8	0.3 ± 0.3	0.0 ± 0.0	0.0 ± 0.0	5.3 ± 6.2	0.1 ± 0.1	5.2 ± 7.4	0.0 ± 0.1			0.0 ± 0.0	0.0 ± 0.0			0.4 ± 0.8	0.0 ± 0.1
			18:2_16:0(*sn*-2)_16:0	14.4 ± 4.9	1.2 ± 0.5	20.9 ± 2.2	0.3 ± 0.1	12.8 ± 4.7	0.1 ± 0.1	10.1 ± 4.1	0.1 ± 0.0			15.4 ± 1.0	1.8 ± 0.2			47.5 ± 1.5	3.8 ± 0.2
		16:0_16:1_18:1	16:0_18:1(*sn*-2)_16:1	4.6 ± 2.5	0.4 ± 0.2	0.0 ± 0.0	0.0 ± 0.0	0.8 ± 1.1	0.0 ± 0.0	0.0 ± 0.0	0.0 ± 0.0	0.0 ± 0.0	0.0 ± 0.0	0.0 ± 0.0	0.0 ± 0.0	2.4 ± 4.1	0.1 ± 0.2	4.2 ± 7.1	0.3 ± 0.6
			18:1_16:0(*sn*-2)_16:1	14.8 ± 6.1	1.3 ± 0.6	7.0 ± 9.9	0.1 ± 0.1	7.1 ± 7.3	0.1 ± 0.1	4.3 ± 7.4	0.0 ± 0.1	20.4 ± 23.7	1.5 ± 1.8	54.4 ± 2.1	6.5 ± 0.6	47.5 ± 27.4	2.4 ± 1.5	28.9 ± 7.5	2.3 ± 0.6
			18:1_16:1(*sn*-2)_16:0	7.5 ± 3.4	0.6 ± 0.3	5.8 ± 6.6	0.1 ± 0.1	10.1 ± 12.5	0.1 ± 0.1	21.9 ± 19.0	0.2 ± 0.1	25.7 ± 29.7	1.8 ± 2.2	0.0 ± 0.0	0.0 ± 0.0	21.4 ± 27.2	1.1 ± 1.4	0.7 ± 1.3	0.1 ± 0.1
		16:1_16:1_18:0	16:1_18:0(*sn*-2)_16:1	2.7 ± 1.6	0.2 ± 0.1	0.0 ± 0.0	0.0 ± 0.0							11.5 ± 1.4	1.4 ± 0.2				
			18:0_16:1(*sn*-2)_16:1	4.5 ± 2.0	0.4 ± 0.2	7.3 ± 0.6	0.1 ± 0.0							0.0 ± 0.0	0.0 ± 0.0				
	**50:3**			**0.6 ± 0.1**		**nd**		**nd**		**nd**		**1.1 ± 0.3**		**3.8 ± 0.1**		**3.2 ± 0.2**		**2.7 ± 0.0**	
		14:0_18:0_18:3	18:0_14:0(*sn*-2)_18:3	5.5 ± 6.0	0.0 ± 0.0														
			18:0_18:3(*sn*-2)_14:0	2.1 ± 1.8	0.0 ± 0.0														
			18:3_18:0(*sn*-2)_14:0	4.7 ± 6.3	0.0 ± 0.0														
		14:0_18:1_18:2	18:1_14:0(*sn*-2)_18:2	30.2 ± 6.1	0.2 ± 0.1							74.2 ± 4.7	0.8 ± 0.2	0.0 ± 0.0	0.0 ± 0.0	19.1 ± 5.6	0.6 ± 0.2	27.2 ± 3.9	0.7 ± 0.1
			18:1_18:2(*sn*-2)_14:0	4.3 ± 5.0	0.0 ± 0.0							0.0 ± 0.0	0.0 ± 0.0	0.0 ± 0.0	0.0 ± 0.0	1.4 ± 1.7	0.0 ± 0.1	3.5 ± 3.5	0.1 ± 0.1
			18:2_18:1(*sn*-2)_14:0	2.8 ± 3.3	0.0 ± 0.0							0.6 ± 1.0	0.0 ± 0.0	24.9 ± 2.9	1.0 ± 0.1	2.1 ± 2.5	0.1 ± 0.1	3.4 ± 2.1	0.1 ± 0.1
		14:1_18:0_18:2	18:0_14:1(*sn*-2)_18:2	1.4 ± 0.0	0.0 ± 0.0														
			18:0_18:2(*sn*-2)_14:1	1.2 ± 0.1	0.0 ± 0.0														
			18:2_18:0(*sn*-2)_14:1	1.3 ± 0.2	0.0 ± 0.0														
		14:1_18:1_18:1	18:1_14:1(*sn*-2)_18:1	2.5 ± 1.8	0.0 ± 0.0														
			18:1_18:1(*sn*-2)_14:1	3.5 ± 2.5	0.0 ± 0.0														
		16:0_16:0_18:3	16:0_18:3(*sn*-2)_16:0	3.7 ± 4.9	0.0 ± 0.0							0.0 ± 0.0	0.0 ± 0.0			6.0 ± 7.5	0.2 ± 0.3	3.8 ± 2.3	0.1 ± 0.1
			18:3_16:0(*sn*-2)_16:0	7.7 ± 6.8	0.0 ± 0.0							16.3 ± 3.8	0.2 ± 0.1			40.1 ± 7.8	1.3 ± 0.4	7.2 ± 2.0	0.2 ± 0.1
		16:0_16:1_18:2	16:0_18:2(*sn*-2)_16:1	1.4 ± 1.6	0.0 ± 0.0									0.0 ± 0.0	0.0 ± 0.0	2.1 ± 2.4	0.1 ± 0.1	5.6 ± 2.6	0.1 ± 0.1
			18:2_16:0(*sn*-2)_16:1	12.4 ± 17.5	0.1 ± 0.1									60.1 ± 2.9	2.3 ± 0.2	9.3 ± 3.8	0.3 ± 0.1	17.5 ± 6.4	0.5 ± 0.2
			18:2_16:1(*sn*-2)_16:0	7.0 ± 6.4	0.0 ± 0.0									0.0 ± 0.0	0.0 ± 0.0	3.4 ± 3.9	0.1 ± 0.1	24.5 ± 7.4	0.7 ± 0.2
		16:1_16:1_18:1	16:1_18:1(*sn*-2)_16:1	4.4 ± 5.0	0.0 ± 0.0							4.3 ± 5.1	0.0 ± 0.1	15.0 ± 4.3	0.6 ± 0.2	9.9 ± 7.0	0.3 ± 0.3	2.8 ± 2.2	0.1 ± 0.1
			18:1_16:1(*sn*-2)_16:1	11.4 ± 7.6	0.1 ± 0.1							4.6 ± 7.3	0.0 ± 0.1	0.0 ± 0.0	0.0 ± 0.0	6.6 ± 4.7	0.2 ± 0.2	8.7 ± 2.7	0.2 ± 0.1
	**50:4**			**0.6 ± 0.1**		**nd**		**nd**		**nd**		**nd**		**0.2 ± 0.1**		**3.6 ± 0.3**^{de}		**0.2 ± 0.1**	
		14:0_18:1_18:3	18:1_14:0(*sn*-2)_18:3	33.4 ± 16.5	0.2 ± 0.1									0.0 ± 0.0	0.0 ± 0.0	47.4 ± 6.7	1.7 ± 0.4		
			18:1_18:3(*sn*-2)_14:0	12.4 ± 14.5	0.1 ± 0.1									0.0 ± 0.0	0.0 ± 0.0	0.0 ± 0.0	0.0 ± 0.0		
			18:3_18:1(*sn*-2)_14:0	0.0 ± 0.0	0.0 ± 0.0									30.2 ± 15.4	0.1 ± 0.0	0.0 ± 0.0	0.0 ± 0.0		
		14:0_18:2_18:2	18:2_14:0(*sn*-2)_18:2	13.7 ± 8.6	0.1 ± 0.1									16.5 ± 3.1	0.0 ± 0.0			30.7 ± 14.2	0.1 ± 0.1
			18:2_18:2(*sn*-2)_14:0	0.0 ± 0.0	0.0 ± 0.0									0.0 ± 0.0	0.0 ± 0.0			36.5 ± 9.4	0.1 ± 0.0
		14:1_18:1_18:2	18:1_14:1(*sn*-2)_18:2											0.0 ± 0.0	0.0 ± 0.0				
			18:1_18:2(*sn*-2)_14:1											0.0 ± 0.0	0.0 ± 0.0				
			18:2_18:1(*sn*-2)_14:1											18.1 ± 2.7	0.0 ± 0.0				
		16:0_16:1_18:3	16:0_18:3(*sn*-2)_16:1	13.0 ± 16.2	0.1 ± 0.1									0.0 ± 0.0	0.0 ± 0.0	0.0 ± 0.0	0.0 ± 0.0		
			18:3_16:0(*sn*-2)_16:1	2.6 ± 5.2	0.0 ± 0.0									48.0 ± 1.0	0.1 ± 0.0	22.3 ± 16.5	0.8 ± 0.7		
			18:3_16:1(*sn*-2)_16:0	8.3 ± 9.6	0.0 ± 0.1									0.0 ± 0.0	0.0 ± 0.0	30.3 ± 18.9	1.1 ± 0.8		
		16:1_16:1_18:2	16:1_18:2(*sn*-2)_16:1	0.0 ± 0.0	0.0 ± 0.0									0.0 ± 0.0	0.0 ± 0.0			17.0 ± 7.6	0.0 ± 0.0
			18:2_16:1(*sn*-2)_16:1	16.7 ± 11.0	0.1 ± 0.1									22.3 ± 0.9	0.0 ± 0.0			15.8 ± 4.7	0.0 ± 0.0

ACN	TG species	TG molecular species	TG regioisomer	Camel		Cow		Goat		Sheep		Human		Dog		Horse		Pig	
				in TG species	of total TGs	in TG species	of total TGs	in TG species	of total TGs	in TG species	of total TGs	in TG species	of total TGs	in TG species	of total TGs	in TG species	of total TGs	in TG species	of total TGs
**51**	**51:1**			**1.4 ± 0.1**		**nd**		**nd**		**nd**		**nd**		**nd**		**nd**		**nd**	
		15:0_18:0_18:1	18:0_15:0(*sn*-2)_18:1	51.7 ± 5.4	0.7 ± 0.1														
			18:0_18:1(*sn*-2)_15:0	0.1 ± 0.1	0.0 ± 0.0														
			18:1_18:0(*sn*-2)_15:0	1.4 ± 2.7	0.0 ± 0.0														
		16:0_17:0_18:1	17:0_18:1(*sn*-2)_16:0	10.0 ± 9.8	0.1 ± 0.1														
			18:1_16:0(*sn*-2)_17:0	11.6 ± 1.1	0.2 ± 0.0														
			18:1_17:0(*sn*-2)_16:0	12.8 ± 9.5	0.2 ± 0.1														
		16:0_17:1_18:0	17:1_18:0(*sn*-2)_16:0	1.0 ± 1.4	0.0 ± 0.0														
			18:0_16:0(*sn*-2)_17:1	1.4 ± 1.4	0.0 ± 0.0														
			18:0_17:1(*sn*-2)_16:0	6.4 ± 5.7	0.1 ± 0.1														
		16:1_17:0_18:0	17:0_18:0(*sn*-2)_16:1	1.3 ± 1.8	0.0 ± 0.0														
			18:0_16:1(*sn*-2)_17:0	5.0 ± 4.9	0.1 ± 0.1														
			18:0_17:0(*sn*-2)_16:1	1.7 ± 1.9	0.0 ± 0.0														
	**51:2**			**0.7 ± 0.1**		**nd**		**nd**		**nd**		**nd**		**nd**		**nd**		**nd**	
		15:0_18:1_18:1	18:1_15:0(*sn*-2)_18:1	13.6 ± 21.1	0.1 ± 0.1														
			18:1_18:1(*sn*-2)_15:0	31.2 ± 21.0	0.2 ± 0.2														
		15:1_18:0_18:1	18:0_15:1(*sn*-2)_18:1	3.0 ± 4.2	0.0 ± 0.0														
			18:0_18:1(*sn*-2)_15:1	7.7 ± 11.0	0.1 ± 0.1														
			18:1_18:0(*sn*-2)_15:1	5.2 ± 7.3	0.0 ± 0.1														
		16:0_17:1_18:1	17:1_18:1(*sn*-2)_16:0	2.0 ± 4.0	0.0 ± 0.0														
			18:1_16:0(*sn*-2)_17:1	5.0 ± 9.9	0.0 ± 0.1														
			18:1_17:1(*sn*-2)_16:0	13.7 ± 9.4	0.1 ± 0.1														
		16:1_17:0_18:1	17:0_18:1(*sn*-2)_16:1	4.4 ± 8.8	0.0 ± 0.1														
			18:1_16:1(*sn*-2)_17:0	9.2 ± 10.6	0.1 ± 0.1														
			18:1_17:0(*sn*-2)_16:1	4.8 ± 9.6	0.0 ± 0.1														
		16:1_17:1_18:0	17:1_18:0(*sn*-2)_16:1	0.0 ± 0.0	0.0 ± 0.0														
			18:0_16:1(*sn*-2)_17:1	14.3 ± 2.2	0.1 ± 0.0														
			18:0_17:1(*sn*-2)_16:1	1.9 ± 2.7	0.0 ± 0.0														
**52**	**52:0**			**2.2 ± 0.1**		**0.3 ± 0.0**		**nd**		**0.4 ± 0.1**		**nd**		**nd**		**nd**		**nd**	
		16:0_16:0_20:0	16:0_20:0(*sn*-2)_16:0	1.8 ± 0.5	0.0 ± 0.0	3.2 ± 0.9	0.0 ± 0.0												
			20:0_16:0(*sn*-2)_16:0	3.8 ± 0.6	0.1 ± 0.0	6.0 ± 1.3	0.0 ± 0.0												
		16:0_18:0_18:0	18:0_16:0(*sn*-2)_18:0	85.4 ± 5.9	1.9 ± 0.3	70.2 ± 1.1	0.2 ± 0.0			25.1 ± 12.5	0.1 ± 0.1								
			18:0_18:0(*sn*-2)_16:0	9.0 ± 6.0	0.2 ± 0.1	20.6 ± 1.6	0.1 ± 0.0			74.9 ± 12.5	0.3 ± 0.1								
	**52:1**			**9.7 ± 0.1^{c}**		**1.9 ± 0.1**		**0.9 ± 0**		**1.2 ± 0.1**		**7.4 ± 0.3^{de}**		**3.5 ± 0.2**		**nd**		**4.6 ± 0.0**	
		16:0_16:0_20:1	16:0_20:1(*sn*-2)_16:0			0.1 ± 0.1	0.0 ± 0.0												
			20:1_16:0(*sn*-2)_16:0			2.9 ± 1.0	0.1 ± 0.0												
		16:0_18:0_18:1	18:0_16:0(*sn*-2)_18:1	77.7 ± 9.2	7.5 ± 1.0	78.5 ± 12.0	1.5 ± 0.3	71.8 ± 16.5	0.7 ± 0.2	55.3 ± 9.8	0.7 ± 0.1	95.5 ± 6.9	7.1 ± 0.8	74.1 ± 8.3	2.6 ± 0.4			79.8 ± 10.6	3.6 ± 0.5
			18:0_18:1(*sn*-2)_16:0	22.3 ± 9.2	2.2 ± 0.9	12.6 ± 11.0	0.2 ± 0.2	8.3 ± 4.9	0.1 ± 0.0	18.4 ± 8.4	0.2 ± 0.1	4.0 ± 7.1	0.3 ± 0.5	0.1 ± 0.1	0.0 ± 0.0			0.0 ± 0.0	0.0 ± 0.0
			18:1_18:0(*sn*-2)_16:0	0.0 ± 0.0	0.0 ± 0.0	5.9 ± 2.7	0.1 ± 0.1	19.9 ± 14.0	0.2 ± 0.1	26.4 ± 4.9	0.3 ± 0.1	0.5 ± 0.6	0.0 ± 0.0	25.8 ± 8.4	0.9 ± 0.3			20.2 ± 10.6	0.9 ± 0.5
	**52:2**			**10.6 ± 0.9^{b}**		**2.1 ± 0.2**		**1.9 ± 0.1**		**2.2 ± 0.1**		**23.8 ± 0.3^{a}**		**19.2 ± 0.4^{a}**		**4.8 ± 0.2^{c}**		**18.7 ± 0.2^{a}**	
		16:0_18:0_18:2	18:0_16:0(*sn*-2)_18:2	11.0 ± 1.7	1.2 ± 0.3									2.9 ± 3.2	0.6 ± 0.6				
			18:0_18:2(*sn*-2)_16:0	6.1 ± 1.7	0.6 ± 0.2									0.0 ± 0.0	0.0 ± 0.0				
			18:2_18:0(*sn*-2)_16:0	3.0 ± 1.9	0.3 ± 0.2									13.9 ± 3.0	2.7 ± 0.6				
		16:0_18:1_18:1	18:1_16:0(*sn*-2)_18:1	40.8 ± 9.5	4.3 ± 1.4	57.7 ± 10.0	1.2 ± 0.3	69.8 ± 8.0	1.3 ± 0.2	36.6 ± 11.4	0.8 ± 0.3	89.2 ± 9.2	21.3 ± 2.4	71.2 ± 1.4	13.7 ± 0.6	93.0 ± 1.5	4.5 ± 0.3	73.8 ± 19.4	13.8 ± 3.8
			18:1_18:1(*sn*-2)_16:0	19.8 ± 8.5	2.1 ± 1.1	42.3 ± 10.0	0.9 ± 0.3	30.2 ± 8.0	0.6 ± 0.2	63.4 ± 11.4	1.4 ± 0.3	10.8 ± 9.2	2.6 ± 2.2	0.0 ± 0.0	0.0 ± 0.0	7.0 ± 1.5	0.3 ± 0.1	26.2 ± 19.4	4.9 ± 3.7
		16:1_18:0_18:1	18:0_16:1(*sn*-2)_18:1	14.4 ± 3.9	1.5 ± 0.5									0.0 ± 0.0	0.0 ± 0.0				
			18:0_18:1(*sn*-2)_16:1	2.6 ± 1.3	0.3 ± 0.2									0.0 ± 0.0	0.0 ± 0.0				
			18:1_18:0(*sn*-2)_16:1	2.3 ± 1.8	0.2 ± 0.2									12.0 ± 1.6	2.3 ± 0.4				
	**52:3**			**1.8 ± 0.2**		**nd**		**0.7 ± 0.1**		**0.5 ± 0.1**		**7.8 ± 0.2^{d}**		**15.3 ± 0.4^{b}**		**3.2 ± 0.1**		**18.4 ± 0.4^{a}**	
		16:0_18:0_18:3	18:0_16:0(*sn*-2)_18:3	2.8 ± 1.5	0.1 ± 0.0					1.6 ± 0.2	0.0 ± 0.0							0.7 ± 0.4	0.1 ± 0.1
			18:0_18:3(*sn*-2)_16:0	6.7 ± 1.6	0.1 ± 0.0					1.8 ± 0.3	0.0 ± 0.0							0.3 ± 0.3	0.1 ± 0.1
			18:3_18:0(*sn*-2)_16:0	3.1 ± 2.8	0.1 ± 0.1					2.6 ± 0.8	0.0 ± 0.0							0.3 ± 0.4	0.1 ± 0.1
		16:0_18:1_18:2	18:1_16:0(*sn*-2)_18:2	24.7 ± 4.0	0.4 ± 0.1			20.5 ± 33.3	0.2 ± 0.3	21.7 ± 23.0	0.1 ± 0.1	82.2 ± 11.1	6.4 ± 1.1	65.0 ± 1.4	10.0 ± 0.5	40.1 ± 7.4	1.3 ± 0.3	68.0 ± 14.1	12.5 ± 2.9
			18:1_18:2(*sn*-2)_16:0	18.1 ± 2.2	0.3 ± 0.1			43.4 ± 22.7	0.3 ± 0.2	49.0 ± 19.7	0.3 ± 0.1	7.7 ± 12.4	0.6 ± 1.0	0.0 ± 0.0	0.0 ± 0.0	26.5 ± 7.6	0.8 ± 0.3	22.3 ± 16.5	4.1 ± 3.1
			18:2_18:1(*sn*-2)_16:0	6.5 ± 1.9	0.1 ± 0.0			18.5 ± 23.5	0.1 ± 0.2	10.2 ± 7.9	0.1 ± 0.0	4.6 ± 5.9	0.4 ± 0.5	0.0 ± 0.0	0.0 ± 0.0	1.1 ± 0.7	0.0 ± 0.0	1.0 ± 1.3	0.2 ± 0.2
		16:1_18:0_18:2	18:0_16:1(*sn*-2)_18:2	5.2 ± 3.9	0.1 ± 0.1			0.7 ± 0.7	0.0 ± 0.0	1.1 ± 1.0	0.0 ± 0.0	3.5 ± 1.3	0.3 ± 0.1	0.0 ± 0.0	0.0 ± 0.0			0.8 ± 0.4	0.1 ± 0.1
			18:0_18:2(*sn*-2)_16:1	1.9 ± 1.6	0.0 ± 0.0			2.9 ± 1.8	0.0 ± 0.0	3.3 ± 2.7	0.0 ± 0.0	0.3 ± 0.5	0.0 ± 0.0	0.0 ± 0.0	0.0 ± 0.0			0.7 ± 0.4	0.1 ± 0.1
			18:2_18:0(*sn*-2)_16:1	4.2 ± 4.6	0.1 ± 0.1			2.5 ± 3.0	0.0 ± 0.0	1.4 ± 1.3	0.0 ± 0.0	1.1 ± 0.8	0.1 ± 0.1	35.0 ± 1.4	5.4 ± 0.4			1.4 ± 1.3	0.3 ± 0.2
		16:1_18:1_18:1	18:1_16:1(*sn*-2)_18:1	11.7 ± 10.8	0.2 ± 0.2			6.8 ± 7.9	0.1 ± 0.1	6.4 ± 8.8	0.0 ± 0.1	3.6 ± 4.8	0.3 ± 0.4			16.6 ± 12.1	0.5 ± 0.4	5.5 ± 3.0	1.0 ± 0.6
			18:1_18:1(*sn*-2)_16:1	15.0 ± 9.2	0.3 ± 0.2			10.5 ± 5.7	0.1 ± 0.1	9.3 ± 5.6	0.1 ± 0.0	1.2 ± 1.8	0.1 ± 0.1			15.7 ± 10.9	0.5 ± 0.4	7.2 ± 3.7	1.3 ± 0.7

ACN	TG species	TG molecular species	TG regioisomer	Camel		Cow		Goat		Sheep		Human		Dog		Horse		Pig	
				in TG species	of total TGs	in TG species	of total TGs	in TG species	of total TGs	in TG species	of total TGs	in TG species	of total TGs	in TG species	of total TGs	in TG species	of total TGs	in TG species	of total TGs
	**52:4**			**0.7 ± 0.1**		**nd**		**nd**		**nd**		**1.5 ± 0.1**		**5.4 ± 0.3**[e]		**6.4 ± 0.4**[a]		**8.1 ± 0.1**[c]	
		16:0_16:0_20:4	16:0_20:4(*sn*-2)_16:0	0.0 ± 0.0	0.0 ± 0.0													4.2 ± 1.3	0.3 ± 0.1
			20:4_16:0(*sn*-2)_16:0	9.2 ± 4.0	0.1 ± 0.0													1.4 ± 0.1	0.1 ± 0.0
		16:0_18:1_18:3	18:1_16:0(*sn*-2)_18:3	18.4 ± 14.8	0.1 ± 0.1							67.7 ± 4.5	1.0 ± 0.1	2.3 ± 3.2	0.1 ± 0.2	58.8 ± 11.0	3.8 ± 0.9		
			18:1_18:3(*sn*-2)_16:0	14.3 ± 18.6	0.1 ± 0.1							0.0 ± 0.0	0.0 ± 0.0	0.0 ± 0.0	0.0 ± 0.0	24.2 ± 11.7	1.6 ± 0.8		
			18:3_18:1(*sn*-2)_16:0	2.8 ± 5.6	0.0 ± 0.0							0.0 ± 0.1	0.0 ± 0.0	23.7 ± 5.7	1.3 ± 0.4	0.1 ± 0.1	0.0 ± 0.0		
		16:0_18:2_18:2	18:2_16:0(*sn*-2)_18:2	6.8 ± 7.8	0.0 ± 0.1							32.2 ± 4.5	0.5 ± 0.1	28.9 ± 3.1	1.6 ± 0.3	5.2 ± 3.0	0.3 ± 0.2	17.6 ± 4.0	1.4 ± 0.3
			18:2_18:2(*sn*-2)_16:0	7.5 ± 8.9	0.1 ± 0.1							0.0 ± 0.0	0.0 ± 0.0	0.0 ± 0.0	0.0 ± 0.0	0.1 ± 0.2	0.0 ± 0.0	32.3 ± 6.1	2.6 ± 0.5
		16:1_18:0_18:3	18:0_16:1(*sn*-2)_18:3	8.4 ± 3.6	0.1 ± 0.0														
			18:0_18:3(*sn*-2)_16:1	0.6 ± 1.2	0.0 ± 0.0														
			18:3_18:0(*sn*-2)_16:1	1.2 ± 2.5	0.0 ± 0.0														
		16:1_18:1_18:2	18:1_16:1(*sn*-2)_18:2	31.2 ± 13.7	0.2 ± 0.1									0.0 ± 0.0	0.0 ± 0.0	15.4 ± 1.3	1.0 ± 0.1	44.1 ± 4.4	3.6 ± 0.4
			18:1_18:2(*sn*-2)_16:1	2.6 ± 5.2	0.0 ± 0.0									0.0 ± 0.0	0.0 ± 0.0	0.0 ± 0.1	0.0 ± 0.0	0.2 ± 0.4	0.0 ± 0.0
			18:2_18:1(*sn*-2)_16:1	1.5 ± 3.1	0.0 ± 0.0									45.1 ± 1.2	2.4 ± 0.2	0.0 ± 0.1	0.0 ± 0.0	0.2 ± 0.3	0.0 ± 0.0
	**52:5**			**nd**		**nd**		**nd**		**nd**		**nd**		**nd**		**0.9 ± 0.0**		**0.9 ± 0.0**	
		16:0_18:2_18:3	18:2_16:0(*sn*-2)_18:3													31.2 ± 18.0	0.3 ± 0.2	7.8 ± 8.9	0.1 ± 0.1
			18:2_18:3(*sn*-2)_16:0													6.6 ± 6.1	0.1 ± 0.1	21.5 ± 24.9	0.2 ± 0.2
			18:3_18:2(*sn*-2)_16:0													4.8 ± 6.4	0.0 ± 0.1	10.3 ± 13.5	0.1 ± 0.1
		16:1_18:1_18:3	18:1_16:1(*sn*-2)_18:3													41.3 ± 16.7	0.4 ± 0.2	4.0 ± 6.0	0.0 ± 0.1
			18:1_18:3(*sn*-2)_16:1													3.0 ± 4.5	0.0 ± 0.0	2.8 ± 3.5	0.0 ± 0.0
			18:3_18:1(*sn*-2)_16:1													9.2 ± 13.8	0.1 ± 0.1	10.5 ± 8.6	0.1 ± 0.1
		16:1_18:2_18:2	18:2_16:1(*sn*-2)_18:2													4.5 ± 0.4	0.0 ± 0.0	31.9 ± 36.9	0.3 ± 0.3
			18:2_18:2(*sn*-2)_16:1													1.2 ± 1.3	0.0 ± 0.0	25.4 ± 17.8	0.2 ± 0.2
	**52:6**			**nd**		**nd**		**nd**		**nd**		**nd**		**nd**		**1.3 ± 0.1**		**nd**	
		16:0_18:3_18:3	18:3_16:0(*sn*-2)_18:3													42.3 ± 9.8	0.5 ± 0.2		
			18:3_18:3(*sn*-2)_16:0													28.4 ± 6.0	0.4 ± 0.1		
		16:1_18:2_18:3	18:2_16:1(*sn*-2)_18:3													7.3 ± 10.4	0.1 ± 0.1		
			18:2_18:3(*sn*-2)_16:1													1.4 ± 1.4	0.0 ± 0.0		
			18:3_18:2(*sn*-2)_16:1													20.6 ± 12.7	0.3 ± 0.2		
**54**	**54:1**			**1.7 ± 0.1**		**0.4 ± 0.1**		**nd**		**0.4 ± 0.1**		**nd**		**nd**		**nd**		**nd**	
		16:0_18:1_20:0	18:1_20:0(*sn*-2)_16:0	2.7 ± 2.9	0.0 ± 0.1														
			20:0_16:0(*sn*-2)_18:1	2.6 ± 3.0	0.0 ± 0.1														
			20:0_18:1(*sn*-2)_16:0	5.6 ± 1.7	0.1 ± 0.0														
		18:0_18:0_18:1	18:0_18:0(*sn*-2)_18:1	31.5 ± 9.0	0.5 ± 0.2	88.2 ± 13.5	0.4 ± 0.1			80.7 ± 14.1	0.4 ± 0.1								
			18:0_18:1(*sn*-2)_18:0	57.6 ± 9.9	1.0 ± 0.2	11.9 ± 13.5	0.0 ± 0.1			19.3 ± 14.1	0.1 ± 0.1								
	**54:2**			**3.6 ± 0.4**		**0.4 ± 0.0**		**0.4 ± 0.0**		**0.8 ± 0.1**		**1.9 ± 0.0**		**0.8 ± 0.1**		**nd**		**2.2 ± 0.1**	
		16:0_18:1_20:1	18:1_20:1(*sn*-2)_16:0	0.1 ± 0.1	0.0 ± 0.0							1.1 ± 0.1	0.0 ± 0.0	0.2 ± 0.3	0.0 ± 0.0				
			20:1_16:0(*sn*-2)_18:1	0.2 ± 0.4	0.0 ± 0.0							18.0 ± 2.1	0.3 ± 0.0	24.0 ± 4.3	0.2 ± 0.1				
			20:1_18:1(*sn*-2)_16:0	3.3 ± 0.5	0.1 ± 0.0							3.0 ± 1.3	0.1 ± 0.0	0.6 ± 1.0	0.0 ± 0.0				
		18:0_18:0_18:2	18:0_18:0(*sn*-2)_18:2											15.1 ± 2.1	0.1 ± 0.0				
			18:0_18:2(*sn*-2)_18:0											0.0 ± 0.0	0.0 ± 0.0				
		18:0_18:1_18:1	18:0_18:1(*sn*-2)_18:1	96.4 ± 0.6	3.5 ± 0.4	82.8 ± 7.4	0.4 ± 0.1	83.7 ± 14.5	0.3 ± 0.1	69.4 ± 19.1	0.5 ± 0.2	62.4 ± 12.0	1.2 ± 0.3	20.3 ± 23.4	0.2 ± 0.2			91.2 ± 8.8	2.0 ± 0.3
			18:1_18:0(*sn*-2)_18:1	0.0 ± 0.1	0.0 ± 0.0	17.2 ± 7.4	0.1 ± 0.0	16.3 ± 14.5	0.1 ± 0.1	30.6 ± 19.1	0.2 ± 0.2	15.5 ± 12.5	0.3 ± 0.2	53.6 ± 24.8	0.4 ± 0.3			8.8 ± 8.8	0.2 ± 0.2
	**54:3**			**1.9 ± 0.3**		**0.4 ± 0.0**		**0.6 ± 0.1**		**0.4 ± 0.0**		**0.7 ± 0.2**		**4.3 ± 0.3**		**0.4 ± 0.1**		**7.3 ± 0.3**[d]	
		16:0_18:1_20:2	18:1_20:2(*sn*-2)_16:0											0.0 ± 0.0	0.0 ± 0.0			3.1 ± 3.2	0.2 ± 0.2
			20:2_16:0(*sn*-2)_18:1											7.2 ± 2.1	0.3 ± 0.1			2.4 ± 2.6	0.2 ± 0.2
			20:2_18:1(*sn*-2)_16:0											0.0 ± 0.0	0.0 ± 0.0			0.6 ± 0.4	0.0 ± 0.0
		16:0_18:2_20:1	18:2_20:1(*sn*-2)_16:0											1.3 ± 2.3	0.1 ± 0.1				
			20:1_16:0(*sn*-2)_18:2											3.9 ± 1.2	0.2 ± 0.1				
			20:1_18:2(*sn*-2)_16:0											0.0 ± 0.0	0.0 ± 0.0				
		18:0_18:0_18:3	18:0_18:0(*sn*-2)_18:3	3.9 ± 0.9	0.1 ± 0.0														
			18:0_18:3(*sn*-2)_18:0	1.1 ± 0.8	0.0 ± 0.0														
		18:0_18:1_18:2	18:0_18:1(*sn*-2)_18:2	4.4 ± 3.8	0.1 ± 0.1	0.5 ± 0.7	0.0 ± 0.0	0.9 ± 1.2	0.0 ± 0.0			6.9 ± 2.7	0.0 ± 0.0	0.0 ± 0.0	0.0 ± 0.0				
			18:0_18:2(*sn*-2)_18:1	36.2 ± 7.0	0.7 ± 0.2	17.2 ± 10.7	0.1 ± 0.0	11.1 ± 14.1	0.1 ± 0.1			30.7 ± 5.8	0.2 ± 0.1	0.0 ± 0.0	0.0 ± 0.0				
			18:1_18:0(*sn*-2)_18:2	3.6 ± 3.3	0.1 ± 0.1	6.9 ± 9.7	0.0 ± 0.0	9.9 ± 14.0	0.1 ± 0.1			1.9 ± 0.2	0.0 ± 0.0	42.5 ± 2.8	1.8 ± 0.2				
		18:1_18:1_18:1	18:1_18:1(*sn*-2)_18:1	52.0 ± 1.7	1.0 ± 0.2	87.7 ± 14.2	0.3 ± 0.1	89.1 ± 12.6	0.5 ± 0.1	100.0 ± 0.0	0.4 ± 0.0	80.3 ± 22.8	0.5 ± 0.3	45.1 ± 3.4	1.9 ± 0.3	100.0 ± 0.0	0.4 ± 0.1	94.0 ± 0.7	6.9 ± 0.4

ACN	TG species	TG molecular species	TG regioisomer	Camel		Cow		Goat		Sheep		Human		Dog		Horse		Pig	
				in TG species	of total TGs	in TG species	of total TGs	in TG species	of total TGs	in TG species	of total TGs	in TG species	of total TGs	in TG species	of total TGs	in TG species	of total TGs	in TG species	of total TGs
	**54:4**			**0.8 ± 0.1**		**nd**		**nd**		**nd**		**nd**		**5.2 ± 0.3**[e]		**0.5 ± 0.1**		**10.3 ± 0.1**[b]	
		16:0_18:1_20:3	18:1_20:3(*sn*-2)_16:0															0.6 ± 0.9	0.1 ± 0.1
			20:3_16:0(*sn*-2)_18:1															3.8 ± 2.3	0.4 ± 0.2
			20:3_18:1(*sn*-2)_16:0															0.7 ± 0.8	0.1 ± 0.1
		16:0_18:2_20:2	18:2_20:2(*sn*-2)_16:0															0.3 ± 0.3	0.0 ± 0.0
			20:2_16:0(*sn*-2)_18:2															2.3 ± 1.4	0.2 ± 0.1
			20:2_18:2(*sn*-2)_16:0															0.5 ± 0.6	0.0 ± 0.1
		18:0_18:1_18:3	18:0_18:1(*sn*-2)_18:3											0.0 ± 0.0	0.0 ± 0.0				
			18:0_18:3(*sn*-2)_18:1											0.0 ± 0.0	0.0 ± 0.0				
			18:1_18:0(*sn*-2)_18:3											2.7 ± 1.4	0.1 ± 0.1				
		18:0_18:2_18:2	18:0_18:2(*sn*-2)_18:2											0.0 ± 0.0	0.0 ± 0.0			7.6 ± 3.1	0.8 ± 0.3
			18:2_18:0(*sn*-2)_18:2											29.6 ± 4.4	1.6 ± 0.3			5.6 ± 2.6	0.6 ± 0.3
		18:1_18:1_18:2	18:1_18:1(*sn*-2)_18:2											67.7 ± 3.3	3.5 ± 0.4	36.5 ± 28.7	0.2 ± 0.2	22.6 ± 6.9	2.3 ± 0.7
			18:1_18:2(*sn*-2)_18:1											0.0 ± 0.0	0.0 ± 0.0	63.5 ± 28.7	0.3 ± 0.2	56.0 ± 5.1	5.7 ± 0.6
	**54:5**			**nd**		**nd**		**nd**		**nd**		**nd**		**2.6 ± 0.6**		**0.8 ± 0.1**		**5.7 ± 0.1**[e]	
		16:0_18:1_20:4	18:1_20:4(*sn*-2)_16:0															0.0 ± 0.0	0.0 ± 0.0
			20:4_16:0(*sn*-2)_18:1															17.1 ± 0.8	1.0 ± 0.1
			20:4_18:1(*sn*-2)_16:0															0.0 ± 0.0	0.0 ± 0.0
		18:1_18:1_18:3	18:1_18:1(*sn*-2)_18:3											22.3 ± 2.9	0.6 ± 0.2	58.7 ± 16.5	0.4 ± 0.2		
			18:1_18:3(*sn*-2)_18:1											0.0 ± 0.0	0.0 ± 0.0	32.5 ± 11.1	0.2 ± 0.1		
		18:1_18:2_18:2	18:1_18:2(*sn*-2)_18:2											0.0 ± 0.0	0.0 ± 0.0	6.8 ± 1.8	0.1 ± 0.0	78.4 ± 8.6	4.5 ± 0.6
			18:2_18:1(*sn*-2)_18:2											77.7 ± 2.9	2.0 ± 0.5	6.4 ± 2.2	0.0 ± 0.0	4.5 ± 7.8	0.3 ± 0.4
	**54:6**			**nd**		**nd**		**nd**		**nd**		**nd**		**nd**		**0.7 ± 0.1**		**1.5 ± 0.1**	
		18:1_18:2_18:3	18:1_18:2(*sn*-2)_18:3													68.8 ± 24.0	0.5 ± 0.2		
			18:1_18:3(*sn*-2)_18:2													15.1 ± 13.4	0.1 ± 0.1		
			18:2_18:1(*sn*-2)_18:3													16.2 ± 32.4	0.1 ± 0.2		
		18:2_18:2_18:2	18:2_18:2(*sn*-2)_18:2															100.0 ± 0.0	1.5 ± 0.1
	**54:7**			**nd**		**nd**		**nd**		**nd**		**nd**		**nd**		**0.6 ± 0.4**		**nd**	
		18:1_18:3_18:3	18:1_18:3(*sn*-2)_18:3													53.4 ± 14.7	0.3 ± 0.3		
			18:3_18:1(*sn*-2)_18:3													25.4 ± 13.3	0.1 ± 0.2		
		18:2_18:2_18:3	18:2_18:2(*sn*-2)_18:3													10.0 ± 2.4	0.1 ± 0.1		
			18:2_18:3(*sn*-2)_18:2													11.2 ± 2.2	0.1 ± 0.1		

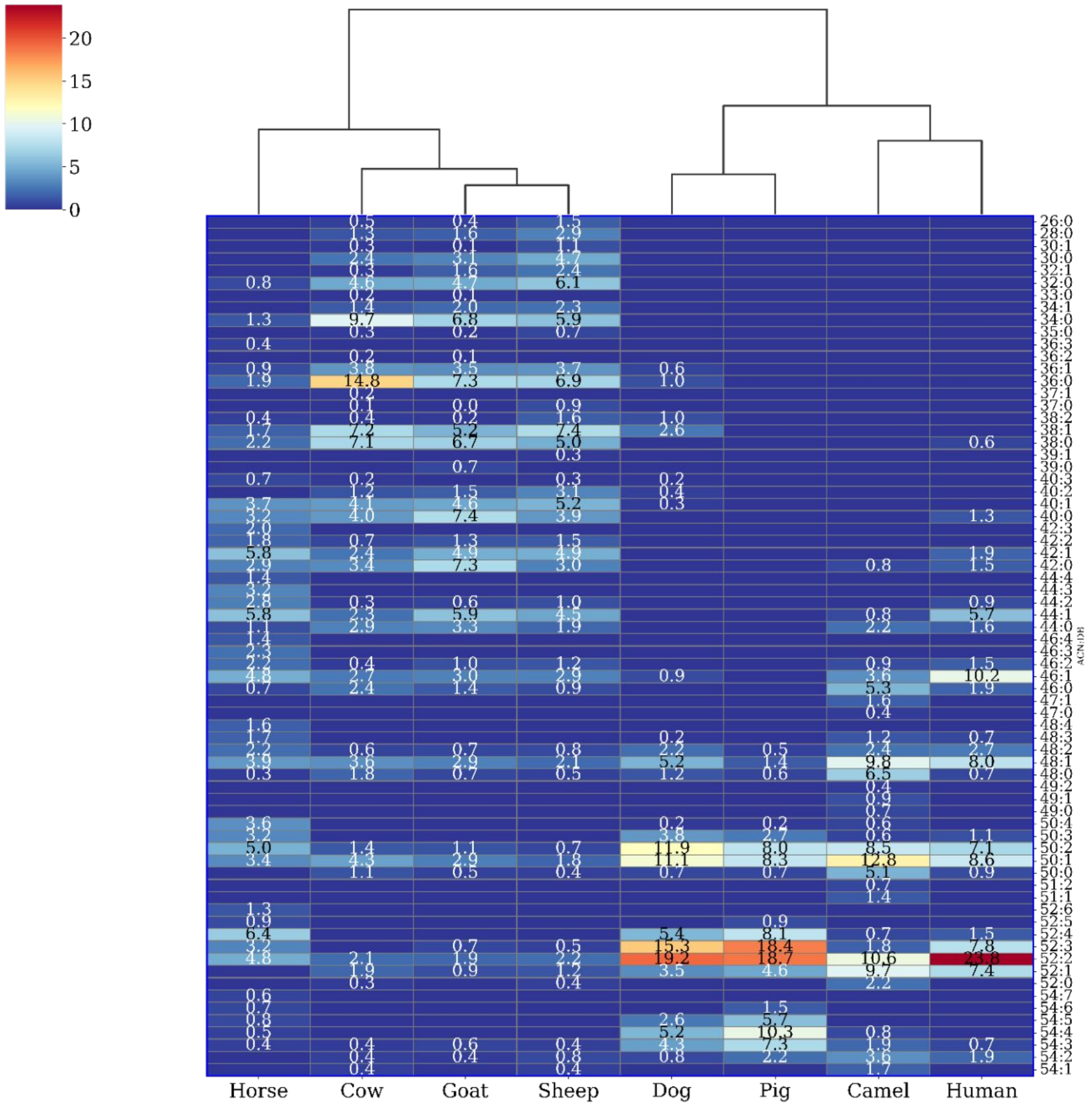

Figure S2 Clustering analysis of TG species composition of mammalian milk (mol%).

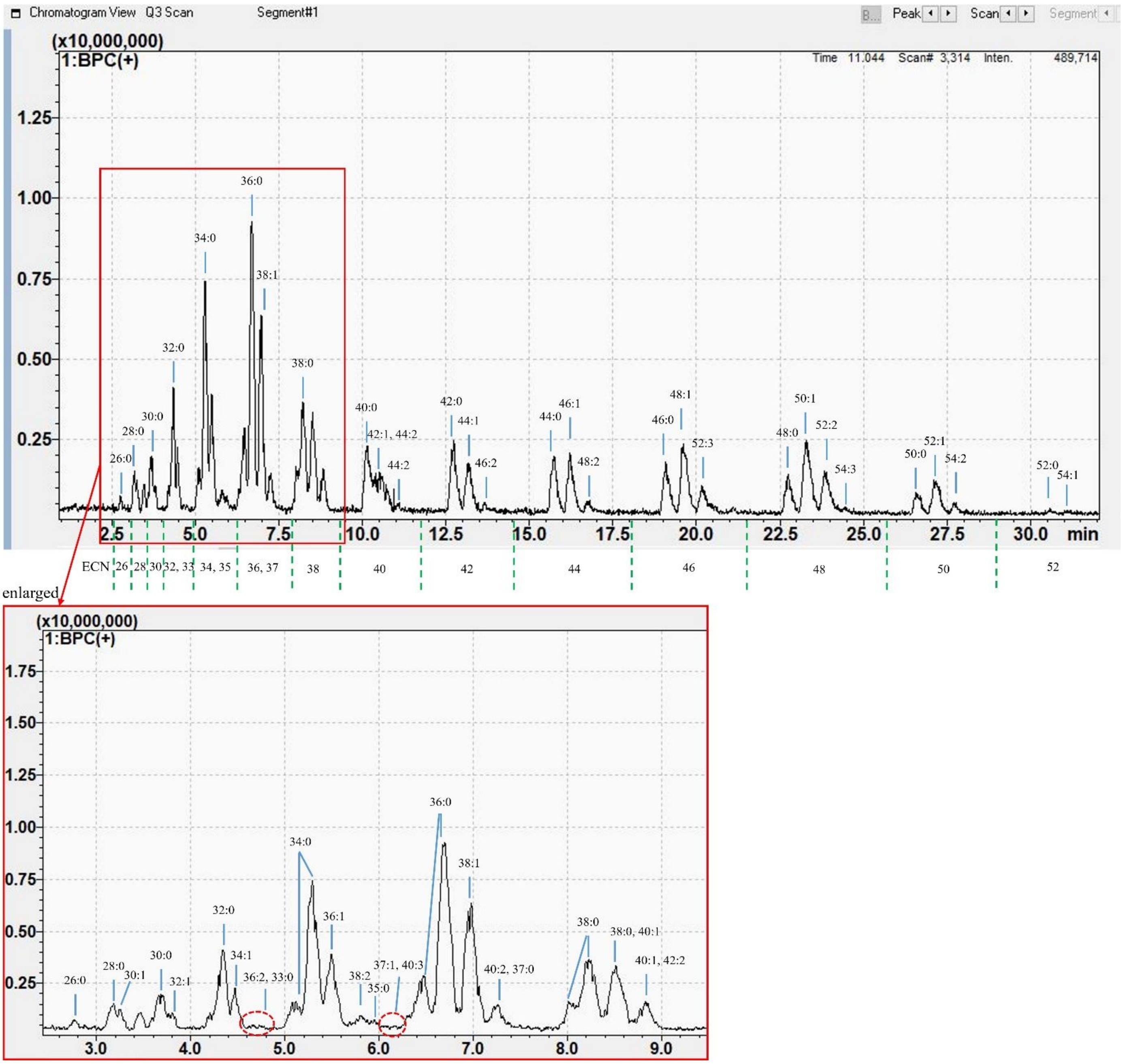

Figure S3 MS scan chromatogram of cow milk TG species

Table S6 Retention times (tR) of TG species.

Start–end (min)	precursor ion	ACN:DB	ECN	Start–end (min)	precursor ion	ACN:DB	ECN
2.485–3.086	516.40	26:0	26	13.463–14.919	868.65	52:6	40
				14.169–15.573	894.70	54:7	40
3.031–3.415	544.45	28:0	28				
3.123–3.577	570.40	30:1	28	12.860–13.730	740.65	42:0	42
				13.283–14.333	766.65	44:1	42
3.633–4.127	572.45	30:0	30	13.587–14.857	792.60	46:2	42
3.683–4.170	599.55	32:1	30	13.899–15.643	818.70	48:3	42
2.176–4.459	650.55	36:3	30	14.867–16.823	844.70	50:4	42
				15.476–17.063	870.65	52:5	42
4.293–4.960	600.55	32:0	32	16.379–17.873	896.65	54:6	42
4.543–5.307	626.50	34:1	32				
4.643–5.400	652.55	36:2	32	15.600–16.840	768.65	44:0	44
				16.213–17.227	794.65	46:1	44
4.733–5.603	614.55	33:0	33	16.507–18.097	820.70	48:2	44
				16.947–19.457	846.70	50:3	44
3.487–3.767	680.50	38:2	34	18.743–20.169	898.75	54:5	44
5.360–6.070	628.50	34:0	34				
5.573–6.373	654.55	36:1	34	17.083–19.32	808.70	47:1	45
5.780–6.653	680.55	38:2	34	17.453–19.767	834.70	49:2	45
6.320–7.007	706.65	40:3	34				
				18.760–21.090	822.70	48:1	46
5.857–6.693	642.55	35:0	35	18.940–20.220	796.70	46:0	46
6.250–7.223	668.60	37:1	35	19.323–20.673	822.70	48:1	46
				19.747–21.707	848.70	50:2	46
6.663–7.487	656.55	36:0	36	20.337–23.007	874.70	52:3	46
6.970–7.910	682.55	38:1	36	21.283–25.443	900.90	54:4	46
7.186–8.366	734.60	42:3	36				
7.273–8.217	708.60	40:2	36	20.473–23.237	836.70	49:1	47
7.643–8.513	760.60	44:4	36	21.067–24.273	862.75	51:2	47
7.243–8.503	696.60	39:1	37	22.443–23.617	824.70	48:0	48
7.350–8.290	670.60	37:0	37	22.887–24.220	850.75	50:1	48
				23.423–25.476	876.85	52:2	48
8.177–9.400	684.60	38:0	38	23.880–25.727	902.75	54:3	48
8.543–9.703	710.60	40:1	38				
8.987–9.947	736.60	42:2	38	23.760–25.743	838.75	49:0	49
9.063–10.203	762.60	44:3	38	24.213–26.583	864.75	51:1	49
9.606–10.446	788.65	46:4	38				
				26.150–27.560	852.75	50:0	50
8.447–9.76	698.60	39:0	39	26.603–27.983	878.70	52:1	50
				27.167–29.067	904.90	54:2	50
10.310–11.530	712.60	40:0	40				
10.693–11.613	738.60	42:1	40	30.060–31.460	880.75	52:0	52
11.097–12.137	764.65	44:2	40	30.397–32.147	906.85	54:1	52
11.446–12.639	790.60	46:3	40				
11.983–13.129	816.65	48:4	40				

Figure S4 Examples of MS/MS chromatogram and spectra. (a, TGs 52:0, 52:1, 52:2, 52:3, 52:4, and 52:5 in cow and pig milk; b, TGs 54:2 and 44:0 in cow milk; b, odd-chain TGs 49:0 and 51:1 in camel milk)

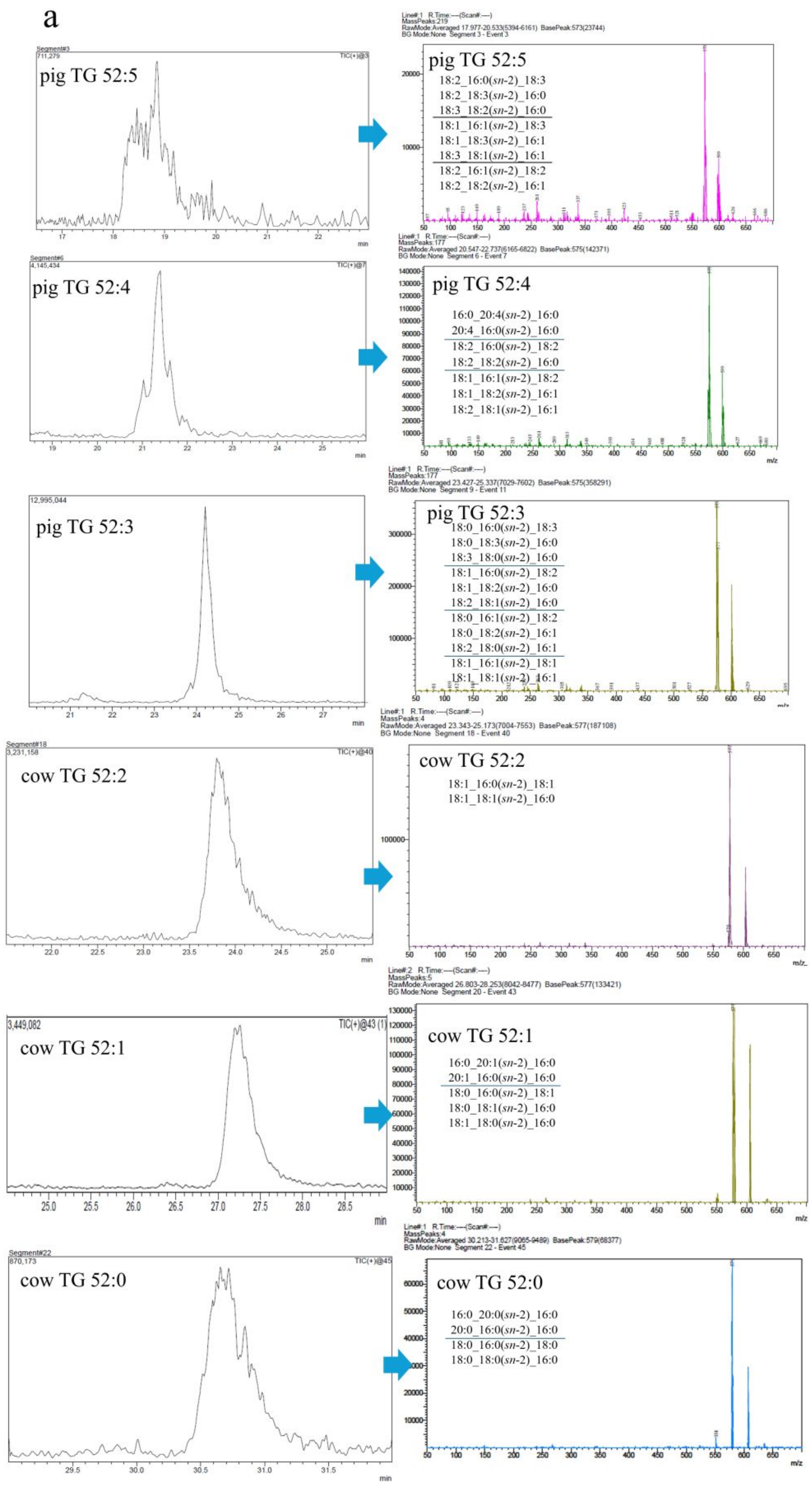

b

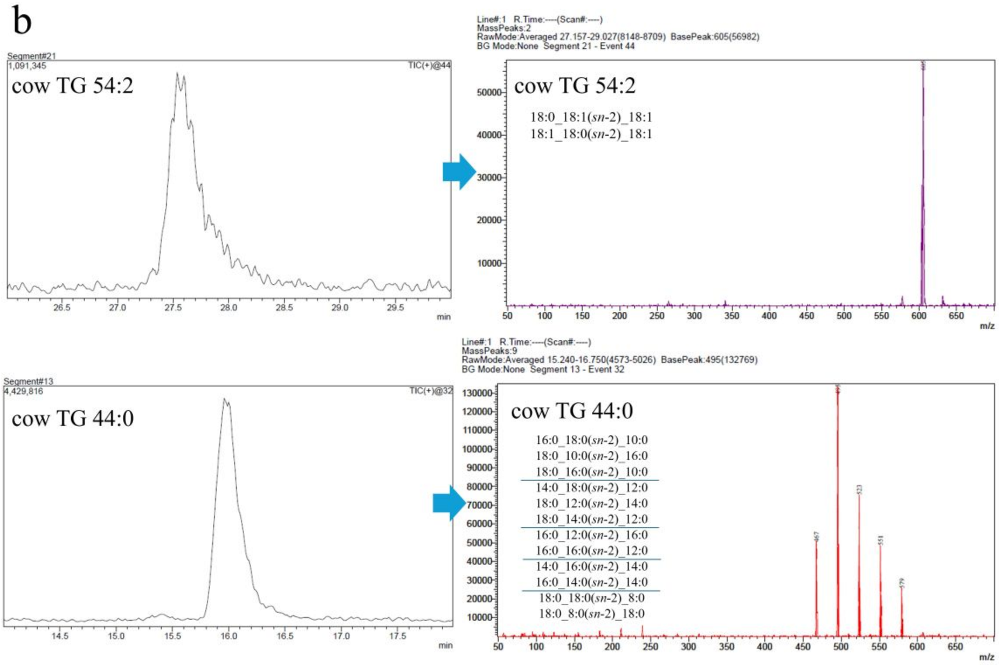
cow TG 54:2
cow TG 54:2
18:0_18:1(sn-2)_18:1
18:1_18:0(sn-2)_18:1
cow TG 44:0
cow TG 44:0
16:0_18:0(sn-2)_10:0
18:0_10:0(sn-2)_16:0
18:0_16:0(sn-2)_10:0
14:0_18:0(sn-2)_12:0
18:0_12:0(sn-2)_14:0
18:0_14:0(sn-2)_12:0
16:0_12:0(sn-2)_16:0
16:0_16:0(sn-2)_12:0
14:0_16:0(sn-2)_14:0
16:0_14:0(sn-2)_14:0
18:0_18:0(sn-2)_8:0
18:0_8:0(sn-2)_18:0
m/z
min

c

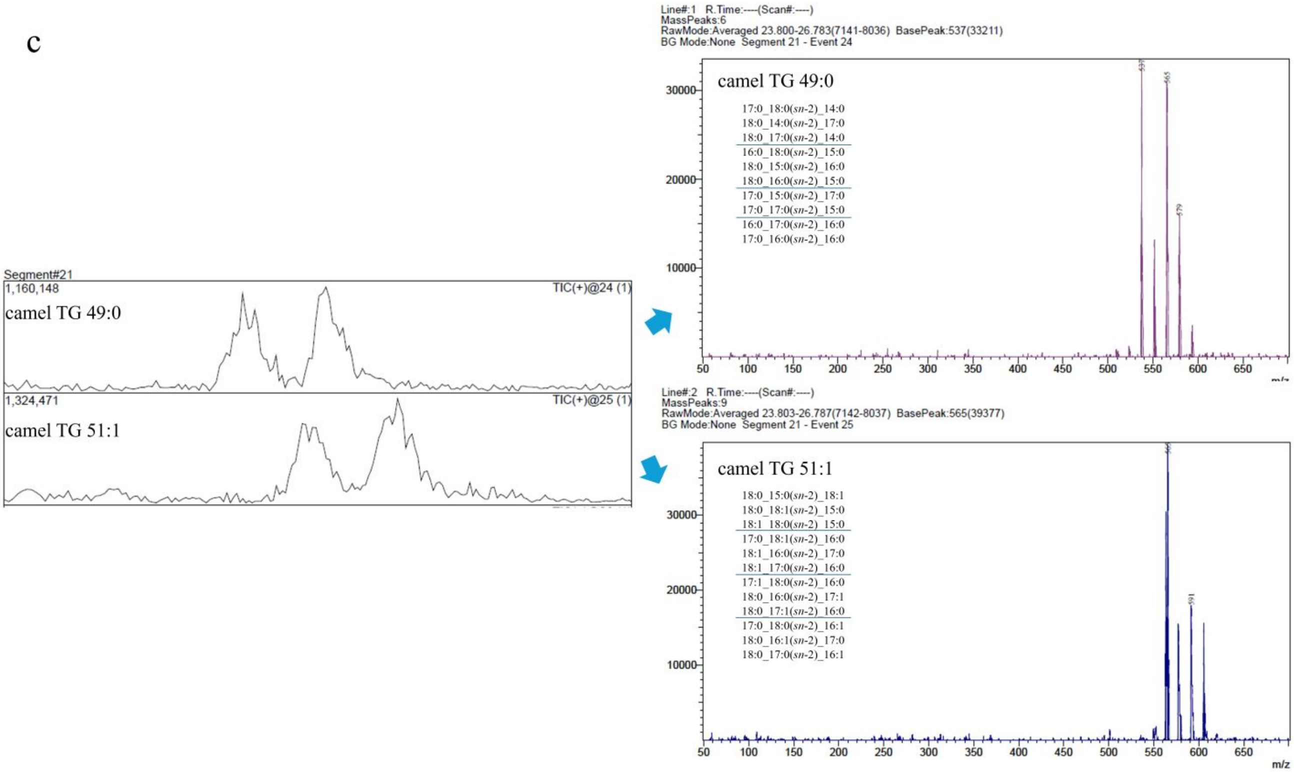
camel TG 49:0
camel TG 51:1
camel TG 49:0
17:0_18:0(sn-2)_14:0
18:0_14:0(sn-2)_17:0
18:0_17:0(sn-2)_14:0
16:0_18:0(sn-2)_15:0
18:0_15:0(sn-2)_16:0
18:0_16:0(sn-2)_15:0
17:0_15:0(sn-2)_17:0
17:0_17:0(sn-2)_15:0
16:0_17:0(sn-2)_16:0
17:0_16:0(sn-2)_16:0
camel TG 51:1
18:0_15:0(sn-2)_18:1
18:0_18:1(sn-2)_15:0
18:1_18:0(sn-2)_15:0
17:0_18:1(sn-2)_16:0
18:1_16:0(sn-2)_17:0
18:1_17:0(sn-2)_16:0
17:1_18:0(sn-2)_16:0
18:0_16:0(sn-2)_17:1
18:0_17:1(sn-2)_16:0
17:0_18:0(sn-2)_16:1
18:0_16:1(sn-2)_17:0
18:0_17:0(sn-2)_16:1
m/z

Table S7 The *sn*-positional features of FAs in detected TG regioisomers in eight mammalian milk species (mol%).

FA	*sn* position	Pseudo-ruminant	Ruminants			Non-ruminants			
		Camel	Cow	Goat	Sheep	Human	Dog	Horse	Pig
4:0	*sn*-1,3	0.0	10.1	4.2	5.9	0.0	1.7	0.4	0.0
	sn-2	0.0	4.4	4.0	6.2	0.0	0.2	0.1	0.0
6:0	*sn*-1,3	0.0	4.5	3.5	3.9	0.0	0.1	0.2	0.0
	sn-2	0.0	1.2	2.1	2.7	0.0	0.0	0.2	0.0
8:0	*sn*-1,3	0.0	1.6	2.8	2.8	0.0	0.0	2.7	0.0
	sn-2	0.0	1.0	1.8	2.3	0.0	0.0	1.3	0.0
10:0	*sn*-1,3	0.0	2.8	10.3	8.3	0.8	0.0	6.1	0.0
	sn-2	0.1	1.3	2.4	2.6	0.8	0.0	2.3	0.0
12:0	*sn*-1,3	0.5	2.2	3.8	4.2	5.3	0.1	5.5	0.0
	sn-2	1.2	2.0	2.1	1.4	3.1	0.0	2.7	0.0
14:0	*sn*-1,3	6.0	6.8	6.2	6.2	4.4	3.1	4.5	0.4
	sn-2	8.7	5.8	5.4	2.9	5.7	0.1	4.9	1.1
14:1	*sn*-1,3	0.3	0.4	0.1	0.0	0.0	0.2	0.2	0.0
	sn-2	0.1	0.2	0.0	0.0	0.0	0.0	0.1	0.0
15:0	*sn*-1,3	0.6	0.2	0.1	0.3	0.0	0.0	0.0	0.0
	sn-2	1.1	0.1	0.2	0.1	0.0	0.0	0.0	0.0
15:1	*sn*-1,3	0.1	0.0	0.0	0.0	0.0	0.0	0.0	0.0
	sn-2	0.0	0.0	0.0	0.0	0.0	0.0	0.0	0.0
16:0	*sn*-1,3	16.8	20.4	17.5	14.7	8.9	7.7	10.3	10.3
	sn-2	11.6	10.3	8.2	4.5	18.5	20.8	13.5	16.2
16:1	*sn*-1,3	4.0	0.4	0.6	0.4	0.9	10.0	6.2	2.2
	sn-2	1.8	0.4	0.4	0.5	0.9	0.0	2.8	2.0
17:0	*sn*-1,3	0.4	0.0	0.1	0.3	0.0	0.0	0.0	0.0
	sn-2	0.2	0.0	0.0	0.1	0.0	0.0	0.0	0.0
17:1	*sn*-1,3	0.1	0.0	0.0	0.0	0.0	0.0	0.0	0.0
	sn-2	0.1	0.0	0.0	0.0	0.0	0.0	0.0	0.0
18:0	*sn*-1,3	15.1	5.7	3.8	5.1	5.5	1.6	0.2	2.6
	sn-2	2.7	3.0	2.6	2.7	0.9	7.2	0.2	0.9
18:1	*sn*-1,3	20.0	11.1	13.1	13.9	36.3	25.8	19.1	36.5
	sn-2	4.6	3.5	3.9	6.8	2.9	5.0	2.6	6.3
18:2	*sn*-1,3	2.2	0.4	0.6	0.6	4.1	15.4	3.5	13.8
	sn-2	0.9	0.2	0.3	0.4	0.6	0.0	1.1	6.6
18:3	*sn*-1,3	0.5	0.0	0.0	0.0	0.4	0.8	7.9	0.2
	sn-2	0.1	0.0	0.0	0.0	0.0	0.0	1.4	0.1
20:0	*sn*-1,3	0.1	0.0	0.0	0.0	0.0	0.0	0.0	0.0
	sn-2	0.0	0.0	0.0	0.0	0.0	0.0	0.0	0.0
20:1	*sn*-1,3	0.0	0.0	0.0	0.0	0.1	0.1	0.0	0.0
	sn-2	0.0	0.0	0.0	0.0	0.0	0.0	0.0	0.0
20:2	*sn*-1,3	0.0	0.0	0.0	0.0	0.0	0.1	0.0	0.2
	sn-2	0.0	0.0	0.0	0.0	0.0	0.0	0.0	0.1
20:3	*sn*-1,3	0.0	0.0	0.0	0.0	0.0	0.0	0.0	0.2
	sn-2	0.0	0.0	0.0	0.0	0.0	0.0	0.0	0.0
20:4	*sn*-1,3	0.0	0.0	0.0	0.0	0.0	0.0	0.0	0.4
	sn-2	0.0	0.0	0.0	0.0	0.0	0.0	0.0	0.1

Formula S1 Calculation of the number of theoretically possible triacylglycerol regioisomers.

The number of combinations theoretically possible triacylglycerol regioisomers considering the equivalence of the *sn*-1 and *sn*-3 positions is calculated as follow.

x is the number of FAs

1. the number of possibilities in sn-2 position:

$$x$$

2. the number of possibilities in *sn*-1/3 positions (repetition combination):

$$\binom{n+k-1}{k} = x\binom{x+2-1}{2}$$

where n is the number of FAs ($n = x$), k is the number of items ($k = 2$)

3. the number of theoretically possible triacylglycerol regioisomers

$$y = x\binom{x+2-1}{2} = \frac{x^3 + x^2}{2}$$

For example, for FA $x = 10$, TG regioisomers $y = 550$.
